# Vitamin D3 reduces the viability of cancer cells *in vitro* and retard the EAC tumors growth in mice

**DOI:** 10.1371/journal.pone.0331306

**Published:** 2025-09-08

**Authors:** Vidya G. Bettada, Chaithanya G. Basavaraju, Shalini H. Doreswamy, SubbaRao V. Tulimilli, Rimshia Naaz, Siva Dallavalasa, Paramahans V. Salimath, Anjali Devi S. Bettadapura, Asha Srinivasan, Rajalakshmi Ramashetty, Suma M. Natraj, SubbaRao V. Madhunapantula

**Affiliations:** 1 Center of Excellence in Molecular Biology and Regenerative Medicine (CEMR) Laboratory (DST-FIST supported center, ICMR collaborating center of excellence – ICMR-CCoE), Department of Biochemistry (DST-FIST supported department), JSS Medical College, JSS Academy of Higher Education & Research (JSS AHER), Mysuru, Karnataka, India; 2 Division of Nanoscience and Technology, School of Life Sciences-Mysuru, JSS Academy of Higher Education & Research (JSS AHER), Mysuru, Karnataka, India; 3 JSS Academy of Higher Education & Research (JSS AHER), Mysuru, Karnataka, India; 4 Department of Physiology, JSS Medical College, JSS Academy of Higher Education & Research (JSS AHER), Mysuru, Karnataka, India; 5 Leader, Special Interest Group in Cancer Biology and Cancer Stem Cells (SIG-CBCSC), JSS Medical College, JSS Academy of Higher Education & Research (JSS AHER), Mysuru, Karnataka, India; Columbia University, UNITED STATES OF AMERICA

## Abstract

Prior studies from our laboratory have shown that cancer cells exposed to vitamin D3 exhibited reduced proliferation in breast cancer cells due to the upregulation of p53 and downregulation of cyclin-D1. Furthermore, in mice, our group has demonstrated that administration of 125 µg/kg of vitamin D3 retarded the growth of EAC tumors. But, it is unknown whether vitamin D3 exerts similar anti-cancer effects against cell lines representing carcinomas of the liver, colon and rectum, cervix, and brain. It is also unknown whether administration of vitamin D3 by i.p alone is sufficient for better tumor inhibition or combined administration consisting of i.p. and intratumoral (i.t.) routes is required. Furthermore, the ability of vitamin D3 in reducing the tumor growth in normal and diabetic mice has not been studied to date. Addressing these lacunae, we have prepared the dose and time response curves for vitamin D3 against different cancer cells and assessed the impact on pathways regulating cell survival and cell proliferation. A dose-dependent decrease in the (a) number of proliferating cells; (b) viability and (c) an increase in apoptosis (as evidenced by increased cleaved caspase-3) were observed with vitamin D treatment. Mechanistically, low dose vitamin D3 (15.62µM and 31.25µM) increased the expression of p53 and p21 at 24h and 48h of treatment. Interestingly, we could only observe minor changes in the expression of Bax, Bcl2 and Survivin proteins with vitamin D3 treatment. In mice, i.p. and i.t. combination reduced the tumor growth much more effectively compared to i.p. alone. Our data also showed that vitamin D3 could retard tumors developing in normal and hyperglycaemic mice. In summary, vitamin D3 is a potent anti-cancer agent, hence, is recommend for further development to treat cancers.

## Introduction

Despite the implementation of various screening programs and the development of several preventive and treatment strategies, the incidence and mortality due to various cancers are increasing at an alarming rate [1]. According to the data published by “Our World in Data: Burden of Disease 2019”, cancer is the second leading cause of death globally [2]. According to the recent statistics of Global Cancer Observatory (GLOBOCAN) 2022, about 19.9 million new cancer cases were diagnosed, and 9.7 million deaths were reported due to different cancers [3]. Among different cancers, the carcinomas of the lung, breast, and colorectal are in the top 3 places in terms of incidence; and the cancers of the lung, colorectal, and liver are in the top 3 positions in terms of mortality [3]. Although the current treatment options for cancer therapy (including surgery, radiation therapy, chemotherapy, targeted therapy, immune therapy, and hormonal therapy) are effective in the initial stages, these treatment methods often remain unsuccessful if the cancer is advanced to malignant and metastatic stages [4]. Consequently, the most promising strategy for cancer therapy is the combination of anti-cancer medications with natural supplements or chemicals that have minimal side effects and better pharmacological profiles [5]. It has been demonstrated that several natural and essential nutritional substances can effectively eliminate cancerous cells by reducing resistance while enhancing the immune defense network [6]. Therefore, the search for such nutritional supplements continues, and recently, the focus has shifted toward the utilization of fat-soluble vitamins (in particular vitamin D3 and vitamin E) for the treatment of cancers [7].

Vitamin D3 is a fat-soluble secosteroid prohormone (precursor to the potent steroid hormone calcitriol (also known as 1,25-dihydroxy vitamin D3)), which mediates numerous actions in humans [8]. For many years, the function of vitamin D3 was limited to calcium and phosphorus homeostasis, i.e., bone mineralization [9,10] but, during the past 20 years (after the discovery of vitamin D receptors (VDR) in various cell types), it became evident that vitamin D3 has various extra-skeletal effects including the regulation of several physiological processes such as cell proliferation, differentiation, and immune modulation [8,9]. Vitamin D3 functions by binding to VDR, a member of the nuclear hormone receptors super-family [11]. VDR, once activated by the binding of vitamin D3, interacts with retinoid-X-receptor (RXR) and form a heterodimeric complex, which is recruited to the vitamin D response elements (VDRE) in the target genes to activate or repress their expression [12,13]. But, recent studies have also demonstrated VDR-independent actions of vitamin D3 [14].

Accumulating evidences suggest that vitamin D3 deficiency is associated with the development and progression of many chronic diseases including cancer [15–20]. While several epidemiological studies have demonstrated the association between vitamin D3 deficiency and cancer, many laboratory investigations employing a variety of cell-based and animal models have shown the ability of vitamin D3 to mitigate the development and progression of cancer [8]. Vitamin D3 is known to regulate multiple signaling pathways in cancer cells that include control of cell proliferation, differentiation, invasion, metastasis, angiogenesis, apoptosis, and inflammation [8]. In addition, recent studies have proven that vitamin D3 can promote anti-tumoral immunity by modulating the gut microbiome in certain cancers [21]. Therefore vitamin D3 has the potential to affect the development and progression of cancer [8]. Although the anticancer effects of vitamin D3 are reported in a wide range of cancers *in vitro*, more detailed mechanistic insights were obtained in the case of carcinomas of breast and prostate [8]. For instance, recently our group has reported the anticancer role of vitamin D3 in breast cancer cell lines *in vitro* and Ehrlich ascites carcinomas (EAC) *in vivo* [22]. However, it is currently unknown which among different cancer cell lines is more responsive to vitamin D3 treatment.

Vitamin D3 is usually provided in the form of supplements to deficient individuals [23]. Studies have shown that supplementation of vitamin D3 in the diet helps in reducing the incidence of cancers [8]. However, vitamin D3 is given as capsules/tablets/sprays to treat cancer patients [24]. Currently, it is unknown whether vitamin D3 could be given intra-tumorally and, if so, whether intraperitoneal (i.p.) administration along with intra-tumoral (i.t.) injections of vitamin D3 synergistically reduces solid tumors. In this study, we have tested this possibility by comparing the efficacy of vitamin D3 when given i.p. alone and i.p. along with i.t. We could demonstrate that administration of vitamin D3 in a combination of i.p., and i.t., is relatively more effective compared to i.p. alone.

Recent investigations have reported that vitamin D3 is deficient even in individuals suffering from diabetes mellitus [25]. Preclinical and epidemiological studies have shown that supplementing the diet with vitamin D3 could in part reduce the complications of diabetes [26,27]. Recent studies have also shown enhanced tumor growth in individuals suffering from hyperglycemia, i.e., Type-2 diabetes (T2D); and vitamin D3 is deficient not only in cancer patients but also in individuals suffering from T2D. Further, recent reports have also shown that tumor cells are more resistant to chemotherapeutic agents if they are grown in high-glucose/ high-fat medium [28,29]. Based on these observations and evidences from *in vitro* and *in vivo* studies, we have hypothesized that vitamin D3 might help in mitigating T2D-induced complications while reducing the growth of tumors.

In the current investigation, we have compared the efficacy of vitamin D3 for inhibiting cell lines representing carcinomas of the cervix, colon and rectum, liver, and brain. Molecularly, vitamin D3 increased the expression of p21. Cell line dependent minor changes were also observed in the expression of survivin and Bax/Bcl2 ratio. Next, we have determined the effect of administering vitamin D3 to mice having or not having hyperglycemia but bearing EAC tumors. Results of these experiments showed that vitamin D3 inhibited the growth of tumors even in hyperglycemic mice. Intraperitoneal and intratumoral administration of vitamin D3 more effectively reduced the progression of EAC solid tumors in *Swiss albino* mice. Mechanistically, vitamin D3 reduced the vascular density (CD31 staining) and number of proliferating cells (Ki67 staining) in EAC solid tumors. Intraperitoneal and intratumoral administration of vitamin D3 was found to be more effective in reducing the progression of EAC solid tumors in non-diabetic mice. Future studies can consider testing vitamin D3 in association with various anti-hyperglycemic agents to check whether this combination approach yields a synergistic effect.

## Materials and methods

### Materials

Cell lines: Hepatocellular carcinoma cell lines Hep 3B (passage #20–30) and Hep G2 (passage #30–40); Colorectal cancer cell lines HCT 116 (passage #40–50) and HT 29 (passage # 35–45), Human and rat glioblastoma cell lines U-87 MG (passage #20–30) and C6 (passage # 20–50), respectively, and Cervical cancer cell lines HeLa (passage # 80–90) and SiHa (passage # 20–30) were procured from National Center for Cell Science, Pune, Maharashtra, India. EAC cells were provided by Dr. Prabhakar B.T. Professor, Post Graduate Department of Studies and Research in Biotechnology, Molecular Biomedicine Laboratory, Sahyadri Science College Kuvempu University, Shimoga, Karnataka, India.

### Reagents for cell culture, dyes & pharmacological agents:

Dulbecco’s Modified Eagle Medium containing high glucose (4.5g/L) and sodium bicarbonate (3.7g/L) but lacking L-Glutamine and sodium pyruvate (Hybridoma Tested), and Eagle’s Minimum Essential Medium (MEM) w/ Earle’s salts, 2mM L-Glutamine, 1mM sodium pyruvate, 1.5g/L sodium bicarbonate (Hybridoma Tested) were purchased from Hi-Media Laboratories Pvt Ltd, Bengaluru, Karnataka, India. Fetal Bovine Serum (FBS), Penicillin & Streptomycin mixture, Trypsin-EDTA (0.25%), Dulbecco’s Phosphate Buffered Saline without phenol red, calcium and magnesium, Glutamax (100X), RIPA buffer were purchased from Thermo Fisher Scientific (Waltham, MA, USA), MTS (3-(4,5-dimethylthiazol-2-yl)-5-(3-carboxymethoxyphenyl)-2-(4-sulfophenyl)-2H-tetrazolium), was procured from Promega Corporation, Madison, Wisconsin, USA.

### Antibodies for Western blot:

Vitamin D receptor (VDR) (Cat# sc-13133), Cyclin D1(Cat# sc-8396), p21(Cat# sc-6246), were procured from Santa Cruz Biotechnology, Dallas USA; Bcl2 (Cat# PAA778hu01) and Beta actin (CAB340Mi22) were procured from Cloud-Clone Corp. Houston, USA; Bax (Cat# 5023s), p53(Cat# 9282), Caspase 3(Cat#9662) and Survivin (Cat# 2803s) were procured from Cell Signaling Technology, Massachusetts, USA.

### Antibodies for IHC:

Antibodies for IHC were procured from Path-n-Situ Biotechnologies, Hyderabad, Telangana, India. The antibodies procured were - Ki-67 (Cat# PM210), p53 (Cat# PM101), and CD31 (Cat# GM006).

### Experimental animals:

Swiss albino mice (Gender-Female, Age: 4–6 weeks, and Weight: 23-25g) were purchased from CPCSEA certified Vaarunya Bio-Labs Private Limited, Bengaluru, Karnataka, India.

### Kits for measuring the biochemical parameters:

Analysis of the serum (collected from mice) for various biochemical constituents was carried out by using kits from Agappe Diagnostics Ltd. Bengaluru, Karnataka, India, and E-Lab Science Biotechnology Inc. Houston, Texas, United States.

The following kits were used in this study: Vitamin D3 (Cat# E-EL-0012), Cholesterol (Cat# 51403002, HDL (Cat#51414003, Triglycerides (Cat# 51410002, Calcium (Cat# 11006001 and Inorganic phosphorous (Cat# 12006018)

## Methods

### Determination of anti-proliferative potential of vitamin D3:

Liver (Hep 3B and Hep G2), colorectal (HCT 116 and HT-29), glioblastoma (U-87 MG and C6), and cervical cancer (HeLa and SiHa) cells were seeded in a 96-well plate at a density of 1 × 10^4^ cells/well in 100 µL of complete medium and allowed to adhere for 24h. The cells were incubated in a carbon dioxide incubator maintained at 37^o^C with 5% CO_2_ and 90% relative humidity. After 24h, when the confluence reached about 60% to 70%, the cells were exposed to increasing concentrations of vitamin D3 (7.8, 15.6, 31.25, 62.5, 125, 250, and 500μM) for 24h, 48h and 72h respectively. Cisplatin (100 µM) was used as positive control. The vehicle control, i.e., DMSO, concentration was maintained at 0.5%. Cell viability was determined by using sulforhodamine B (SRB) assay according to Skehan et.al.,1990 [30]. Percentage cell death was carried out by using the following formula.

Percentage cell death = 100-(OD of Control – OD of Sample)/ (OD of Control) X 100. A minimum of two independent experiments with at least 3 replicate wells for each concentration was performed.

### Determination of cell viability using MTS assay:

EAC cells derived directly from mouse ascites were plated in a 96 well plate, and allowed to grow for 24h. After 24h, when the cells reach about 60% to 70% confluence, they were exposed to increasing concentrations of vitamin D3 (7.8, 15.6, 31.25, 62.5, 125, 250, and 500μM) for 24h and 48h. Cell viability was determined by using MTS assay. A stock of 2 mg/mL MTS was prepared in 10mL of sterile PBS. Then, MTS and PMS (0.92 mg/mL in PBS) were mixed in 20:1 ratio, and 20 μL of this mixture was directly added to cells growing in 100 µL complete medium. The plates were incubated for 2h in a CO_2_ incubator at 37°C, and were gently tapped to ensure the solubility of dye in media. The absorbance was measured at 570nm in a multimode plate reader. The percentage viability was calculated using the equation below.


% Viability = [(OD of Test/ OD of Control) x 100]

Percentage cell death = 100-(OD of Control – OD of Sample)/ (OD of Control) X 100. All the experiments were repeated atleast two times with 3 replicate wells for each concentration.

### Determination of IC50 of vitamin D3:

The efficacy of vitamin D3 was determined by performing a dose-response study as detailed before. The percentage inhibition compared to control vehicle-treated cells was determined and the data was analyzed using GraphPad Prism Version 8.0. The IC50 values were calculated from 3 independent experiments to check the reproducibility. Results were represented as IC50 ± SEM.

### Determination of the impact of vitamin D3 on cell number:

To quantify the variations in the cell number upon treatment with vitamin D3, C6 cells that showed good response to vitamin D3 were seeded at a density of 1X10^4^ cells/well in 100 µL complete medium and allowed to proliferate for 12h, 24h, 48h and 72h. At each time point, cells from three wells (independently) were trypsinized and counted by using Neubauer chamber. The cell number was plated against time point to gain insights in to the impact of vitamin D3. According to this assay, decrease in cell number compared to the baseline (0h) shows cell death induction, whereas a minimal- (~10% or low) or no increase in cell number shows a cytostatic effect.

### Assessment of vitamin D3-induced cell death by dual staining:

To determine the cell death-related changes induced by vitamin D3, a dual staining procedure using AO and EtBr reagents was performed as detailed in S. Shailasree et al., 2015 [31]. Liver, colorectal, glioblastoma, and cervical cancer cells (0.5 X 10^6^ cells/2mL Media/Well) were grown in a 6-well plate for 30h, and subsequently treated with vitamin D3 for 24h and 48h. DMSO (0.5%) and cisplatin (100μM) were included as vehicle and positive control, respectively. Control and treated cells were trypsinized by the addition of ~150 μL of 0.25% Trypsin-EDTA and mixed gently to obtain a single-cell suspension. Trypsin was neutralized by the addition of complete media (~150 μL), and the cell suspension was centrifuged at 900 X g for 5 minutes at 4^o^C. The cell pellet was resuspended in 20 μL PBS. Later the cell suspension was mixed with AO (working concentration of 10 μg/mL in PBS) and 10 μL EtBr (working concentration of 10 μg/mL in PBS) staining solution, followed by incubation in the dark for 10 minutes at room temperature. The stained cells were assessed using a fluorescence microscope (BX53, Olympus Corporation Shinjuku, Tokyo, Japan) operating with TRITC (Tetramethylrhodamine) and FITC (Fluorescein Isothiocyanate) filters. All the images were captured using green and red channels and merged to visualize a combined image showing green (Live) and red (Dead). At least 4 different fields were considered for quantification of live and dead cells. The percentage of cells undergoing death over total cells/field was considered for the quantification of cell death at 24h and 48h and the data were represented as a bar graph.

### Cell cycle analysis:

Cell cycle analysis was carried out using the NucleoCounter® (NucleoCounter® NC-3000™, ChemoMetec, Allerod, Denmark). Approximately, 1x10^6^ HCT 116 and Hep 3B cells from control untreated and vitamin D3 treated (for 24h and 48h, concentrations are similar to the one used for AO/EtBr staining assay) groups were collected and centrifuged for 5 min at 500 X *g* at room temperature (5430R, Eppendorf, Hamburg, Germany). The pellet was washed once with PBS and resuspended in 250 μL of Solution 10 (Lysis Buffer Product No. 910–3010) supplemented with 10 µg/mL DAPI (4’,6-diamidino-2-phenylindole, Solution 12 Product No. 910–3012). This mixture was incubated at 37°C for 5 minutes. Upon completion of the incubation, 250 μL of Solution 11 (Stabilization Buffer Product No. 910–3011) was added. Stained cells (10 μL) were loaded into NC-Slide A8 (Product No. 942–0003) and analyzed using the Two-step Cell Cycle Assay program of NC-3000. The data were analyzed based on the number and intensity of DAPI-stained cells. Histograms were captured, and the percentage of cells in each stage of the cell cycle determined. Results were analysed by comparing the vitamin D3 treated cells with untreated control cells.

### Western blotting:

Western blotting was carried out to determine the changes in the expression of survival, apoptosis and cell cycle regulator proteins. Experimentally, whole cell lysates of the hepatocellular- and colorectal carcinoma cell lines (Hep 3B and HCT 116, respectively) were treated with different concentrations of vitamin D3, and were collected by the addition of RIPA (Radio immunoprecipitation assay) buffer. Samples were vortexed 5–6 times for 1.0 minute each at an interval of 5 minutes. The lysates were centrifuged at 9391 X *g* for 10 mins at 4°C and the supernatant collected. Total protein content was determined by BCA method. Fifty micrograms of total protein was separated using 12% SDS-PAGE as detailed by Gowda et. al [32]. The blots were probed with antibodies of interest and detected by the addition of appropriate secondary antibody tagged with horse raddish peroxidase (Table 1). The bands were then visualized using ECL followed by capturing the image in a Chemidoc system (Alliance Q9 UVITec Cambridge UK). The bands were quantified using Image J software and the expression level was normalized by dividing the area of target protein with that of loading control.

**Table 1:** Primary and Secondary antibodies used.

Particulars of Primary Antibody		Particulars of Secondary Antibody	
**Antibody**	**Dilution**	**Antibody**	**Dilution**
VDR	1:500	Anti Mouse	1:5000
Cyclin D1	1:500		
Beta Actin	1:3000		
p53	1:1000	Anti Rabbit	1:5000
Bax	1:2000		
Bcl2	1:2000		
Survivin	1:1000		
p21	1:1000		
Caspase-3	1:1000		

### Evaluation of the anti-tumor activity of vitamin D3 in normal and diabetic Swiss albino mice:

The experimental protocol for tumor kinetics studies was approved by the Committee for the Purpose of Control and Supervision of Experiments on Animals (CPCSEA), Govt of India recognized Institutional Animal Ethical Committee (JSS AHER/CPT/IAEC/130/2022 and JSS AHER/CPT/IAEC/125/2022) of JSS Academy of Higher Education & Research (JSS AHER), Mysore, Karnataka, India. The committee consists of experts in the field of animal sciences as well as in the biological sciences. The mice were housed in the Centre for Experimental Pharmacology and Toxicology (CEPT), which is a Govt. of India licensed centre located in JSS Academy of Higher Education & Research, Mysore, Karnataka, India (The licence number is 261/PO/ReBi/S/2000/CCSEA). A total of 78 Swiss albino mice (Female, 4–8 weeks old; body weight ranging from 22.0g to 28.0g) were used in this study. The study was carried out for a total of 47 days, during which the animal health and behaviour were monitored on a daily basis by the animal-care taker as well as by the person in-charge of the facility. No mice were found dead, except that one mouse from the Cisplatin 2.5 mg/Kg group found dead on day 45, (just before the end of the study). Mice were euthanized if any symptoms of necrosis (in the tumor) were noticed and or the size of the tumor attained a volume between 2000–2500mm^3^ in the untreated control mice (Note: All the mice treated with vitamin D3 and cisplatin had much smaller tumor volumes compared to control mice). Euthanization was carried out soon after the completion of the treatment by CO_2_ asphyxiation followed by cervical dislocation as recommended by the CPCSEA guidelines. Euthanization of mice was performed in a location, which is separate from the room where the experimental mice were housed. Care was executed to minimize the anxiety, pain and distress to the mice during euthanization procedure.

### Anti-tumor efficacy of vitamin D3 in normal mice:

The EAC solid tumor study in normal mice was carried out according to Om-Ali Elkhawaga et al., 2019 and Prashanth et al., 2023 [22,33]. Female Swiss albino mice were used in this study. The mice were acclimatized for 5 days and were grouped into 5 groups as mentioned below

1. Normal Control (NC) n = 3
2. Tumor Control (TC) n = 19
3. Vehicle Control (VC) n = 10
4. Vitamin D3 125 µg/Kg-(Vit D3 125 µg/Kg) n = 10 (6 mice for i.p. + i.t. and 4 mice for i.p.)
5. Positive Control-Cisplatin 2.5 mg/Kg (PC) n = 14

Since our pilot studies showed the death of mice in the tumor control (due to tumor burden) and in the cisplatin (due to poor tolerance of some of the mice) groups, a greater number of mice was included in the study. Beginning from the 6^th^ day, 0.1M citrate buffer (used as a vehicle to prepare streptozotocin) was administered to the mice every alternate day till the 12^th^ day. On the 14^th^ day 2X10^6^ viable EAC cells/mouse were injected intramuscularly (viability determined by Trypan blue exclusion method before injecting in to mice [34]) and the tumors were allowed to develop for the next 2 weeks (14–28 days). From the 28^th^ day, vitamin D3 (125 µg/Kg) was administered to 6 mice intraperitoneally (i.p.) and intratumorally (i.t.), while the remaining 4 mice were administered 10 doses of vitamin D3 intraperitoneally.

During the treatment period, tumor volumes were measured using Vernier caliper once every alternate day. The length and width of the tumor were measured and the volume was calculated using the (W^2^ × L)/2 formula [35]. Mice were sacrificed after the completion of treatment. Blood, tumor, and vital organs were harvested for further processing. The collected tumors were rinsed with PBS, weighed, and fixed in formalin for further analysis by H&E and IHC.

### Anti-tumor efficacy of vitamin D3 in diabetic mice:

The study was carried out as detailed by Kozokoshizuka et al., 1999 [36]. Female Swiss albino mice (4–6 weeks old; 23–25g) were arranged randomly into different groups as shown below.

1. Normal Control n = 3
2. Streptozotocin-induced diabetic mice with EAC solid tumor (STZ + TC representing Tumor Control) n = 7
3. Vehicle control (VC-0.5% DMSO) n = 4
4. Streptozotocin-induced Diabetic mice with EAC solid tumors – Treated with 125 µg/Kg of vitamin D3 (Referred as STZ + TC + Vit D3 125 µg/Kg) n = 4
5. Streptozotocin-induced Diabetic mice with EAC solid tumors – Treated with 2.5 mg/Kg of Cisplatin (Referred to as STZ + TC + PC) n = 4

Mice were acclimatized for 5 days. Stock streptozotocin (SR Life Sciences, Bangalore, Karnataka, India) was prepared by dissolving in cold 0.1M citrate buffer, pH 4.5, and injected intraperitoneally from the day 6 at a dosage of 30 mg/Kg and 40 mg/Kg every alternate day till day 12. The fasting blood glucose (FBG) level was measured every other day to determine the induction of diabetes. The blood was collected by tail prick method (from the tail vein) and glucose concentration was measured using a glucometer (Dr. Morpen, New Delhi, India). After confirming the induction of diabetes (FBS value 198 ± 9.32 mg/dL), EAC cells were injected on the 14^th^ day (2X10^6^ viable cells/mouse) intramuscularly to the left thigh [22]. EAC cells were allowed to develop into solid tumors for 14 days. Vitamin D3 (125 µg/Kg) and Cisplatin (Positive control PC) 2.5 mg/Kg were administered from day 28 till day 46. A total of 10 doses (intraperitoneally) were administered every alternate day. During the treatment period, the length and width of the tumor were measured and the volume was calculated using the (W^2^ × L)/2 formula [35]. The collected tumors were rinsed thoroughly in PBS, weighed, and photographed.

### Analysis of blood collected from experiment in normal mice

In order to determine the impact of treatment on various blood parameters, serum was separated from the whole blood collected from the mice (as listed below) and assays performed as per the instructions given by the supplier.

1. Normal control (NC) n = 3
2. Tumor Control (TC) n = 5
3. Vehicle Control (VC) n = 4
4. Vitamin D3 125 µg/Kg (Vit D3) n = 3
5. Cisplatin 2.5 mg/Kg (Positive control PC) n = 3

Blood was drawn from mice through the tail vein, and was used to separate the serum. The content of vitamin D3, random blood glucose (RBG), Triglycerides (TGs), high-density lipoproteins (HDL), total cholesterol (TC), Calcium, and Phosphorous levels were measured using kits purchased from Agappe Diagnostics Limited (Pattimattom, Ernakulam, Kerala, India) and Elabscience Biotechnology Inc (Houston, Texas, USA). Assays were carried out as per the instructions in the respective kit inserts. Random blood glucose was estimated at the end of the study to determine the effect of treatment.

### Immunohistochemical (IHC) analysis of tumors:

Upon completion of the study, the tumor and vital organs were collected and fixed immediately in freshly prepared 10% formalin solution as detailed before [37]. The tissues were fixed for about 24 hours before taking them for further processing. Dehydration of the tissue was carried out by exposing to different concentrations of ethanol. Further, the tissues were subjected to clearing using xylene. Tissues were exposed to xylene for a maximum time of 45 minutes. Wax infiltration was carried out by exposing the tissues to liquid wax at 60°C and further cooled to 20°C. Tissue blocks were prepared by placing the tissue carefully into moulds filled with molten wax and a cassette was placed over it, and allowed to cool. The tissue blocks were retrieved after the wax had set, and sections of 3.0 to 4.0µm thick were prepared using a microtome (RM2255 Leica Biosystems, Nussloch Germany).

Tissue sections of 4.0µm thickness were taken on poly L-lysine coated slides (S21.2113.A Leica BOND Plus Slides, Leica Biosystems, Nussloch Germany) and incubated overnight at 65°C. Sections were de-paraffinized for 15 minutes using xylene and dehydrated with absolute ethanol for 4 minutes. Antigen retrieval was carried out by subjecting the slides to a buffer containing Tris-EDTA at 95°C for 10 minutes. Sections were cooled for 10–15 mins and washed with Tris-buffer pH 7.2. H_2_O_2_ (10%) was used to block the endogenous peroxidase activity. The slides were then incubated with the primary antibody (Ki-67, p53 and CD31 procured from Path-n-Situ Biotechnologies used at a concentration of 1:100) at room temperature for 60 minutes. Detection was performed by using 3,3’-diaminobenzidine (DAB) reagent for 5–7 minutes, which resulted in a brown-colored precipitate. These slides were counterstained with hematoxylin and mounted with D.P.X mountant (Thermo Fischer, Waltham, Massachusetts, United States).

### Statistical analyses:

Cytotoxicity assays were carried out atleast 2 times with 3 replicate measurements in each experiment. The bars represent the average of three independent experiments with the significance (P) determined by One-Way ANOVA at each time point. The data represents the mean of at least 3 different fields. Differences between control and vitamin D3 treated groups were calculated and the level of significance was assessed by GraphPad Prism. One-way ANOVA was applied for measuring the significance among the different groups.

## Results

### Vitamin D3 reduced the viability of cell lines representing carcinomas of the liver, colon and rectum, cervix, and brain:

**Fig 1:**
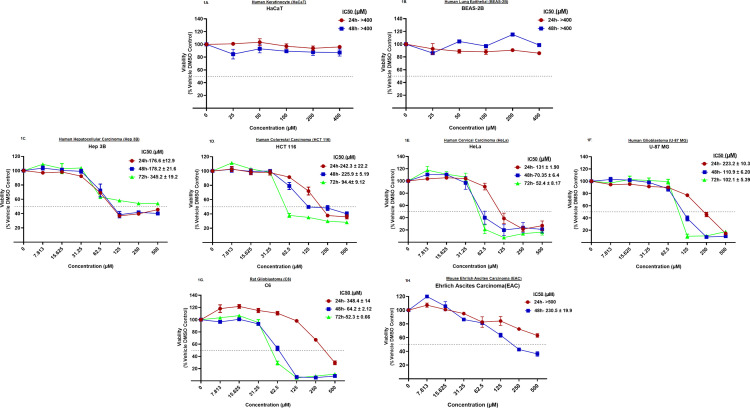
Treatment with vitamin D3 reduced the viability of cancer cell lines: *1A & 1B.* Human keratinocyte cell line HaCaT and human lung epithelial cell line BEAS-2B did not respond to vitamin D3 treatment. Very minimal cytotoxic effect (<10% cell death) was observed at the highest concentration of vitamin D3 tested (i.e., 400µM). 1C. Liver cancer cell line Hep 3B exposed to increasing concentrations of vitamin D3 showed a dose-dependent increase in cytotoxic effect beginning from 31.25µM; 1D. Exposure of colorectal carcinoma cell line HCT 116 to increasing concentrations of vitamin D3 showed a dose- and time dependent increase in cytotoxic effect; 1E. Cervical cancer cell line HeLa exhibited dose- and time-dependent response pattern to vitamin D3 treatment; 1F & 1G. Human and rat glioblastoma cell lines U-87 MG and C6, respectively; which were exposed to vitamin D3 demonstrated a statistically significant reduction in viable cells with increasing time and treatment concentration. 1H. Mouse EAC cells showed a decrease in cell viability starting from 250 µM at 24h of treatment. Better cytotoxic effect of vitamin D3 was observed at 48h of treatment starting from 125 µM concentration. Cytotoxicity assays were carried out atleast 2 times with 3 replicate measurements in each experiment. The bars represent the average of three independent experiments with the significance (P) determined by One-Way ANOVA at each time point. *p < 0.05, **p < 0.01, ***p < 0.001, ****p < 0.0001.

Several studies, including the one from our group, have shown the anticancer activity of vitamin D3 (Cholecalciferol). But, to date, no study has compared the efficacy of vitamin D3 against cell lines representing carcinomas of the liver, colon and rectum, cervix, and brain. This information not only helps to identify the cell lines that better respond to the vitamin D3 treatment but also helps in correctly assessing whether vitamin D3 can be considered for treating a specific cancer. To determine the efficacy of vitamin D3 against these cell lines, the exponentially growing cells were treated with increasing concentrations (7.8µM to 500µM) of vitamin D3 for 24h, 48h, and 72h and the viability was determined as detailed in the methods section. The doses were selected based on our prior publication data [22]. Analysis of the data showed a dose-dependent decrease in the viability (beginning from 31.25µM till the concentration reaches 125µM) across the chosen cancer cell lines (Fig 1).

Vitamin D3 did not show any cytotoxic effect against normal cell lines representing the human keratinocytes, i.e., HaCaT (Fig 1A) and human lung epithelium, i.e., BEAS-2B (Fig 1B). Hepatocellular carcinoma cell lines Hep 3B (Fig 1C) and Hep G2 (Supporting data S1a Fig) showed a dose-dependent decrease in viability with increasing treatment concentration. The IC50 values for these cell lines ranged from 170µM to 500µM at 24h of exposure to vitamin D3. Cell lines representing colorectal carcinomas viz., HCT 116 (Fig 1D) and HT-29 (Supporting data S1b Fig) showed a statistically significant decrease in viability at 24h and 48h. IC50 values for HCT 116 and HT-29 ranged from 130µM to 250µM upon 24h and 48h of treatment with vitamin D3. Cervical cancer cell line HeLa (Fig 1E) and SiHa (Supporting Data S1c Fig) also showed a dose-dependent response at 24h and the effect increased with extended treatment time viz., 48h and 72h. Vitamin D3 exhibited a dose and time-dependent decrease in the viability of human and rat glioblastoma cell lines U-87 MG and C6, respectively (Fig 1F, G). Interestingly U-87 MG and C6 cell lines (despite differences in their origin of species, i.e., human and rat) showed a very similar effect with IC50 values ranging from 220µM to 350µM at 24h. Mouse derived EAC cells, upon treatment with vitamin D3, showed reduced viability with an IC50 of 230.5µM after 48h of treatment (Fig 1H).

Further, in order to determine the processes (inhibition of cell number or induction of cell death) that are responsible for vitamin D3 treatment-induced cytotoxicity, the C6 cells were plated and treated with different concentrations of vitamin D3 for 12h, 24h, 48h and 72h. Number of cells at each time point was counted and compared with untreated control (at base line, i.e., 0h and at the corresponding time points). Vitamin D3 treatment reduced the cell number with increasing treatment time and concentrations, suggesting the percentage inhibition is probably due to cell proliferation arrest followed by the induction of cell death (Supporting Data S2 Fig). In summary, vitamin D3 reduced the viability of cancer cells with varied IC50 values. Analyses of the IC50 values showed a time-dependent decrease in the concentration of vitamin D3 with the increase in the exposure time in majority of cancer cell lines. However, the IC50 value of hepatocellular carcinoma cell line Hep 3B and colorectal carcinoma cell line HT-29 has increased with increasing treatment time.

### Treatment of cancer cells with vitamin D3 induced apoptotic cell death:

**Fig 2:**
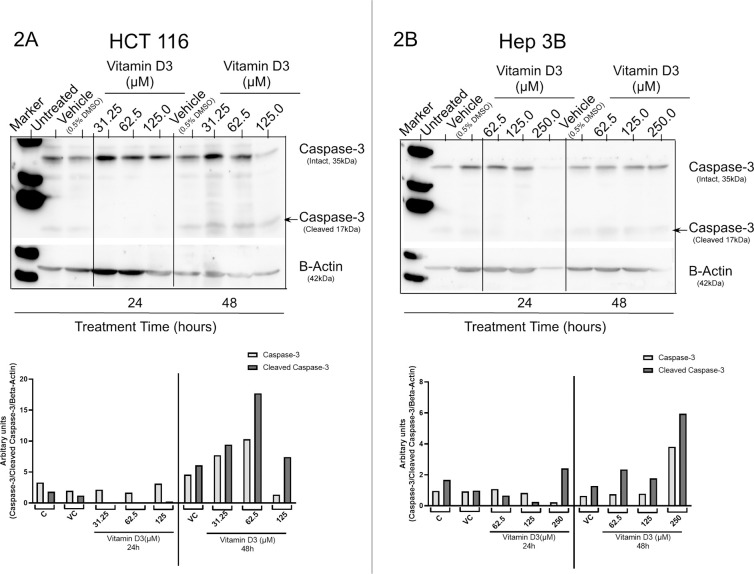
Expression of Caspase-3 in HCT 116 and Hep 3B: *2A. and 2B.* Caspase-3 expression upon treatment with Vitamin D3 in HCT 116 and Hep 3B cell lines.2A. HCT 116 showed a dose dependent increase in the 17kDa cleaved caspase fragment at 48h of vitamin D3 treatment. 2B. Hep 3B also showed a similar expression pattern with increase in cleaved caspase 3 expression when compared to the intact caspases at 48h of treatment. Both the cell lines however did not show any significant changes in the cleaved caspase 3 expression at 24h of treatment.

Since vitamin D3 reduced the viability of different cancer cell lines, next, we have determined the processes that were responsible for viability reduction. Apoptosis, a programmed cell death, is one of the primary mechanisms triggered by chemotherapeutic drugs to reduce the viability of cancer cells [38]. To determine whether treatment of cancer cells (HCT-116 and Hep3B) with vitamin D3 has induced cell death by promoting apoptosis, we have measured the expression of intact and cleaved caspase-3 by western blotting (Fig 2A, B). Analysis of the data showed an increase in the expression of 17kDa cleaved caspase fragment with a concomitant decrease in the expression of 35kDa intact caspase at 48h of treatment (Fig 2A, B). Interestingly no cleaved caspase was observed when both the cell lines were exposed to vitamin D3 for 24h (Fig 2A, B).

In addition to caspase expression, we have also stained the control untreated and experimental vitamin D3 exposed cells with acridine orange and ethidium bromide dual stain (AO/EtBr), and observed the stained cells under fluorescence microscope [39]. The dye acridine orange (AO) is permeable through cell membranes and stains both live and dead cells, but, ethidium bromide (EtBr) enters the cells only if the integrity of the cell membrane is lost [40]. According to this protocol, the live cells appear green without having any green dots, whereas the late apoptotic cells appear orange in color and exhibit shrunken morphology [41]. In contrast to late apoptotic cells, the necrotic cells, which are also in orange color, appear bulk in size with necrotic blubs. Considering these features as indicators of live/apoptotic/necrotic cells, we have counted at least 4 different fields in each stained slide and represented the results as percentage of cells undergoing death over total cells. Based on the dose and time response studies, three concentrations (below, above and at IC50 of vitamin D3) were selected for each cell line. In some cell lines, higher doses of vitamin D3 showed 100% cell death (in particular when the treatment time is prolonged) hence the lower doses were used to treat the cells [42,43].

**Fig 3:**
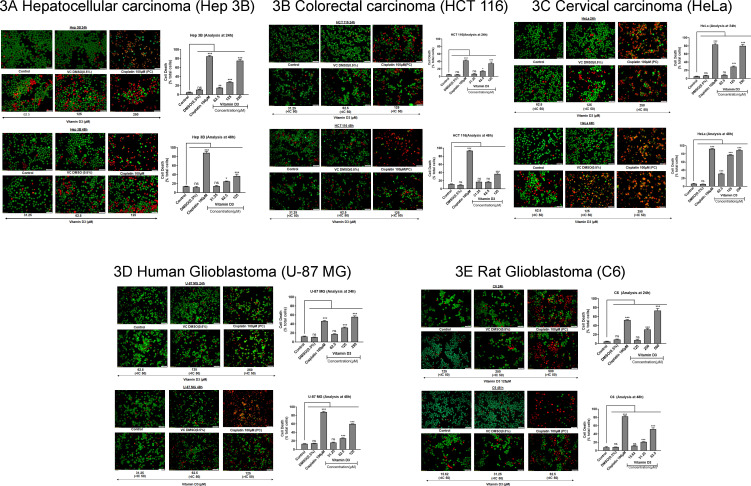
AO/EtBr Staining of Cancer cell lines: *To check whether vitamin D3 promoted cell death in cancer cells, a dual staining protocol was implemented as detailed in the methods section and the representative photomicrographs and corresponding quantification data presented.3A.* Hep 3B cells treated with 250µM vitamin D3 showed cell death similar to that of 100µM cisplatin at 24h. Prolonged exposure to vitamin D3 also produced a dose-response with maximal effect (~40% cell death) observed at 125µM; 3B. CRC cell line HCT 116 also exhibited maximal cell death at 125 µM vitamin D3, but fewer dead cells were observed at lower doses at 24h; 3C. Cervical cancer cell line HeLa showed maximal cell death at 250 µM vitamin D3. At 48h of treatment, even 62.5 µM vitamin D3 showed around 40% cell death while the higher doses showed a maximum of ~90% cell death; 3D. Human glioblastoma cell line U-87 MG showed maximal cell death at 250µM vitamin D3 and lesser dead cells at lower doses at 24h; 3E. Rat glioblastoma cell line C6 exhibited maximal cell death at 500 µM vitamin D3. The data represents the mean of at least 3 different fields. Differences between control and vitamin D3 treated groups were calculated and the level of significance was assessed by GraphPad Prism. One-way ANOVA was applied. *p < 0.05, **p < 0.01, ***p < 0.001, ****p < 0.0001.

Hepatocellular carcinoma cell lines Hep 3B (Fig 3A) and Hep G2 (Supporting Data S3a Fig) showed ~70−80% cell death at 250µM upon 24h of treatment. At 48h of treatment, even the lower dose of vitamin D3, i.e., 31.25µM showed ~20% cell death. The colorectal cancer cell line HCT116 exhibited a significant increase in cell death with increasing vitamin D3 concentrations up to 125µM at 24h (Fig 3B). But, further increase in the treatment time to 48h did not increase the cell death much. Another colorectal carcinoma cell line HT-29 exhibited ~80% cell death when treated with 250µM of vitamin D3 at 24h. Continuing the exposure to 48h showed a better effect as represented in Supporting data S3b Fig. Cervical cancer cell lines HeLa (Fig 3C) and SiHa (Supporting Data S3c Fig) showed a dose- and time-dependent response to vitamin D3 exposure as depicted in Fig 3C and Supporting data S3c Fig. Vitamin D3 (250µM) showed up to 90% cell death at 24h treatment. The lower doses showed a better effect in SiHa upon 48h treatment when compared to the HeLa cell line.

In case of human glioblastoma cell line U-87 MG, treatment with 250µM vitamin D3 showed ~60% cell death. Prolonged exposure up to 48h yielded a similar percentage of cell death even at 125 µM vitamin D3 concentration (Fig 3D). Rat glioblastoma cell line C6 also showed a dose-dependent increase in the percentage of apoptotic cells when treated with 125µM, 250µM, and 500µM vitamin D3 at 24h of exposure. Increasing the treatment time to 48h yielded a significant cell death even at lower doses of vitamin D3 (15.62µM, 31.25µM, and 62.5µM) (Fig 3E).

### Treatment of cancer cells with vitamin D3 increased sub G0/G1 population:

**Fig 4:**
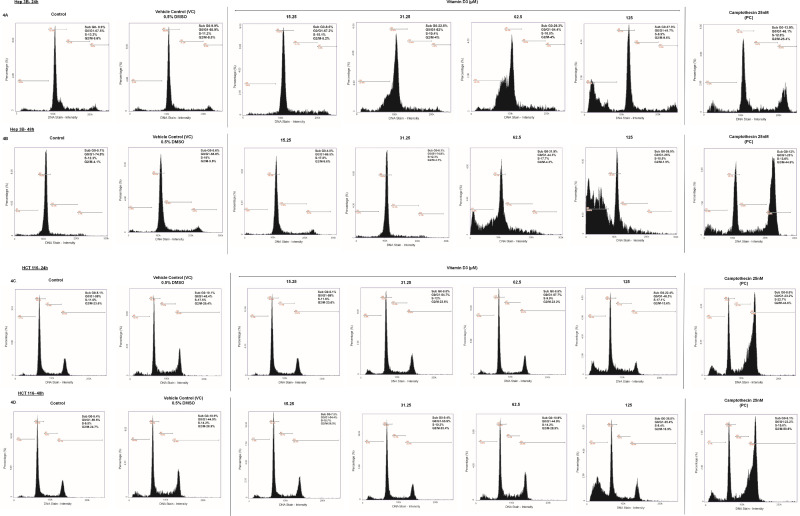
Treatment with vitamin D3 increased percentage SubG0/G1 population in Hep 3B and HCT 116 cell lines. Hep 3B and HCT 116 cells were treated with increasing concentrations of Vitamin D3 for 24h and 48h. 4A & 4B. Treatment of Hep 3B cells with vitamin D3 showed an increase in the sub G0 phase population at 24h and 48h of treatment. Vitamin D3 (125µM) showed a better effect in inducing apoptosis when compared to the positive control Camptothecin (25nM). Prolonged exposure of Hep 3B cells to vitamin D3 (for 48h) showed an increase in the sub G0 across different concentrations.4C & 4D. HCT 116 cells treated with vitamin D3 for 24h and 48h showed an increase in the sub G0 phase at 125 µM of vitamin D3, concentration.

Based on the dose and time response studies, four concentrations of vitamin D3 (15.62µM, 31.25µM, 62.5µM and 125µM) were selected for each cell line to measure the impact on cell cycle progression stages. Hep3B and HCT116 cell lines were treated with increasing concentrations of vitamin D3 for 24h and 48h as detailed in methods section. Hep 3B (Fig 4A, B and Supporting data S4a,b Fig) at 24h and 48h showed an increase in sub G0 phase population indicating cell death with increasing concentrations of Vitamin D3 treatment (Fig 4B and Supporting data S4b Fig). HCT 116 cells treated with vitamin D3 for 24h and 48h showed a slight increase in the percentage cells in sub G0 phase only at 125µM concentration (Fig 4C, D, Supporting data S4c, d Fig).

### Vitamin D3 increased the expression of apoptotic markers

To test the impact of Vitamin D3 on apoptotic- and cell cycle markers, Hep 3B and HCT 116 cell lysates were subjected to Western blotting.

**Fig 5:**
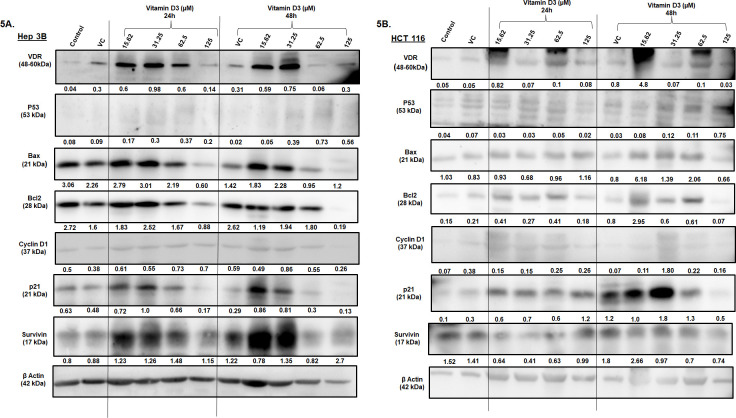
Treatment with vitamin D3 differentially altered the expression of cell death and proliferation markers in Hep 3B and HCT 116 cell lines *To determine the impact of vitamin D3 treatment on the expression of proteins involved in controlling the proliferation and apoptosis, total cell lysates before and after treatment with vitamin D3 were collected and analyzed by Western blotting.* 5A & 5B. Analysis of the blots revealed that treatment of Hep 3B cells for 24h with increasing vitamin D3 concentration increased the VDR expression in both the cell lines. Extended treatment time, i.e., 48h with vitamin D3 improved the expression status of VDR in Hep 3B. There was an increased p53 expression in Hep 3B upon treatment when compared to HCT 116 at 24h of treatment. Both the cell lines showed an increased Bax/Bcl2 ratio at 48h of treatment with the highest dose indicating the susceptibility of the cells to apoptosis. Decreased survivin protein was observed in HCT 116 at 24h of treatment when compared to Hep3B. Extended treatment of 48h showed a considerable decrease in survivin protein with increasing concentrations in HCT 116 cell line. Increased p21 expression was observed at lower doses in both the cell lines. However, this change in p21 expression did not impact the cyclin D1 expressions when compared to the untreated controls.

Whole cell lysates from Hep 3B and HCT 116 cells were extracted upon treatment with vitamin D3 for 24h and 48h. The collected cell lysates were analyzed by western blotting to measure the expression of proteins involved in the regulation of cell cycle, apoptosis and cell death. The expression of VDR has increased with low-dose (15.6 µM to 62.5 µM at 24 and 15.6 µM and 31.2 µM in case of 48h) vitamin D3 treatment in Hep-3B cell line (Fig 5A). The expression of p53 moderately increased upon treatment with vitamin D3 at 24h and 48h. The expression of Bax and Bcl2 did not change much with low-dose vitamin D3 treatment when compared to vehicle treated cells. However, a visible decrease in both Bax as well as Bcl2 was observed at the highest dose, which could be due to massive cell death induced at this concentration of vitamin D3. Comparison of Bax/Bcl2 ratio between vehicle and vitamin D3 treated cells showed a non-significant increase with increasing concentration of vitamin D3 at 24h. Survivin expression was decreased only at 62.5µM concentration 48h post treatment in Hep3B cell line. The expression of p21 showed a dose dependent increase in the expression at lower doses of vitamin D. The expression of cyclin D1 did not show any consistent trend with vitamin D3 treatment (Fig 5A).

HCT116 (Fig 5B) showed differential changes in the expression of VDR with increasing concentration of vitamin D3. Similar to Hep3B, we have observed an increase in the p53 expression at 24h and 48h of exposure to vitamin D3 (Fig 5B). Bax/Bcl2 ratio showed nonsignificant variations with vitamin D3 exposure. Unlike Hep-3B, the expression of survivin decreased with increasing vitamin D3 treatment at 48h. A dose dependent increase in p21 expression at lower concentrations was observed at 24h and 48h of treatment while a decrease in the cyclin D1 expression was observed 48h post treatment.

### Vitamin D3 retarded Ehrlich Ascites Carcinoma (EAC) solid tumors growing in normal and hyperglycemic Swiss albino mice:

Since *in vitro* studies have shown the anti-cancer effect of vitamin D3, next, we have evaluated its potential for inhibiting Ehrlich Ascites Carcinomas in mice. In general majority of cancer cell lines utilized in this study were grown in DMEM containing high glucose (4.5g/L), which mimics hyperglycaemic conditions (450 mg/dL) in humans. Therefore, in the *in vivo* model we have evaluated the impact of vitamin D3 in retarding the EAC tumors developing in healthy mice (Fasting blood glucose is between 80 mg/dL to 120 mg/dL) as well as in mice with hyperglycaemia (Fasting blood glucose is above 120 mg/dL). Low-dose streptozotocin (STZ) was used to induce diabetes as detailed in the study scheme (Supporting data S5a Fig). Fasting blood glucose (FBG) of 198 ± 9.32 mg/dL indicated the establishment of diabetes. The FBG of normal healthy mice was 115.8 ± 6.9 mg/dL (Supporting data S5b Fig).

**Fig 6:**
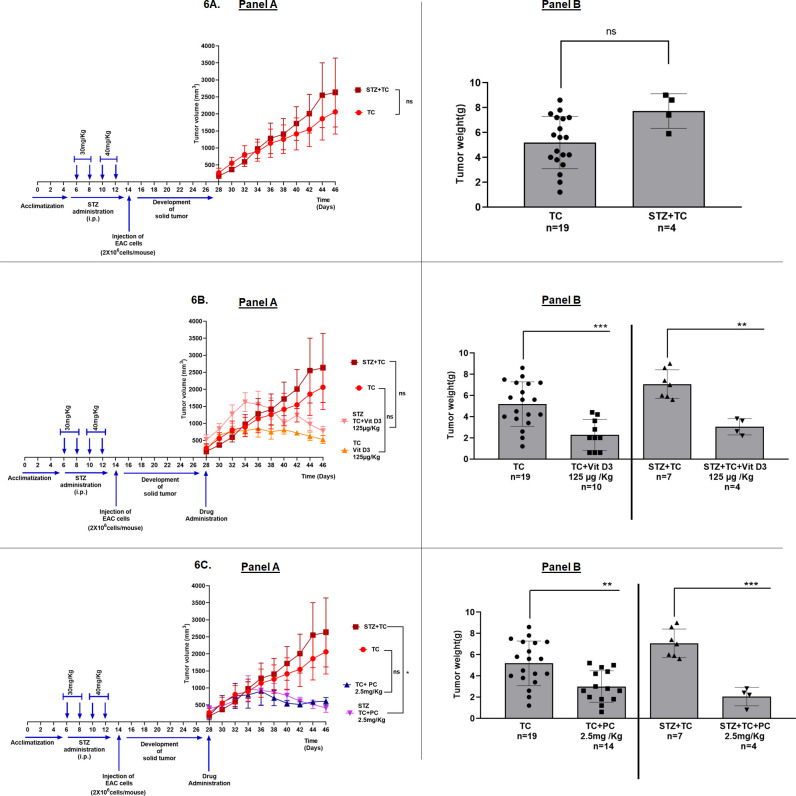
Vitamin D3 retards the growth of EAC tumors in normal and hyperglycaemic mice. 6A.Hyperglycaemia promotes the growth of EAC solid tumors in mice: To determine whether hyperglycaemia promotes EAC tumor growth, mice were first administered with STZ to induce hyperglycaemia. Next, the control normal mice and the experimental hyperglycaemic mice were injected with EAC cells to develop solid tumors. It was observed that mice with hyperglycaemia developed larger tumors compared to normal mice suggesting that elevated glucose in diabetic mice might be promoting the tumor growth.6B and 6C.Vitamin D3 and Cisplatin retarded the growth of EAC tumors in normal and hyperglycaemic mice: Since the growth of EAC tumors is much higher in hyperglycaemic mice compared to normal mice, next we have tested the ability of vitamin D3 and positive control Cisplatin for retarding the tumors in these mice categories. Both vitamin D3 (Fig 6B*) and Cisplatin (*Fig 6C*) could retard the tumors in control and hyperglycemic mice.*

During the tumor kinetics studies, we have observed that the growth of EAC tumors is much higher in diabetic mice compared to normal mice (Fig 6A Panel A and B and Supporting data S6 Fig). For instance, the tumor volume on the day of sacrifice was 2630 ± 1012.9 mm^3^ in diabetic mice, when compared to 2060.7 ± 652.38 mm^3^ in normal mice (Fig 6A Panel A).

As the growth of EAC tumors is higher in hyperglycaemic mice, our next question was to check, whether vitamin D3 is able to inhibit the tumors that are developing aggressively in diabetic mice. Experimentally, the efficacy of vitamin D3 for inhibiting EAC tumor growth was evaluated in normal and hyperglycaemic mice as shown in Supporting data S7` Fig. Intraperitoneal administration of vitamin D3 (125 µg/Kg) in hyperglycaemic- and normal mice with tumors, retarded the development of tumors compared to respective untreated controls (Fig 6B Panel A and B). For instance, the volume of tumor in normal control at the end of treatment is 2060 ± 652.3 mm^3^, vs 526.5 ± 117.6 mm^3^ in mice treated with vitamin D3 (please refer TC vs TC + vit D3 125 µg/Kg group). Similarly, the volume of EAC solid tumors in hyperglycaemic mice is 2630.57 ± 1012.99 mm^3^ (STZ + TC) vs 775.17 ± 134.5 mm^3^ of hyperglycaemic mice treated with vitamin D3 (STZ + TC + Vit D3 125 µg/Kg) (Fig 6B Panel A and B and Supporting data S7 Fig).

A significant reduction in the tumor weights was observed due to vitamin D3 treatment in normal as well as diabetic mice (Fig 6B, Panel B). Diabetic condition in the tumor control group was maintained throughout the treatment period as evidenced by elevated (compared to normal control mice) FBG (Supporting Data S5c Fig). In the treated groups FBG levels did not change significantly upon administration of cisplatin, or vitamin D3 (Supporting data S5c Fig). Cisplatin (PC 2.5 mg/Kg), showed a significant decrease in the tumor volume in diabetic mice (Please compare STZ + TC vs STZ + TC + PC), however, there was a non-significant reduction in nondiabetic mice when compared to their respective controls (Fig 6C Panel A and Supporting data S8 Fig). A significant reduction in tumor weight was also observed with Cisplatin administration in both the study models (Fig 6C, Panel B). Fasting blood glucose levels increased due to Cisplatin injection when compared to normal control and other study groups (Supporting data S5c Fig).

**Fig 7:**
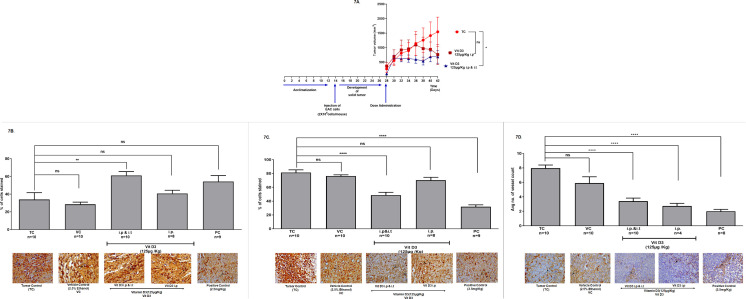
Combination of intraperitoneal and intratumoral administration of vitamin D3 is more effective in reducing the EAC tumor volume compared to just i.p. administration: 7AAdministration of vitamin D3 intraperitoneally (TC* + Vit D3 125 µg/Kg i.p.) found to be less effective compared to dual administration group.* Much better tumor reduction was achieved earlier by the administration of vitamin D3 through i.p. and i.t. combination compared to i.p. administration alone. 7B,7C and 7D.EAC tumors collected from the control and experimental mice were evaluated by immunohistochemical staining to measure p53, Ki-67, and CD31 levels (magnification 40x). Results showed that Ki-67(7C.) and p53(7B.) expression significantly changed upon vitamin D3 (125 µg/Kg) treatment through i.p,i.t route. A significant change in the Ki67 expression was observed in the cisplatin (PC 2.5 mg/Kg) treated group when compared to the control. Among the vitamin D3 groups (i.p and i.p & i.t) it was observed that there was a significant increase in the p53 expression in i.p & i.t combination compared to i.p. alone. Ki67 on the other hand showed a significant reduction in the expression in the i.p & i.t treated vitamin D3 group. 7D. Administration of cisplatin (2.5 mg/Kg) and vitamin D3 (125 µg/Kg) significantly decreased the vessel density (CD31) compared to the tumor control group.

The route of administration also determines the efficacy of pharmacological agents [44,45]. In the majority of studies, the efficacy of pharmacological agents was assessed by the intraperitoneal route of administration. But in certain cases, the drugs were also delivered by other routes such as intra-tumoral (i.t.), intravenous (i.v.), oral gavage (p.o.), and also in diet. In some conditions, a combination of routes was also selected to enhance the anti-tumor activity of drugs. Therefore, in this study, we have compared the efficacy of vitamin D3 when administered by intraperitoneal route alone and intraperitoneal administration combined with intratumoral routes (Fig 7A and Supporting Data S9 Fig). The tumor kinetics data showed that mice administered with vitamin D3 through i.p & i.t had much smaller tumors compared to the ones treated by the i.p. route (Fig 7A). The tumors collected at the end of the study were subjected to immunohistochemical analysis to check whether vitamin D3 administration had any impact on tumor cell proliferation, apoptosis and blood vessel density (Fig 7B–D).

The blood collected from control and experimental mice was centrifuged and serum separated to analyze glucose, vitamin D3, cholesterol, HDL, triglycerides, calcium, and phosphorus levels. Random blood glucose (RBG) levels in animals were checked at the end of the study to measure the effect of treatment with vitamin D3. Analysis of the serum showed no significant changes in the serum vitamin D3 levels upon treatment (Supporting data S10a Fig). Serum calcium levels also did not show any significant changes in the vitamin D3 treated groups (125 µg/Kg) when compared to the normal control (Supporting data S10b Fig). However, the serum phosphorous had significantly increased in the vitamin D3 treated groups compared to normal control (Supporting data S10c Fig). Vitamin D3 administration did not change serum random glucose levels (Supporting data S10d Fig). Analysis of serum cholesterol, HDL, and triglycerides also showed a non-significant decrease upon treatment with 125 µg/Kg vitamin D3. A noticeable increase in the serum triglyceride level was observed with 125 µg/Kg of vitamin D3 (Supporting data S10e,f,g Fig).

### Combination of intra-peritoneal (i.p.) and intra-tumoral (i.t.) administration of vitamin D3 is more effective compared to just i.p. administration in retarding EAC solid tumors:

To determine the efficacy of vitamin D3 based on the route of administration, a set of mice bearing EAC tumors were administered with vitamin D3 intra-peritoneally (i.p.), and the other set was treated with a combination of intraperitoneal and intratumoral (i.p + i.t.) injections as detailed in the animal scheme (Supporting data S9 Fig). Administration of vitamin D3 by i.p. and i.t. (125 µg/Kg each route) significantly decreased tumor volume compared to tumor control. At the end of the experiment, the volume of EAC tumors was (1544.8 ± 504.6 mm3) reduced to 683.10 ± 218.91 mm3 (56% reduction) upon treatment with vitamin D3 by i.p. and i.t. Moreover, vitamin D3 administration through i.p & i.t showed a reduction in tumor volume at an earlier time point compared to its i.p administered counterpart (Fig 7A). In summary, the combination of i.p. and i.t. is more effective compared to just i.p. administration of vitamin D3 in reducing the tumor volume.

### Histochemical analysis of EAC tumors – Vitamin D3 retarded the growth of EAC solid tumors by decreasing blood vessel density while promoting the expression of p53:

To determine the effect of treatment of EAC tumors with vitamin D3, the mice were sacrificed and tumors harvested for histological and immunohistochemical analysis. Tumors from the control, vitamin D3-treated and the Cisplatin administered groups were collected, sectioned, and stained using H&E (Supporting data S11 Fig). Further, the sections were subjected to IHC staining to measure the expression of p53, Ki-67 (a marker for proliferation), and CD31 (a marker for blood vessel density) (Fig 7B–D).

Analysis of the stained sections showed that Ki-67 and p53 expression did not significantly change upon intraperitoneal administration of vitamin D3 (125 µg/Kg). But the dual treatment group, i.e., i.p. along with i.t., showed a -significant decrease in Ki67 stained cells while exhibiting a significant increase in the expression of p53 (Fig 7B, C). Administration of Cisplatin (2.5 mg/Kg) also significantly decreased the expression of Ki67 compared to the untreated tumor control group (Fig 7C). A significant decrease in the vessel count (as evidenced by a decrease in the CD31 stained vessels) was also observed in vitamin D3 (125 µg/Kg) and cisplatin (2.5 mg/Kg) treated groups (Fig 7D).

## Discussion

Vitamin D3 in its active form, i.e., 1,25 (OH)2D3 (calcitriol) has been reported to exhibit anti-proliferative activity against cancer cells [46]. Studies have shown that even the administration of calcidiol (vitamin D3) can inhibit the viability of cancer cells *in vitro* and retard tumors in mice [22]. Supporting these *in vitro* and *in vivo* studies several epidemiological studies have shown an inverse correlation between serum vitamin D3 and the incidence and mortality of cancers [47,48]. Likewise, several investigations have also established a strong connection between vitamin D3 and the inhibition of cancer progression [49]. For example, vitamin D3 inhibits cancer cell proliferation in lung, colorectal, and breast cancer cell lines [50]. Additionally, it was also reported that individuals with lower levels of vitamin D3 are at a higher risk of developing cancers compared to those with higher vitamin D3 levels indicating a potential link to vitamin D3 deficiency [51].

In the present study, we report that vitamin D3 inhibits the proliferation of liver, colorectal, brain, and cervical cancer cells in a dose- and time-dependent manner. The colorectal cancer cell lines HCT-116 and HT-29 were found to be more sensitive to vitamin D3 treatment compared to liver cancer cells Hep3B and HepG2, glioma cells U-87 MG and C6, and cervical cancer cells HeLa and SiHa. Similar to our studies, Jisha Elsa et al., have reported that vitamin D3 is more effective against HCT-116 (IC50–15 µM) [50]. In another study, Shruti et al., have reported that vitamin D3 exhibited an IC50 of 50µM on HCT-116 [51]. J. M. Wierzbicka et al. reported that active vitamin D3 and its analogs were able to inhibit the proliferation of HT-29 cells [52].

In case of liver cancer cell lines HepG2 and Hep3B, the vitamin D3 is more effective against HepG2. This observation is consistent with an earlier report by Antonio Caputo et al., wherein vitamin D3 induced an anti-proliferative effect by promoting cell cycle arrest at the G0/G1 phase in HepG2 cell line [53]. This enhanced sensitivity of HepG2 to vitamin D3 is ascribed to very high VDR expression in this cell line [53].

Several studies have reported the ability of vitamin D3 to induce the expression of VDR [54]. Vitamin D3-induced VDR expression was reported in glioma cells U251, U-87 MG, and T98G [55]. Similarly, another study by Baudet C et al., found that calcitriol could induce VDR expression and increase apoptotic death in C6 glioma cells [56]. This is also in agreement with the effect on cervical cancer cells SiHa and HeLa wherein enhanced VDR expression and activity were observed along with the CYP27A1 and CYP27B1 gene upregulation up on treatment with calcitriol [57–60]. In conclusion, variations in the efficacy of vitamin D3 could be due to (a) differences in the cell number, exposure time, and methods of assessment of viability and apoptosis [61]; and/ or (b) the variations in VDR expression among different cancer cell types [62].

The molecular mechanism by which vitamin D3 exerts its anti-proliferative effects on cancer cells remains to be determined. Recent studies on melanoma [63], and breast [64] cancer cell lines suggested that the anti-tumor effects of vitamin D3 are mediated by pathways associated with apoptosis induction [65]. Hence, we examined the impact of vitamin D3 on cancer cell lines for inducing apoptosis at 24h and 48h. In this study, we have analyzed the cell death by ethidium bromide and acridine orange staining method. We observed a visible increase in the number of dead cells in all 4 cancer cell types exposed to vitamin D3. Similar to our data JE Varghese et al. reported an increase in cellular apoptosis in HCT 116 cells upon treatment with vitamin D3 [51] and Bak et al. observed induction of autophagy upon treatment of C6 cells with calcitriol [66]. In summary, vitamin D3 has been shown to promote cell death by increasing various death mechanisms in cancer cells.

Cell cycle regulation has been previously emphasized as one of the mechanisms of action of vitamin D3 [67]. Hence, we investigated the impact of vitamin D3 on cell cycle arrest in two different cell lines (HCT116 and Hep3B). We observed a dose dependent increase in the sub G0 phase (an indicator of dead cells) cell population at 24h of exposure of Hep3B cells. HCT 116 on the other hand showed an increase in sub G0 phase only at 125µM concentration at 24h and 48h treatment. Although, we observed an increase in the sub-G0 cells, we did not detect any changes in the cell cycle pattern.

Since many changes in the apoptotic and cell cycle markers have been outlined with the treatment of cells with anticancer agents, next, we have tested the impact of vitamin D3 on the expression of vitamin D receptor (VDR), and the regulators of apoptosis (such as p53, Bax, Bcl2, and survivin), and cell cycle inhibitors (including p21) in HCT116 and Hep3B. Our study showed an increased VDR expression with low-dose vitamin D3 treatment in both the cell lines at 24h and 48h. The expression of Bax and Bcl2 significantly changed only at the highest dose (125µM) of treatment in both the cell lines after 24h and 48h of treatment. Cyclin D1 showed no significant changes in the expression between untreated and vitamin D3 treated cells. Next, we analyzed the expression pattern of survivin, which is an inhibitor of apoptosis. Results from our study showed a differential expression of survivin with increasing concentrations of vitamin D3 in Hep 3B cell line. HCT 116, however showed a decrease in survivin expression with increasing vitamin D3 concentrations at 24 and 48h. Our result was observed to be consistent with another study by F Li et al., who showed a decrease in the survivin expression in MCF7 cell line when treated with Vitamin D3 [68].

Cell cycle regulators p21 and cyclin D1 expression were also tested in our study. Hep 3B, although showed an increased p21 expression at lower vitamin D3 concentrations it did not affect the cyclin D1 expressions at 24h and 48h of treatment.. HCT 116 displayed a dose-dependent increase in p21 expression at 24h and 48h of treatment with no significant reduction in the cyclin D1 expression. This discrepancy could be due to other factors/proteins responsible for controlling cyclin D1 expression. G Hager et al reported that the active form of vitamin D3 was able to directly regulate the p21 expression [69]. Another study by J Yu et al has shown the regulation of cyclin D1 through VDR regulated Wnt/β-catenin pathway, which has not been explored in our current study [70].

We further tested the effect of vitamin D3 treatment, its dose, and route of administration on tumor growth and progression in mouse models. A study by Jacobson et al demonstrated that rats fed with diets low in calcium and vitamin D3 developed breast tumors more quickly than the rats fed with diets rich in calcium and vitamin D3 [71]. In another study, Mehta et al., found that vitamin D3 analogs could reduce the development of tumors induced by N-methyl-N-nitrosourea (NMU) exposure [72]. Supporting these earlier reports, the data reported in this manuscript further provided strong evidence that intraperitoneal and intra-tumoral administration of vitamin D3 could inhibit the EAC tumors much more effectively compared to just intraperitoneal administration [73]. A prior study evaluated the efficacy of intratumoral dendritic cells in combination with intraperitoneal low dose paclitaxel to treat murine fibrosarcoma and reported complete remission of the tumors when compared to the each drug alone [74]. Even though the i.p. and i.t. combination found effective, its usage in clinical setting is limited as this approach might not be feasible if the tumor cells are metastasized or developed secondary tumors internally. Moreover, this combination approach is not feasible for liquid tumors with many circulating tumor cells.

The Ehrlich Ascites Carcinoma (EAC) model is used widely in cancer research for understanding and exploring the cancer biology and efficacy of anti-cancer agents [75]. In line with the earlier reported studies, our study also showed that vitamin D3 was able to reduce the EAC solid tumors volume. No changes in the serum calcium were observed, however, vitamin D3 treatment increased serum phosphorus levels. This could be a result of low parathyroid hormone, renal failure, or excessive vitamin D3 dosage [76].

Since vitamin D3 has been reported to regulate lipids in the plasma, we have analyzed the HDL, Total Cholesterol (TC), and Triglyceride (TG) levels [77]. In our study, we observed that treatment with vitamin D3 increased triglycerides and decreased cholesterol and HDL levels. This finding of increased HDL is consistent with the study by Ponder et al., which demonstrated that an increase in vitamin D3 leads to an increase in HDL [78]. In another study by Akbari et al., the vitamin D3 treatment showed no beneficial effects on TG, TC, or HDL levels [79].

There is growing evidence linking type 2 diabetes mellitus (T2DM) to a higher chance of developing cancer [80]. In addition to the risk factors that are common to both diseases, diabetic individuals are known to have low levels of vitamin D3, which is similar to cancer patients [81]. Thus, making the diabetes individuals more susceptible to developing cancer and exhibiting treatment resistance. Furthermore, hyperglycaemia and insulin resistance, which are the hallmark features of diabetes, have been implicated in promoting cancer growth and metastasis through various biological mechanisms [82]. Since studies have suggested that diabetes alone can be considered as an independent risk factor for the development of cancer, we developed a model to study the growth properties of EAC cells in diabetic mice. Diabetes was induced by administering low-dose Streptozotocin. Low-dose STZ is an ideal and quick model for creating a type-2 diabetes condition [83]. Once the diabetes status is confirmed, EAC cells were injected for the induction of tumors. In this model, a non-significant increase in the tumor volume was observed in mice injected with STZ, indicating that hyperglycemia might be promoting the growth of EAC tumors in mice. Administration of vitamin D3 has reduced the growth of tumors even in diabetic mice, as there was a significant decrease in the tumor volume compared to tumor bearing control mice

In summary, we have demonstrated that vitamin D3 is effective in reducing the viability of cancer cells by promoting cell death. In addition, we have shown that vitamin D3 retards the EAC tumors growth when administered intraperitoneally, with even greater efficacy when administered through both intraperitoneal and intra-tumoral routes simultaneously. Even though our study has shown the ability of vitamin D3 against multiple cancer cell lines and EAC tumors, there are certain limitations, which can be explored by the interested research community. For example, the mechanistic pathways by which vitamin D3 exerts its anti-tumor effects are not studied in detail. Similarly, the impact of vitamin D3 administration on the gut microbiome and the products produced by the gut microbiome are not explored. The intra-tumoral concentration of vitamin D3 and the metabolic products produced by vitamin D3 in the vicinity of tumors are not tested in this study. Addressing these unanswered questions will help in better understanding the impact of vitamin D3 and its anti-tumoral properties.

## Conclusions

In conclusion our study showed that treatment of a panel of cancer cell lines with vitamin D3 reduced the viability by inducing apoptosis mediated via caspase-3 activation. Cell cycle analysis did not show any significant changes upon vitamin D3 treatment, but a noticeable increase in sub-G0-G1 cell population suggests apoptotic cell death. Western blot analysis showed the induction of cell death by vitamin D3, which is driven primarily through increased Bax/Bcl2 ratio and decreased survivin expression. In mice, we found that a combination of intraperitoneal and intratumoral administration of vitamin D3 is more effective in retarding EAC solid tumors compared to intraperitoneal administration alone. Furthermore, we found that vitamin D3 could retard tumors even in diabetic mice. Mechanistically, vitamin D3 reduced tumor cells proliferation by destroying blood vessels and promoting apoptosis. In summary, vitamin D3 is a potent anti-tumor agent, but require additional optimization procedures to deliver it more effectively into tumors.

## Supporting information

S1 Fig Dose and time dependent decrease in the viability of cancer cells upon treatment with vitamin D3. a. Liver cancer cell line Hep G2 was exposed to increasing concentrations of vitamin D3 and its impact on reducing the viability was determined using SRB assay at 24h, 48h and 72h. A dose dependent increase in cytotoxic effect was observed with increasing vitamin D3 concentration. Hep G2 exhibited more sensitivity to vitamin D3 treatment compared to Hep 3B. b.Colorectal carcinoma cell line HT-29 was treated with increasing concentration of vitamin D3 for 24h, 48h and 72h and the number of viable cells determined by SRB assay. The data showed a dose dependent response upon treatment with vitamin D3 at 24h of exposure. However, continued exposure to 48h and 72h led to decreased efficacy of this sunshine vitamin. c.HPV 16 positive cell line SiHa was treated with vitamin D3 as detailed in methods section and the viability measured by SRB assay. Vitamin D3 inhibited the viability of SiHa cell line beginning from 62.5µM. The percentage reduction in the viability has reduced with increasing concentration of vitamin D (from 250µM). Prolonged treatment time resulted in a slightly better cytotoxic effect.
(TIF)

S2 Fig Treatment of C6 cells with vitamin D3 reduced the number of cells in a time and dose dependent fashion. Treatment of C6 cell line with vitamin D3 at different time points and concentrations showed a time and dose dependent reduction in cell number. The reduction in cell number was evident at a dose of 62.5 µM and beyond at 48 and 72h treatment.
(TIF)

S3 Fig Treatment of cancer cells with vitamin D3 induced death *a.* Treatment of Hep G2 cells with vitamin D3 induced cell death in a dose- and time dependent manner. Whereas about 40% dead cells were observed at 125µM vitamin D3 at 24h, similar percentage cells were dead at 62.5µM at 48h. Cisplatin (100µM), which is used as a positive control, yielded ~40% and ~90% cell death respectively at 24h and 48h. The bar graph represents the mean of at least 3 fields with SEM. One-way ANOVA was applied to determine the significance among control and experimental groups. “P” value <0.05 was considered significant. b.Treatment of HT-29 cells with vitamin D3 induced cell death in a dose- and time dependent manner. Whereas about 70% dead cells were observed at 250µM vitamin D3 at 24h, similar percentage cells were dead at 125µM at 48h. Cisplatin (100µM), which is used as a positive control, yielded ~25% and ~40% cell death respectively at 24h and 48h. The bar graph represents the mean of at least 3 fields with SEM. One-way ANOVA was applied to determine the significance among control and experimental groups. “P” value <0.05 was considered significant. c.Treatment of SiHa cells with vitamin D3 induced cell death in a dose- and time dependent manner. Whereas about 85% dead cells were observed at 250µM vitamin D3 at 24h, similar percentage cells were dead at 62.5µM at 48h. Cisplatin (100µM), which is used as a positive control, yielded ~80% and ~95% cell death respectively at 24h and 48h. The bar graph represents the mean of at least 3 fields with SEM. One-way ANOVA was applied to determine the significance among control and experimental groups. “P” value <0.05 was considered significant.
(TIF)

S4 Fig Exposure of cancer cells to vitamin D3 increased sub-G0-G1 cell population. a. Treatment of Hep 3B cells with vitamin D3 showed an increase in the sub G0 phase population at 24h of treatment. Vitamin D3 (125µM) showed a better effect in inducing apoptosis when compared to the positive control Camptothecin (25nM). b Prolonged exposure of Hep 3B cells to vitamin D3 (for 48h) showed an increase in sub G0 cells at the higher concentrations of 62.5. µM and 125 µM c. and d. HCT 116 cells treated with vitamin D3 for 24h and 48h showed increased Sub G0 cells at 125 µM concentration at 24 and 48h of treatment.
(TIF)

S5 Fig Vitamin D3, but not the Cisplatin, could moderately reduce STZ-induced hyperglycemia in mice (a) Schematic representation of experimental protocol followed in the study: After acclimatization, mice were administered with STZ (2 X 30 mg/kg and 2 X 40 mg/kg) to induce hyperglycemia. The control non-diabetic group was administered with the vehicle used for dissolving the STZ, i.e., 100mM sodium citrate buffer, pH 4.5. After confirming the induction of hyperglycemia in STZ group (diabetic mice), both control non-hyperglycemic mice and hyperglycemic mice were injected intramuscularly with EAC cells to create solid tumors (Day 14). Beginning from day 28, when the solid tumors are of ~100mm^3^ size, control and experimental mice were administered with drugs every other day for 18 days (total duration 46 days); on day 46, the mice were sacrificed and blood, vital organs and tumors were harvested for further analysis (b) Administration of STZ increased fasting blood sugar in mice: In order to establish hyperglycaemic state, mice were administered with low-dose STZ as detailed in methods section and blood sugar content determined using Morepen glucose monitoring strips. The data showed a significant increase in fasting blood glucose (FBG) beginning from day 6. The hyperglycaemic state was maintained till the end of the study as represented by the elevated blood glucose levels measured on the last day of the treatment regime. (c) Vitamin D3 and the positive control Cisplatin differently modulated FBG in hyperglycaemic mice: Intraperitoneal administration of vitamin D3 very minimally decreased FBG compared to vehicle control at the end of the study. Interestingly the FBG is increased in the mice treated with cisplatin (2.5 mg/Kg) in the final two time points.
(TIF)

S6 Fig Schematic representation of tumor kinetics study in diabetic and non-diabetic mice. The scheme represents the induction of a STZ induced diabetes model where low doses of STZ was administered for 4 days(30 mg/Kg and 40 mg/Kg i.p.). While the non diabetic animals received 0.1M citrate buffer. Upon induction of diabetes EAC cells were injected intramuscularly in the thigh region of the left leg of mice on day 14 and allowed to develop in diabetic and non diabetic animals. The volume of developing tumors was measured in both the models until day 47 and the data plotted.
(TIF)

S7 Fig Schematic representation of vitamin D3 treatment schedule in diabetic and non-diabetic mice. In order to determine the impact of administering Vitamin D3 (125 mg/Kg) on EAC tumors growth and blood parameters, the in vivo study was conducted as detailed before. Beginning from day 28, the control non-hyperglycaemic and experimental hyperglycaemic mice were administered with vitamin D3 every other day till day 46. On day 47, the mice were sacrificed and the vital organs and blood were collected for further processing.
(TIF)

S8 Fig Schematic representation of treatment schedule with Cisplatin. In order to determine the impact of administering Cisplatin (2.5 mg/Kg) on EAC tumors growth and blood parameters, the in vivo study was conducted as detailed before. Beginning from day 28, the control non-hyperglycaemic and experimental hyperglycaemic mice were administered with Cisplatin every other day till day 46. On day 47, the mice were sacrificed and the vital organs and blood were collected for further processing.
(TIF)

S9 Fig Schematic representation of vitamin D3 administration routes. After completion of acclimatization, animals were injected with EAC cells on the 14^th^ day and allowed to develop into solid tumors. Day 28 onwards vitamin D3 was administered once every alternate day. Doses were administered intraperitoneal alone, intraperitoneal and intratumoral for 8 doses. Tumor volumes of the animals were continuously monitored during the dosing regimen. Mice were then sacrificed after the completion of the study.
(TIF)

S10 Fig (a to g) Administration of vitamin D3 increased serum phosphorus and triglycerides. In order to determine the changes in serum biochemical parameters that include FBG, Total Cholesterol, HDL, TG, Calcium, Phosphorus and the vitamin D level, the blood was collected from mice at the end of the experiment and subjected to analysis as detailed in methods section. Analysis of the serum showed no significant changes in the serum vitamin D3 or calcium levels in the treated group when compared to the normal control. But, the serum phosphorous had significantly increased in the vitamin D3 treated group compared to normal control. Vitamin D3 administration did not change serum random glucose levels tested at the end of the treatment period. Analyses of serum cholesterol, HDL and triglycerides also showed a non-significant decrease in the serum HDL level upon treatment with 125 µg/Kg vitamin D3, but a noticeable increase in the serum triglyceride level was observed with 125 µg/Kg of vitamin D3.
(TIF)

S11 Fig Variations in the morphology and tissue architecture of EAC-solid tumors. In order to determine tissue architectural and morphological changes in the tumor, the tumors were harvested and processed as detailed in methods section. The tumors were sectioned and stained with Hematoxylin and Eosin. The stained sections were observed under microscope and changes in the tissues recorded by pathologists. Photomicrographs are representatives of six different tumors.
Supporting Information
(TIF)

S1 File Minimal Data set. (DOCX)

S1 Image Raw images. (PDF)
