# Supplementary figures and images for: Vitamin D3 reduces the viability of cancer cells in vitro and retard the EAC tumors growth in mice
Source: PLoS One. 2025 Sep 8;20(9):e0331306. doi: 10.1371/journal.pone.0331306 (PMC12416751; doi:10.1371/journal.pone.0331306)

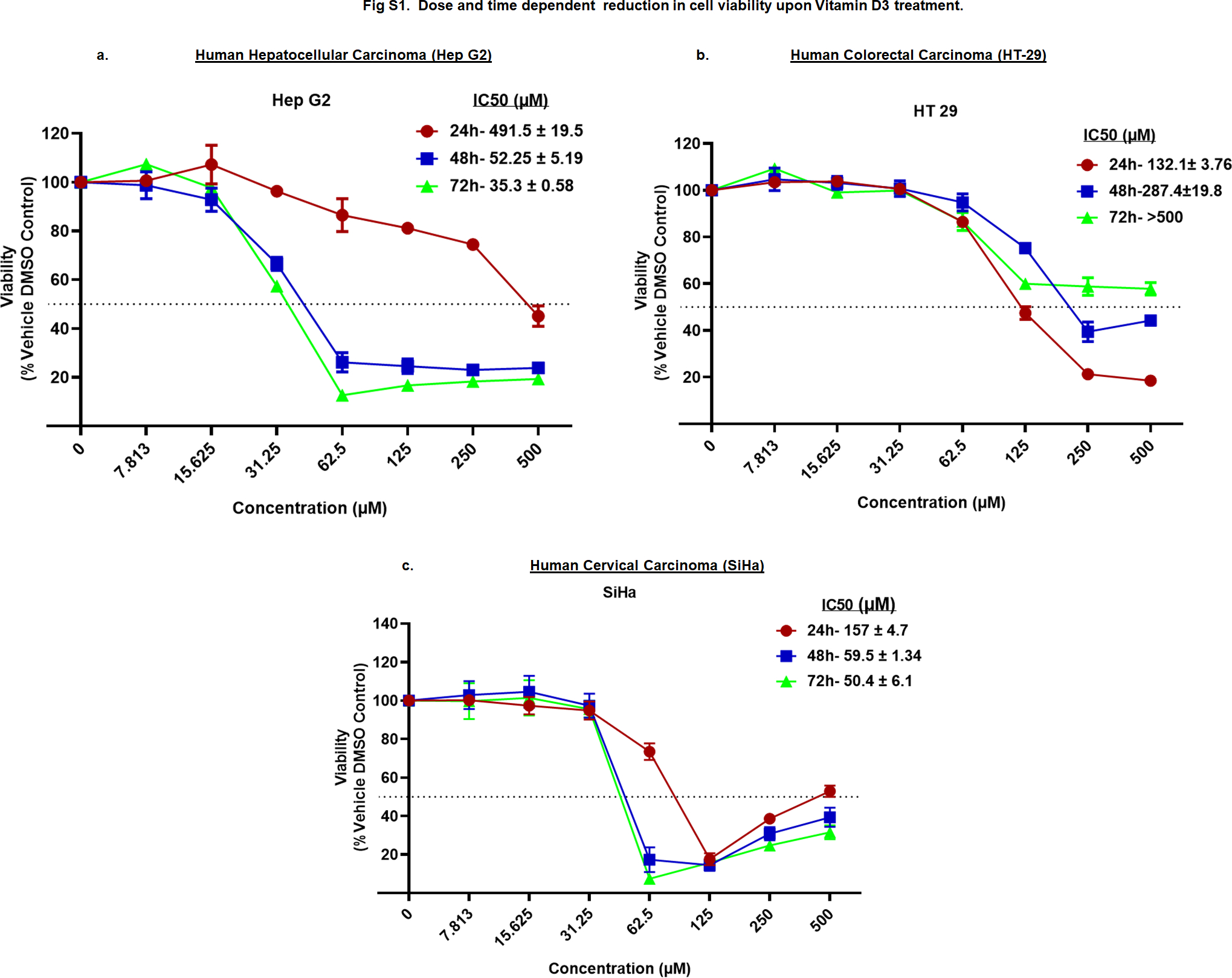

Supplement: S1 Fig — a. Liver cancer cell line Hep G2 was exposed to increasing concentrations of vitamin D3 and its impact on reducing the viability was determined using SRB assay at 24h, 48h and 72h. A dose dependent increase in cytotoxic effect was observed with increasing vitamin D3 concentration. Hep G2 exhibited more sensitivity to vitamin D3 treatment compared to Hep 3B. b.Colorectal carcinoma cell line HT-29 was treated with increasing concentration of vitamin D3 for 24h, 48h and 72h and the number of viable cells determined by SRB assay. The data showed a dose dependent response upon treatment with vitamin D3 at 24h of exposure. However, continued exposure to 48h and 72h led to decreased efficacy of this sunshine vitamin. c.HPV 16 positive cell line SiHa was treated with vitamin D3 as detailed in methods section and the viability measured by SRB assay. Vitamin D3 inhibited the viability of SiHa cell line beginning from 62.5µM. The percentage reduction in the viability has reduced with increasing concentration of vitamin D (from 250µM). Prolonged treatment time resulted in a slightly better cytotoxic effect. (TIF) [file pone.0331306.s001.tif]

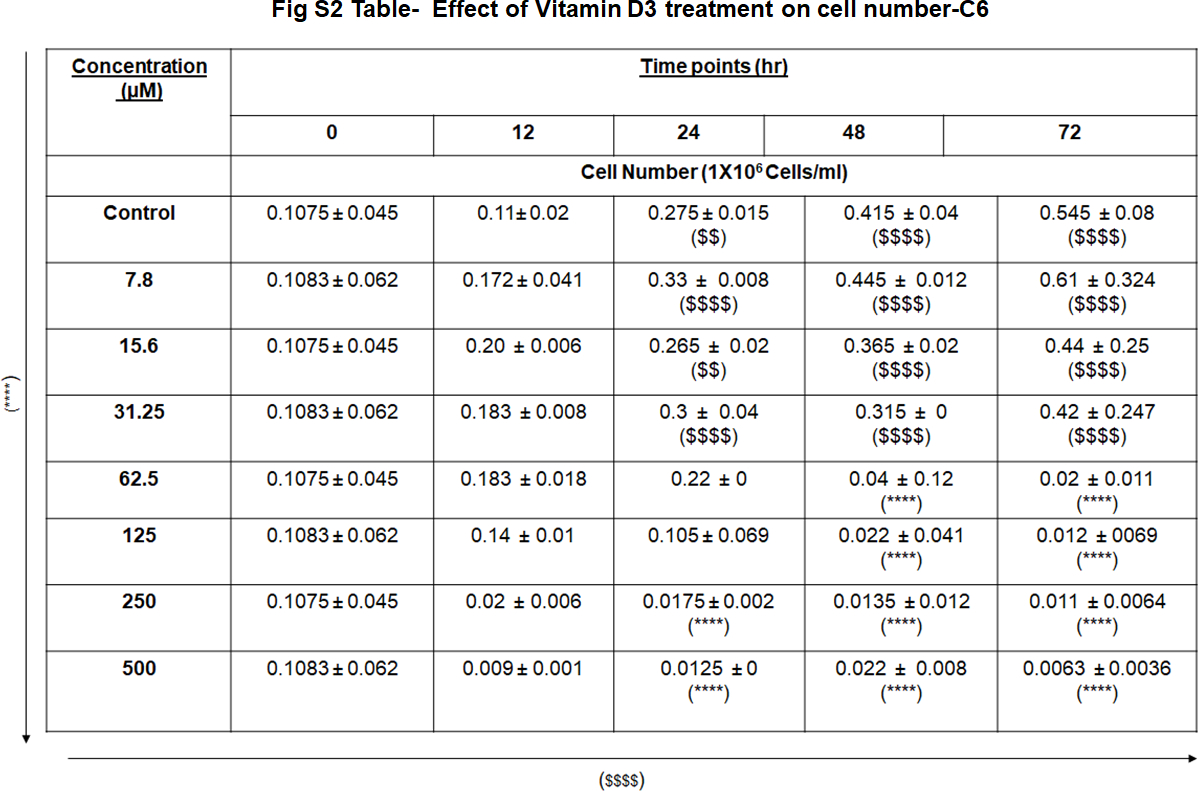

Supplement: S2 Fig — Treatment of C6 cell line with vitamin D3 at different time points and concentrations showed a time and dose dependent reduction in cell number. The reduction in cell number was evident at a dose of 62.5 µM and beyond at 48 and 72h treatment. (TIF) [file pone.0331306.s002.tif]

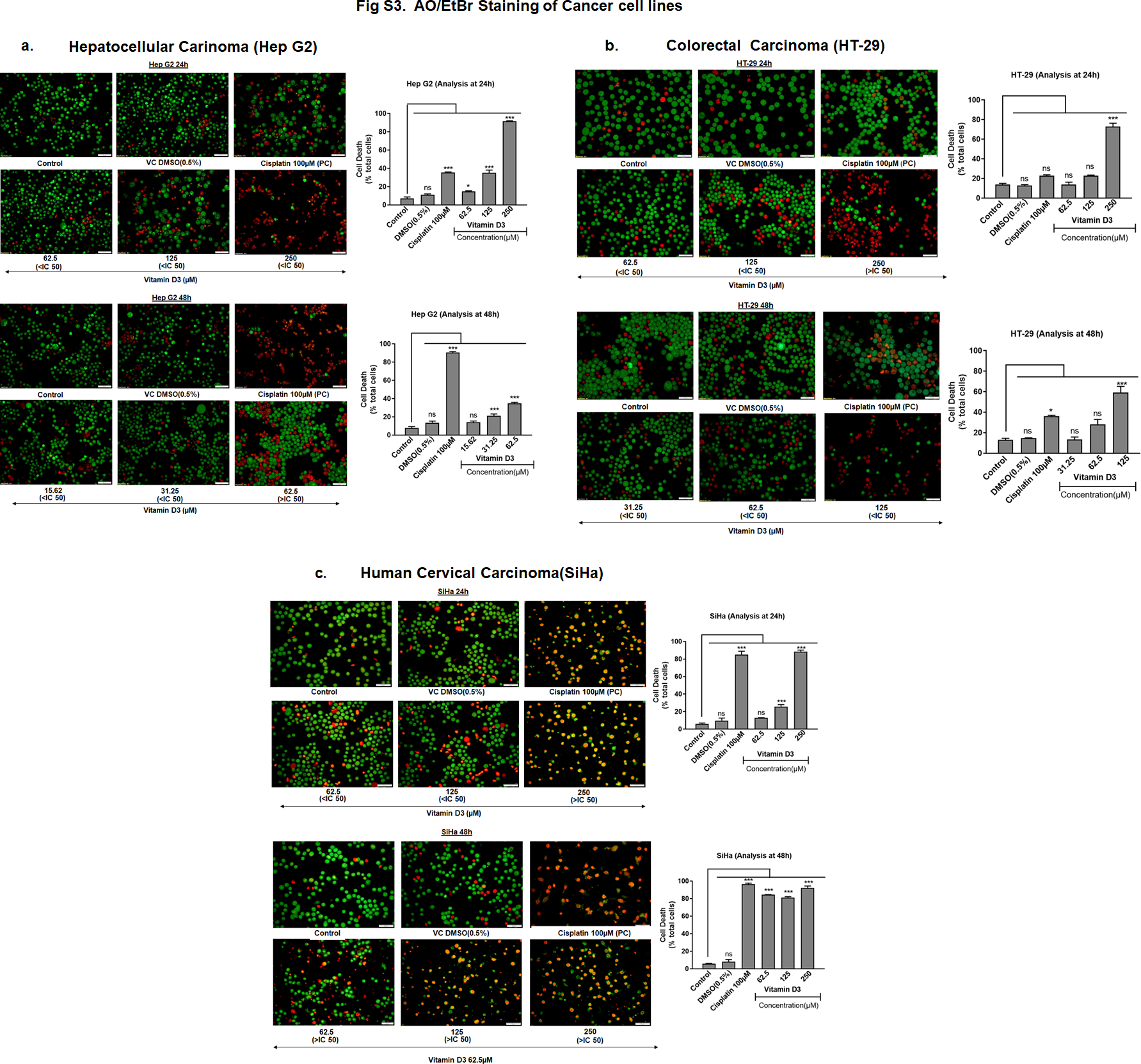

Supplement: S3 Fig — Treatment of Hep G2 cells with vitamin D3 induced cell death in a dose- and time dependent manner. Whereas about 40% dead cells were observed at 125µM vitamin D3 at 24h, similar percentage cells were dead at 62.5µM at 48h. Cisplatin (100µM), which is used as a positive control, yielded ~40% and ~90% cell death respectively at 24h and 48h. The bar graph represents the mean of at least 3 fields with SEM. One-way ANOVA was applied to determine the significance among control and experimental groups. “P” value <0.05 was considered significant. b.Treatment of HT-29 cells with vitamin D3 induced cell death in a dose- and time dependent manner. Whereas about 70% dead cells were observed at 250µM vitamin D3 at 24h, similar percentage cells were dead at 125µM at 48h. Cisplatin (100µM), which is used as a positive control, yielded ~25% and ~40% cell death respectively at 24h and 48h. The bar graph represents the mean of at least 3 fields with SEM. One-way ANOVA was applied to determine the significance among control and experimental groups. “P” value <0.05 was considered significant. c.Treatment of SiHa cells with vitamin D3 induced cell death in a dose- and time dependent manner. Whereas about 85% dead cells were observed at 250µM vitamin D3 at 24h, similar percentage cells were dead at 62.5µM at 48h. Cisplatin (100µM), which is used as a positive control, yielded ~80% and ~95% cell death respectively at 24h and 48h. The bar graph represents the mean of at least 3 fields with SEM. One-way ANOVA was applied to determine the significance among control and experimental groups. “P” value <0.05 was considered significant. (TIF) [file pone.0331306.s003.tif]

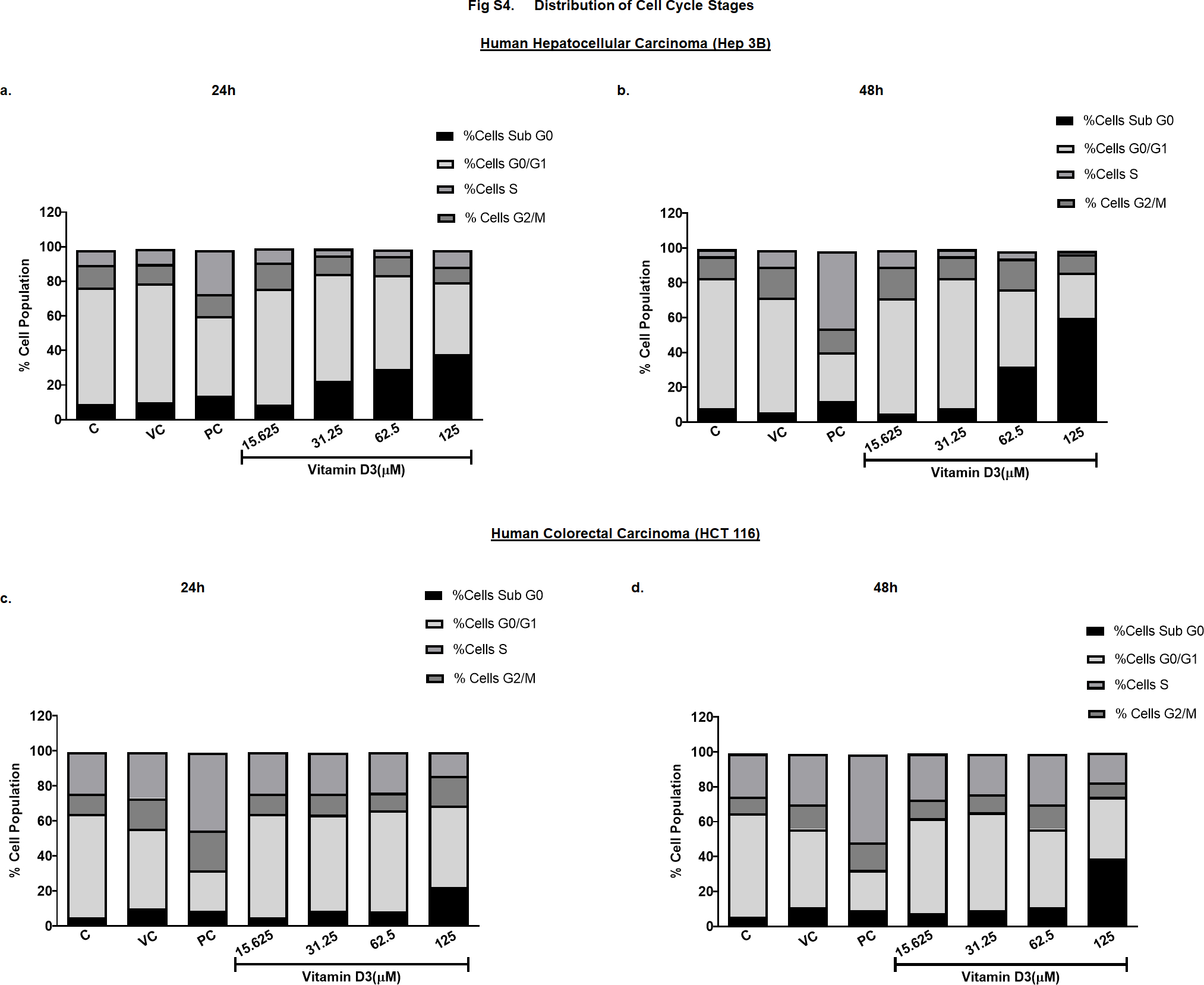

Supplement: S4 Fig — a. Treatment of Hep 3B cells with vitamin D3 showed an increase in the sub G0 phase population at 24h of treatment. Vitamin D3 (125µM) showed a better effect in inducing apoptosis when compared to the positive control Camptothecin (25nM). b Prolonged exposure of Hep 3B cells to vitamin D3 (for 48h) showed an increase in sub G0 cells at the higher concentrations of 62.5. µM and 125 µM c. and d. HCT 116 cells treated with vitamin D3 for 24h and 48h showed increased Sub G0 cells at 125 µM concentration at 24 and 48h of treatment. (TIF) [file pone.0331306.s004.tif]

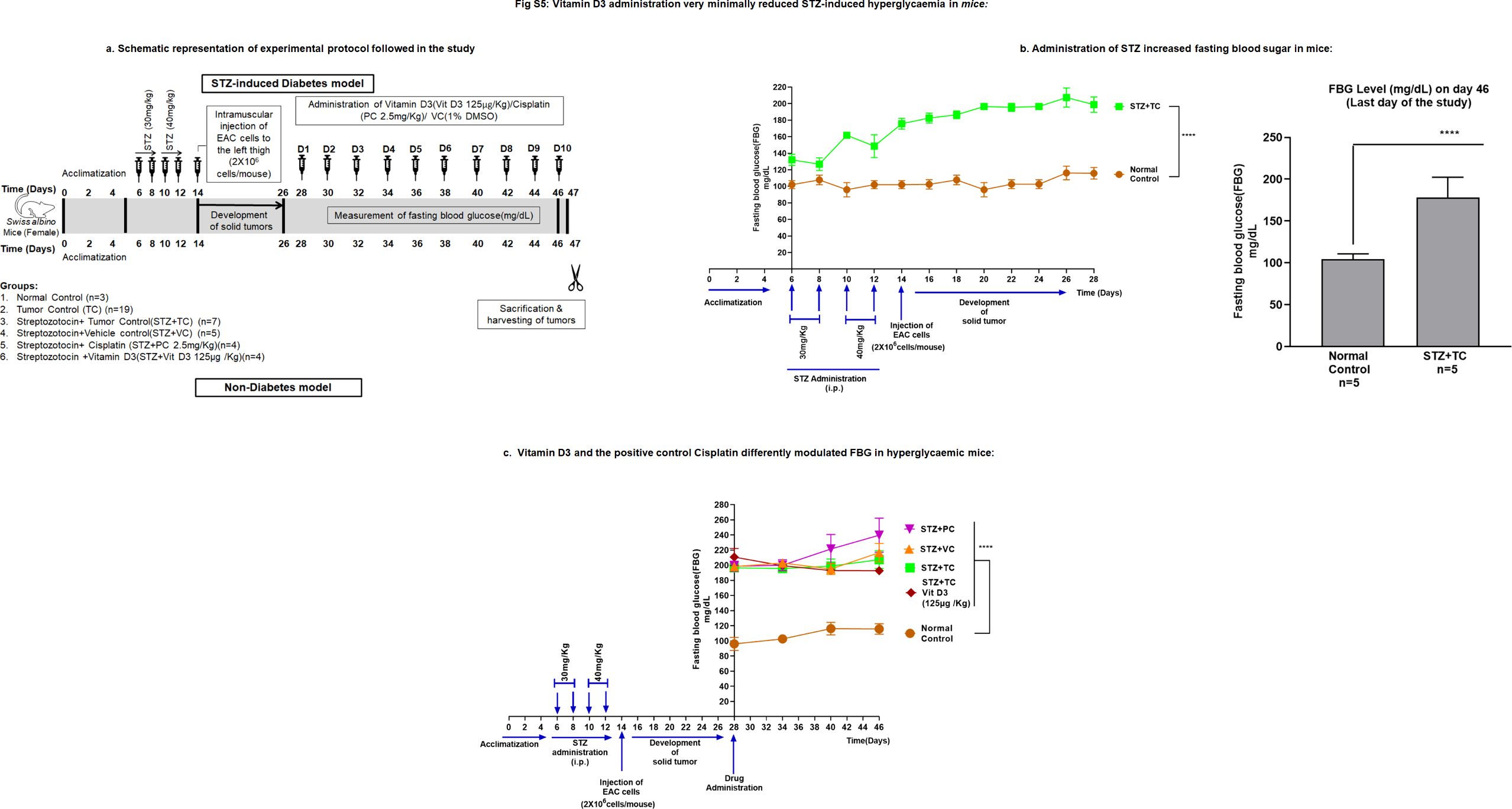

Supplement: S5 Fig — The control non-diabetic group was administered with the vehicle used for dissolving the STZ, i.e., 100mM sodium citrate buffer, pH 4.5. After confirming the induction of hyperglycemia in STZ group (diabetic mice), both control non-hyperglycemic mice and hyperglycemic mice were injected intramuscularly with EAC cells to create solid tumors (Day 14). Beginning from day 28, when the solid tumors are of ~100mm3 size, control and experimental mice were administered with drugs every other day for 18 days (total duration 46 days); on day 46, the mice were sacrificed and blood, vital organs and tumors were harvested for further analysis (b) Administration of STZ increased fasting blood sugar in mice: In order to establish hyperglycaemic state, mice were administered with low-dose STZ as detailed in methods section and blood sugar content determined using Morepen glucose monitoring strips. The data showed a significant increase in fasting blood glucose (FBG) beginning from day 6. The hyperglycaemic state was maintained till the end of the study as represented by the elevated blood glucose levels measured on the last day of the treatment regime. (c) Vitamin D3 and the positive control Cisplatin differently modulated FBG in hyperglycaemic mice: Intraperitoneal administration of vitamin D3 very minimally decreased FBG compared to vehicle control at the end of the study. Interestingly the FBG is increased in the mice treated with cisplatin (2.5 mg/Kg) in the final two time points. (TIF) [file pone.0331306.s005.tif]

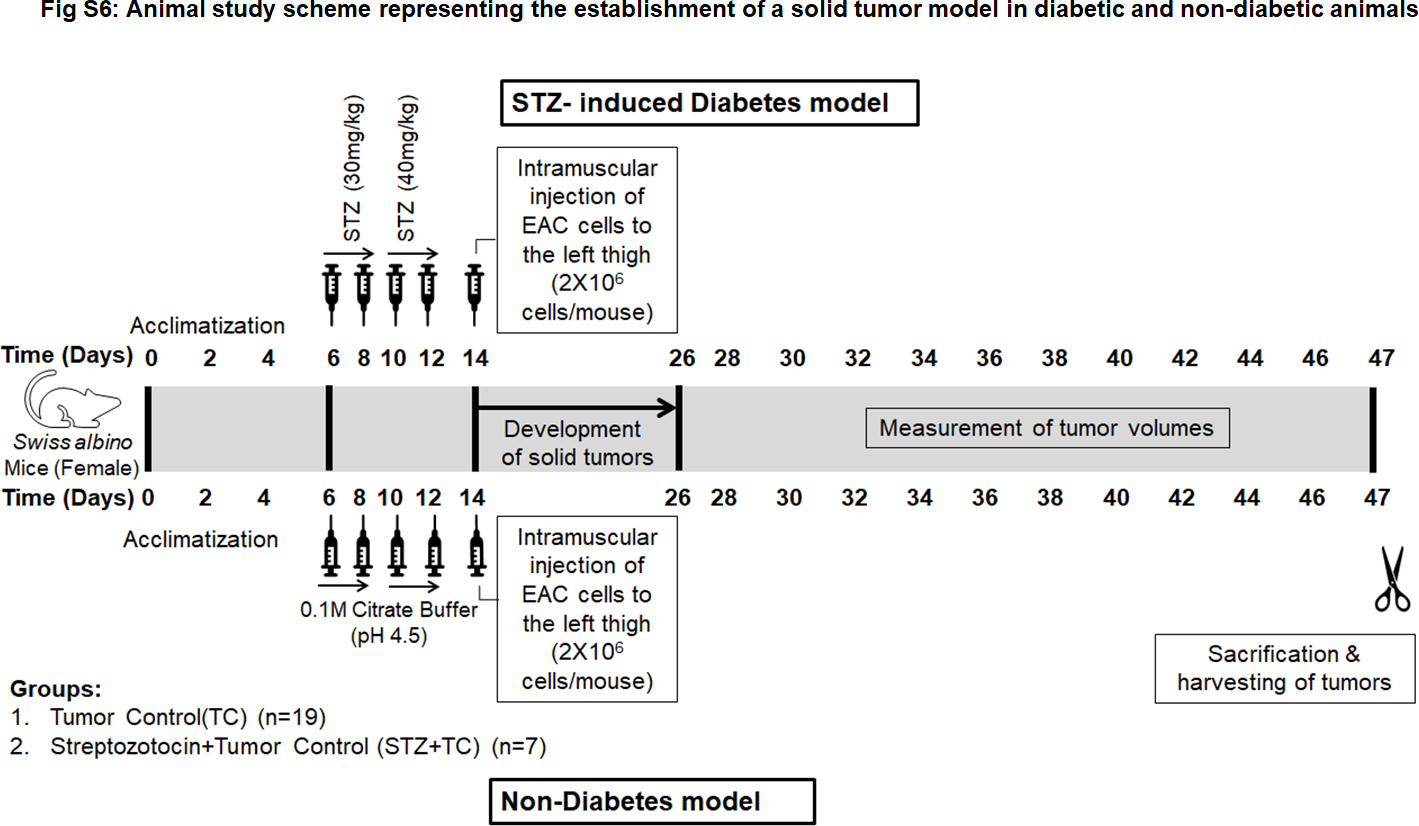

Supplement: S6 Fig — The scheme represents the induction of a STZ induced diabetes model where low doses of STZ was administered for 4 days(30 mg/Kg and 40 mg/Kg i.p.). While the non diabetic animals received 0.1M citrate buffer. Upon induction of diabetes EAC cells were injected intramuscularly in the thigh region of the left leg of mice on day 14 and allowed to develop in diabetic and non diabetic animals. The volume of developing tumors was measured in both the models until day 47 and the data plotted. (TIF) [file pone.0331306.s006.tif]

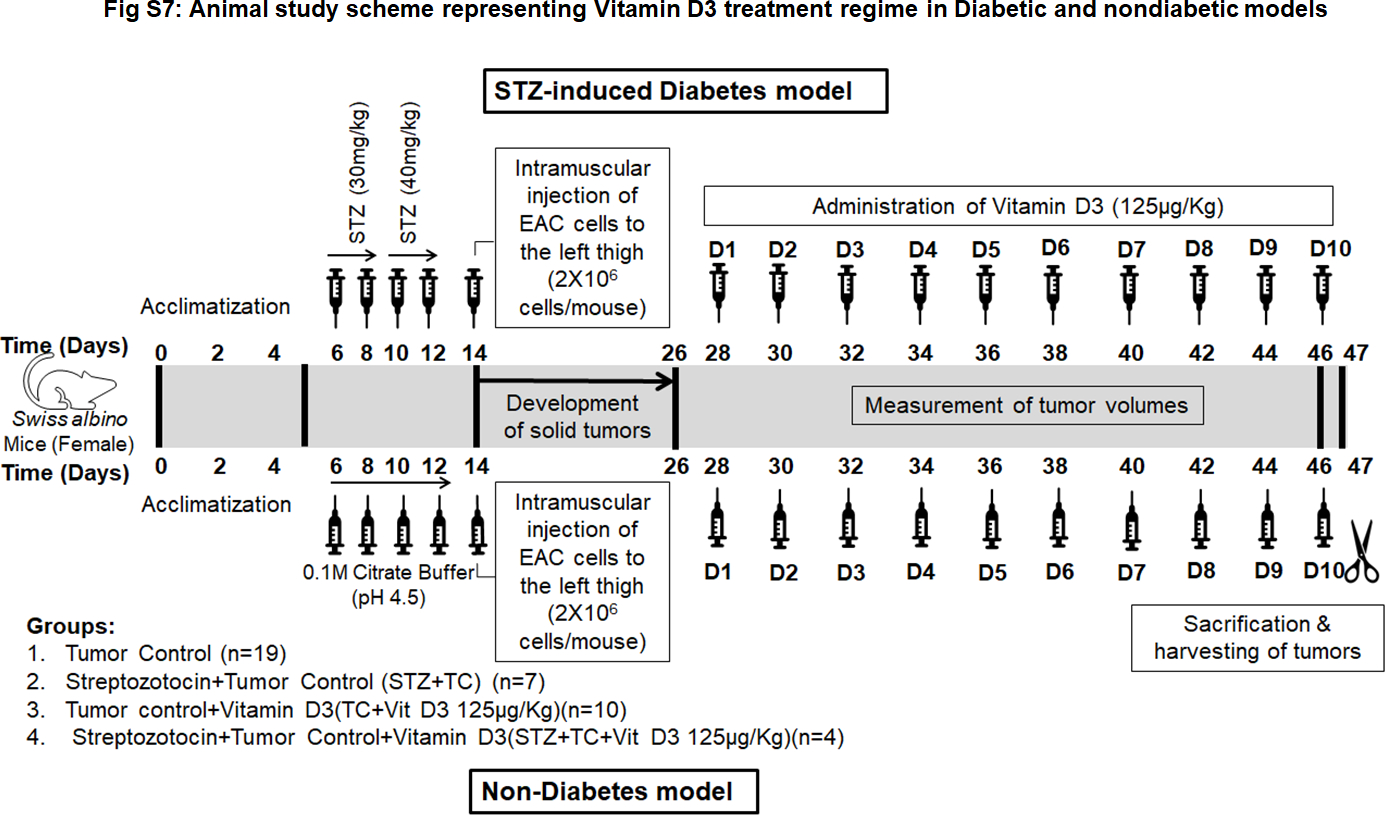

Supplement: S7 Fig — In order to determine the impact of administering Vitamin D3 (125 mg/Kg) on EAC tumors growth and blood parameters, the in vivo study was conducted as detailed before. Beginning from day 28, the control non-hyperglycaemic and experimental hyperglycaemic mice were administered with vitamin D3 every other day till day 46. On day 47, the mice were sacrificed and the vital organs and blood were collected for further processing. (TIF) [file pone.0331306.s007.tif]

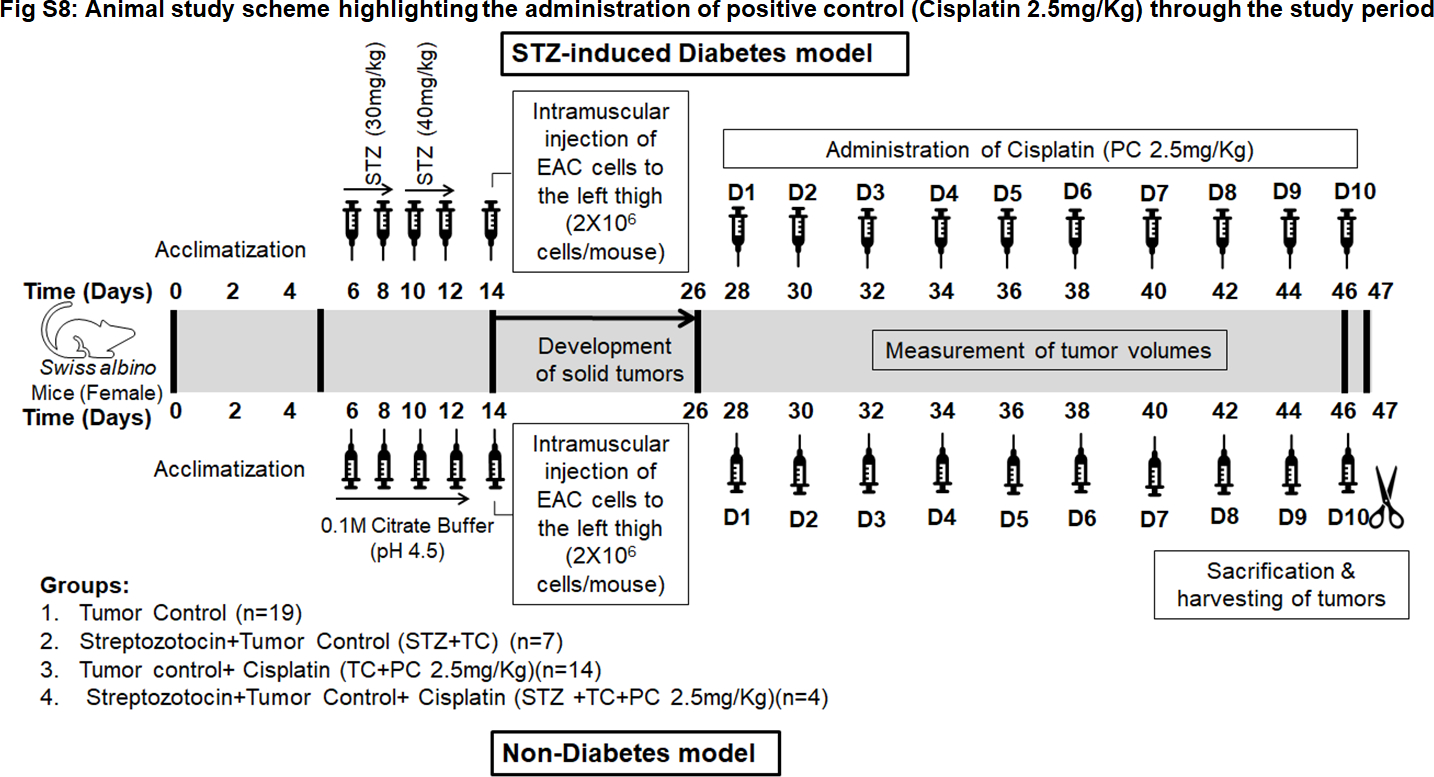

Supplement: S8 Fig — In order to determine the impact of administering Cisplatin (2.5 mg/Kg) on EAC tumors growth and blood parameters, the in vivo study was conducted as detailed before. Beginning from day 28, the control non-hyperglycaemic and experimental hyperglycaemic mice were administered with Cisplatin every other day till day 46. On day 47, the mice were sacrificed and the vital organs and blood were collected for further processing. (TIF) [file pone.0331306.s008.tif]

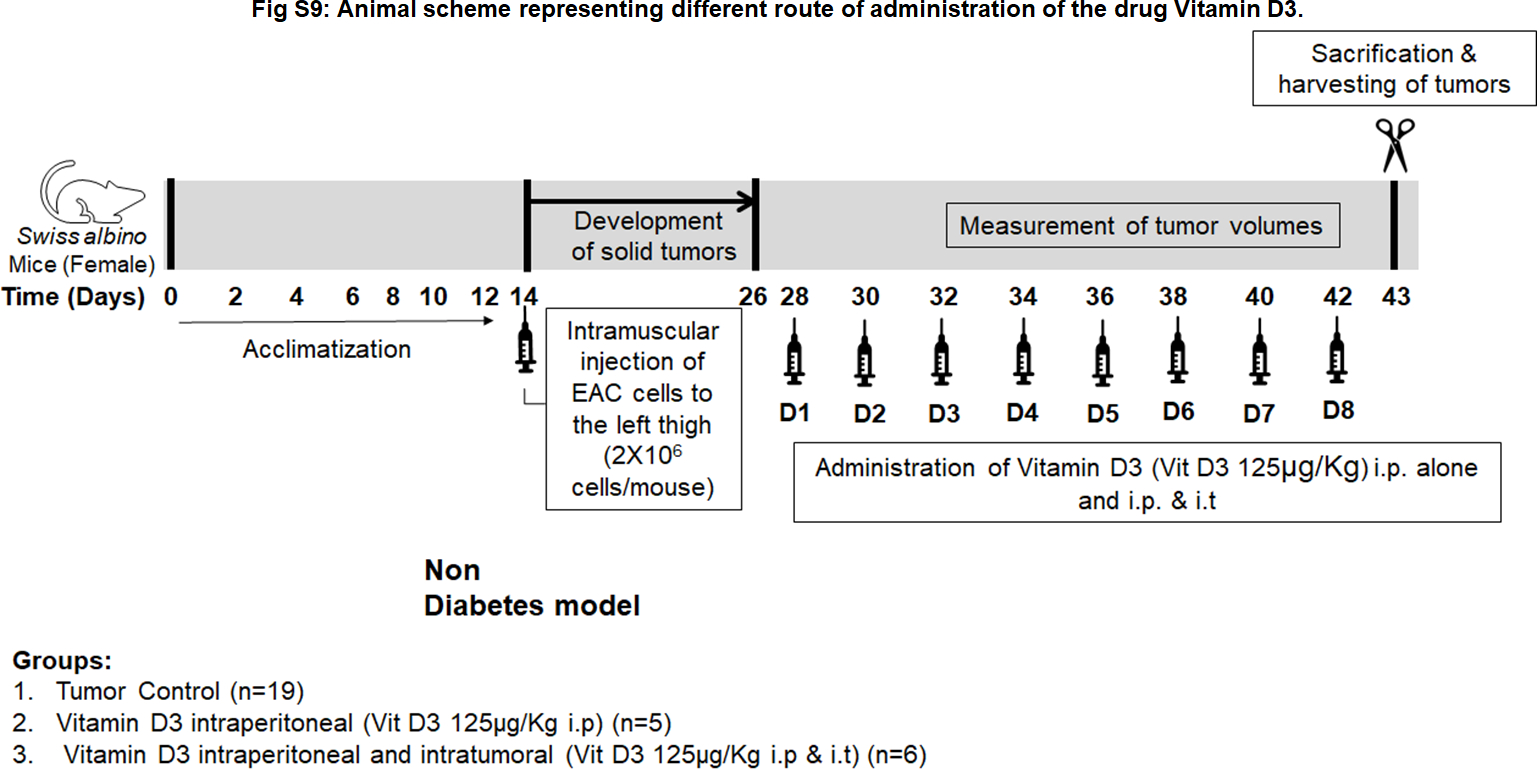

Supplement: S9 Fig — After completion of acclimatization, animals were injected with EAC cells on the 14th day and allowed to develop into solid tumors. Day 28 onwards vitamin D3 was administered once every alternate day. Doses were administered intraperitoneal alone, intraperitoneal and intratumoral for 8 doses. Tumor volumes of the animals were continuously monitored during the dosing regimen. Mice were then sacrificed after the completion of the study. (TIF) [file pone.0331306.s009.tif]

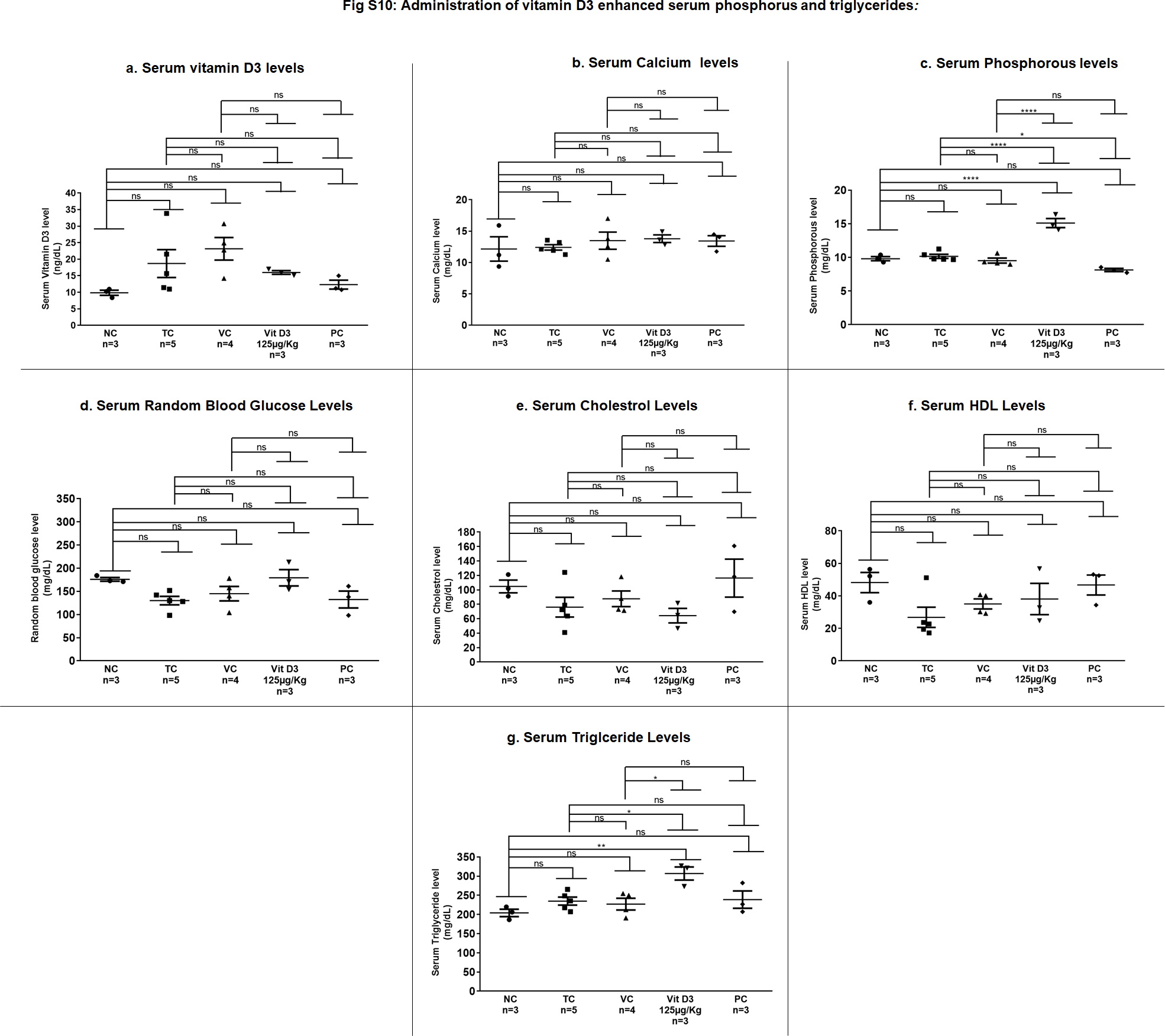

Supplement: S10 Fig — In order to determine the changes in serum biochemical parameters that include FBG, Total Cholesterol, HDL, TG, Calcium, Phosphorus and the vitamin D level, the blood was collected from mice at the end of the experiment and subjected to analysis as detailed in methods section. Analysis of the serum showed no significant changes in the serum vitamin D3 or calcium levels in the treated group when compared to the normal control. But, the serum phosphorous had significantly increased in the vitamin D3 treated group compared to normal control. Vitamin D3 administration did not change serum random glucose levels tested at the end of the treatment period. Analyses of serum cholesterol, HDL and triglycerides also showed a non-significant decrease in the serum HDL level upon treatment with 125 µg/Kg vitamin D3, but a noticeable increase in the serum triglyceride level was observed with 125 µg/Kg of vitamin D3. (TIF) [file pone.0331306.s010.tif]

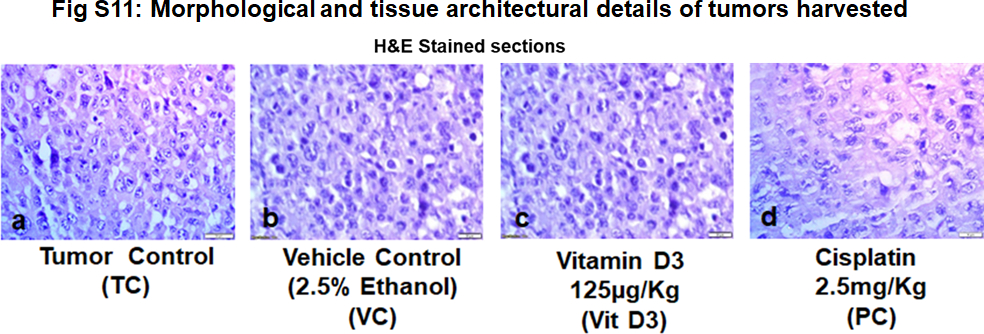

Supplement: S11 Fig — In order to determine tissue architectural and morphological changes in the tumor, the tumors were harvested and processed as detailed in methods section. The tumors were sectioned and stained with Hematoxylin and Eosin. The stained sections were observed under microscope and changes in the tissues recorded by pathologists. Photomicrographs are representatives of six different tumors. Supporting Information (TIF) [file pone.0331306.s011.tif]
