# Supplementary material for: Vitamin D3 reduces the viability of cancer cells in vitro and retard the EAC tumors growth in mice
Source: PLoS One. 2025 Sep 8;20(9):e0331306. doi: 10.1371/journal.pone.0331306 (PMC12416751; doi:10.1371/journal.pone.0331306)
Supplement: S1 File — (DOCX) [file pone.0331306.s012.docx]

**Main text: Raw values with Mean, SD and SEM**

Fig 1A: Human Keratinocyte- HaCaT

|  | **24h** | | | | |
| --- | --- | --- | --- | --- | --- |
| **Concentration (µM)** | **1** | **2** | **Mean** | **SD** | **SEM** |
| **25** | 101.74 | 99.92 | 100.83 | 1.28 | 0.91 |
| **50** | 97.87 | 108.76 | 103.31 | 7.69 | 5.45 |
| **100** | 93.25 | 100.85 | 97.05 | 5.37 | 3.80 |
| **200** | 90.15 | 97.51 | 93.83 | 5.20 | 3.69 |
| **400** | 95.30 | 96.51 | 95.90 | 0.85 | 0.60 |

|  | **48h** | | | | |
| --- | --- | --- | --- | --- | --- |
| **Concentration**  **(µM)** | **1** | **2** | **Mean** | **SD** | **SEM** |
| **25** | 77.02 | 92.24 | 84.63 | 10.76 | 7.63 |
| **50** | 86.60 | 99.39 | 92.99 | 9.042 | 6.41 |
| **100** | 88.01 | 90.54 | 89.27 | 1.78 | 1.26 |
| **200** | 82.56 | 93.07 | 87.81 | 7.42 | 5.26 |
| **400** | 81.64 | 92.68 | 87.16 | 7.80 | 5.53 |

Fig 1B: Human Lung Epithelial-BEAS-2B

|  | **24h** | | | | |
| --- | --- | --- | --- | --- | --- |
| **Concentration**  **(µM)** | **1** | **2** | **Mean** | **SD** | **SEM** |
| **25** | 101.05 | 84.39 | 92.72 | 11.78 | 8.35 |
| **50** | 85.80 | 91.90 | 88.85 | 4.31 | 3.05 |
| **100** | 83.80 | 92.19 | 88.00 | 5.93 | 4.20 |
| **200** | 92.42 | 88.97 | 90.70 | 2.44 | 1.73 |
| **400** | 85.06 | 86.73 | 85.90 | 1.17 | 0.83 |

|  | **48h** | | | | |
| --- | --- | --- | --- | --- | --- |
| **Concentration**  **(µM)** | **1** | **2** | **Mean** | **SD** | **SEM** |
| **25** | 86.05 | 89 | 87.52 | 2.08 | 1.47 |
| **50** | 104.34 | 106.54 | 105.44 | 1.55 | 1.10 |
| **100** | 96.97 | 100.43 | 98.70 | 2.44 | 1.73 |
| **200** | 115.13 | 118.35 | 116.74 | 2.28 | 1.61 |
| **400** | 98.618 | 95.32 | 96.97 | 2.32 | 1.64 |

Fig 1C: Human Hepatocellular Carcinoma- Hep 3B

|  | **24h** | | | | | |
| --- | --- | --- | --- | --- | --- | --- |
| **Concentration**  **(µM)** | **1** | **2** | **3** | **Mean** | **SD** | **SEM** |
| **7.813** | 97.67 | 99.02 | 94.41 | 97.03 | 2.36 | 1.36 |
| **15.625** | 100.12 | 100.26 | 93.93 | 98.10 | 3.61 | 2.09 |
| **31.25** | 90.28 | 95.90 | 90.89 | 92.36 | 3.08 | 1.78 |
| **62.5** | 68.32 | 70.716 | 67.96 | 69.00 | 1.49 | 0.86 |
| **125** | 35 | 39.0743 | 36.36 | 36.81 | 2.07 | 1.19 |
| **250** | 39 | 43.7917 | 36.16 | 39.65 | 3.85 | 2.22 |
| **500** | 49.73 | 45.08 | 41.62 | 45.47 | 4.06 | 2.35 |

|  | **48h** | | | | | |
| --- | --- | --- | --- | --- | --- | --- |
| **Concentration**  **(µM)** | **1** | **2** | **3** | **Mean** | **SD** | **SEM** |
| **7.813** | 98.38 | 107.64 | 104.94 | 103.65 | 4.76 | 2.75 |
| **15.625** | 95.03 | 105.16 | 101.17 | 100.46 | 5.10 | 2.94 |
| **31.25** | 94.38 | 98.99 | 103.83 | 99.073 | 4.72 | 2.73 |
| **62.5** | 86.49 | 55.69 | 74.64 | 72.27 | 15.53 | 8.97 |
| **125** | 46.78 | 31.52 | 37.09 | 38.46 | 7.72 | 4.46 |
| **250** | 42.88 | 40.46 | 41.93 | 41.76 | 1.21 | 0.70 |
| **500** | 40.00 | 39.02 | 41.28 | 40.10 | 1.132 | 0.65 |

|  | **72h** | | | | | |
| --- | --- | --- | --- | --- | --- | --- |
| **Concentration**  **(µM)** | **1** | **2** | **3** | **Mean** | **SD** | **SEM** |
| **7.813** | 109.50 | 109.40 | 108 | 108.97 | 0.84 | 0.48 |
| **15.625** | 92.28 | 116.28 | 100 | 102.85 | 12.25 | 7.08 |
| **31.25** | 102.13 | 108.05 | 101 | 103.72 | 3.78 | 2.18 |
| **62.5** | 64.33 | 60 | 65 | 63.11 | 2.71 | 1.56 |
| **125** | 57.61 | 58.08 | 59 | 58.23 | 0.70 | 0.40 |
| **250** | 54.15 | 54.38 | 54 | 54.17 | 0.19 | 0.11 |
| **500** | 55.97 | 51.40 | 55 | 54.12 | 2.40 | 1.39 |

Fig 1D: Human Colorectal Carcinoma- HCT 116

|  | **24h** | | | | | |
| --- | --- | --- | --- | --- | --- | --- |
| **Concentration**  **(µM)** | **1** | **2** | **3** | **Mean** | **SD** | **SEM** |
| **7.813** | 104.23 | 107.67 | 95.97 | 102.62 | 6.01 | 3.47 |
| **15.625** | 102.08 | 102.28 | 91.11 | 98.49 | 6.39 | 3.69 |
| **31.25** | 100.50 | 101.51 | 91.01 | 97.68 | 5.79 | 3.34 |
| **62.5** | 89.20 | 93.64 | 91.38 | 91.40 | 2.21 | 1.28 |
| **125** | 62.48 | 81.56 | 71.82 | 71.95 | 9.54 | 5.51 |
| **250** | 33.76 | 39.12 | 40.11 | 37.66 | 3.41 | 1.97 |
| **500** | 30.71 | 36.61 | 40.31 | 35.88 | 4.84 | 2.79 |

|  | **48h** | | | | | |
| --- | --- | --- | --- | --- | --- | --- |
| **Concentration**  **(µM)** | **1** | **2** | **3** | **Mean** | **SD** | **SEM** |
| **7.813** | 102.79 | 108.93 | 95.04 | 102.25 | 6.96 | 4.02 |
| **15.625** | 97.98 | 105.97 | 94.41 | 99.45 | 5.92 | 3.42 |
| **31.25** | 100.51 | 100.38 | 99.08 | 99.99 | 0.79 | 0.45 |
| **62.5** | 75.11 | 72.99 | 88.90 | 79.00 | 8.64 | 4.99 |
| **125** | 48.5 | 48.20 | 52.51 | 49.74 | 2.40 | 1.39 |
| **250** | 49.54 | 53.35 | 41.68 | 48.19 | 5.95 | 3.44 |
| **500** | 34.63 | 42.78 | 43.04 | 40.15 | 4.78 | 2.76 |

|  | **72h** | | | | | |
| --- | --- | --- | --- | --- | --- | --- |
| **Concentration**  **(µM)** | **1** | **2** | **3** | **Mean** | **SD** | **SEM** |
| **7.813** | 110.86 | 112.69 | 110.13 | 111.23 | 1.31 | 0.76 |
| **15.625** | 99.96 | 105.76 | 102.79 | 102.84 | 2.89 | 1.67 |
| **31.25** | 93.40 | 98.96 | 101.75 | 98.04 | 4.25 | 2.45 |
| **62.5** | 33.78 | 36.85 | 43.137 | 37.92 | 4.76 | 2.75 |
| **125** | 32.75 | 32.98 | 39.58 | 35.11 | 3.87 | 2.24 |
| **250** | 27.65 | 28.74 | 33.03 | 29.81 | 2.84 | 1.64 |
| **500** | 27.79 | 27.57 | 29.31 | 28.22 | 0.94 | 0.54 |

Fig 1E: Human Cervical Carcinoma- HeLa

|  | **24h** | | | | | |
| --- | --- | --- | --- | --- | --- | --- |
| **Concentration**  **(µM)** | **1** | **2** | **3** | **Mean** | **SD** | **SEM** |
| **7.813** | 105.02 | 102.18 | 103.30 | 103.50 | 1.42 | 0.82 |
| **15.625** | 108.51 | 102.65 | 104.32 | 105.16 | 3.01 | 1.74 |
| **31.25** | 102.81 | 103.11 | 105.76 | 103.90 | 1.62 | 0.93 |
| **62.5** | 84.43 | 86.27 | 101.61 | 90.77 | 9.43 | 5.45 |
| **125** | 41.46 | 22.67 | 52.24 | 38.79 | 14.96 | 8.6 |
| **250** | 23.29 | 27.04 | 13.65 | 21.33 | 6.90 | 3.99 |
| **500** | 21.66 | 41.97 | 17.30 | 26.97 | 13.16 | 7.61 |

|  | **48h** | | | | | |
| --- | --- | --- | --- | --- | --- | --- |
| **Concentration**  **(µM)** | **1** | **2** | **3** | **Mean** | **SD** | **SEM** |
| **7.813** | 110.65 | 110.65 | 110.05 | 110.45 | 0.34 | 0.20 |
| **15.625** | 112.93 | 107.10 | 111.02 | 110.35 | 2.97 | 1.71 |
| **31.25** | 107.19 | 75.68 | 107.54 | 96.80 | 18.29 | 10.57 |
| **62.5** | 45.18 | 19.65 | 55.23 | 40.02 | 18.33 | 10.60 |
| **125** | 19.26 | 35.41 | 4.35 | 19.67 | 15.53 | 8.98 |
| **250** | 16.34 | 40.27 | 14.44 | 23.68 | 14.39 | 8.31 |
| **500** | 16.09 | 32.36 | 14.34 | 20.93 | 9.93 | 5.74 |

|  | 72h | | | | |
| --- | --- | --- | --- | --- | --- |
| **Conc.** | **1** | **2** | **Mean** | **SD** | **SEM** |
| **7.813** | 105.28 | 123.89 | 114.59 | 13.16 | 9.33 |
| **15.625** | 102.58 | 115.19 | 108.89 | 8.92 | 6.32 |
| **31.25** | 93.46 | 112.60 | 103.03 | 13.53 | 9.60 |
| **62.5** | 6.63 | 28.30 | 17.46 | 15.32 | 10.87 |
| **125** | 9.48 | 6.70 | 8.09 | 1.97 | 1.40 |
| **250** | 17.84 | 11.95 | 14.90 | 4.17 | 2.95 |
| **500** | 26.46 | 11.57 | 19.01 | 10.52 | 7.46 |

Fig 1F: Human Glioblastoma- U-87 MG

|  | **24h** | | | | | |
| --- | --- | --- | --- | --- | --- | --- |
| **Concentration**  **(µM)** | **1** | **2** | **3** | **Mean** | **SD** | **SEM** |
| **7.813** | 93.5 | 96.6 | 93.7 | 94.60 | 1.73 | 1.00 |
| **15.625** | 93.1 | 97.7 | 95 | 95.27 | 2.31 | 1.34 |
| **31.25** | 92.9 | 90.4 | 90.9 | 91.40 | 1.32 | 0.76 |
| **62.5** | 90.5 | 90.7 | 88.3 | 89.83 | 1.33 | 0.77 |
| **125** | 75.8 | 77.6 | 77.9 | 77.10 | 1.14 | 0.66 |
| **250** | 51.5 | 45.5 | 39.7 | 45.57 | 5.90 | 3.41 |
| **500** | 17.9 | 17.2 | 9.2 | 14.77 | 4.83 | 2.79 |

|  | **48h** | | | | | |
| --- | --- | --- | --- | --- | --- | --- |
| **Concentration**  **(µM)** | **1** | **2** | **3** | **Mean** | **SD** | **SEM** |
| **7.813** | 106.7 | 95.9 | 105.6 | 102.73 | 5.94 | 3.44 |
| **15.625** | 105.4 | 97.3 | 103 | 101.90 | 4.16 | 2.40 |
| **31.25** | 102.9 | 93.8 | 97.2 | 97.97 | 4.60 | 2.66 |
| **62.5** | 95.4 | 83 | 84.9 | 87.77 | 6.68 | 3.86 |
| **125** | 45.7 | 40.1 | 32 | 39.27 | 6.89 | 3.98 |
| **250** | 12 | 5.7 | 9.1 | 8.93 | 3.15 | 1.82 |
| **500** | 9.1 | 9.1 | 12.7 | 10.30 | 2.08 | 1.20 |

|  | **72h** | | | | | |
| --- | --- | --- | --- | --- | --- | --- |
| **Concentration**  **(µM)** | **1** | **2** | **3** | **Mean** | **SD** | **SEM** |
| **7.813** | 92.7 | 96.6 | 102.22 | 97.17 | 4.79 | 2.77 |
| **15.625** | 94.3 | 101.1 | 112.93 | 102.78 | 9.43 | 5.45 |
| **31.25** | 95.6 | 99.2 | 108.85 | 101.22 | 6.85 | 3.96 |
| **62.5** | 93.4 | 98.3 | 106.17 | 99.29 | 6.44 | 3.72 |
| **125** | 14.7 | 12.5 | 3.71 | 10.30 | 5.81 | 3.36 |
| **250** | 10.1 | 13.4 | 8.82 | 10.77 | 2.36 | 1.37 |
| **500** | 14.9 | 19.1 | 18.92 | 17.64 | 2.37 | 1.37 |

Fig 1G: Rat Glioblastoma-C6

|  | **24h** | | | | | |
| --- | --- | --- | --- | --- | --- | --- |
| **Concentration**  **(µM)** | **1** | **2** | **3** | **Mean** | **SD** | **SEM** |
| **7.813** | 128.1 | 119.5 | 106.7 | 118.10 | 10.77 | 6.22 |
| **15.625** | 123.8 | 125.7 | 115.6 | 121.70 | 5.37 | 3.10 |
| **31.25** | 118.1 | 118.6 | 108.3 | 115.00 | 5.81 | 3.36 |
| **62.5** | 109.9 | 115.9 | 105.8 | 110.53 | 5.08 | 2.94 |
| **125** | 97.2 | 98.9 | 98 | 98.03 | 0.85 | 0.49 |
| **250** | 62.4 | 68.5 | 70.3 | 67.07 | 4.14 | 2.39 |
| **500** | 28.1 | 35.3 | 25 | 29.47 | 5.28 | 3.05 |

|  | **48h** | | | | | |
| --- | --- | --- | --- | --- | --- | --- |
| **Concentration**  **(µM)** | **1** | **2** | **3** | **Mean** | **SD** | **SEM** |
| **7.813** | 98.3 | 95.9 | 94.9 | 96.37 | 1.75 | 1.01 |
| **15.625** | 98 | 99.6 | 104.3 | 100.63 | 3.27 | 1.89 |
| **31.25** | 93.2 | 91.6 | 94.4 | 93.07 | 1.40 | 0.81 |
| **62.5** | 49.1 | 49.8 | 60.4 | 53.10 | 6.33 | 3.66 |
| **125** | 7.2 | 6.4 | 5.3 | 6.30 | 0.95 | 0.55 |
| **250** | 5.4 | 6 | 4.5 | 5.30 | 0.75 | 0.44 |
| **500** | 10.1 | 7.7 | 6 | 7.93 | 2.06 | 1.19 |

|  | **72h** | | | | | |
| --- | --- | --- | --- | --- | --- | --- |
| **Concentration**  **(µM)** | **1** | **2** | **3** | **Mean** | **SD** | **SEM** |
| **7.813** | 102.7 | 103.2 | 103.5 | 103.13 | 0.40 | 0.23 |
| **15.625** | 107.4 | 107.9 | 104.7 | 106.67 | 1.72 | 1.00 |
| **31.25** | 94.8 | 87.4 | 103.7 | 95.30 | 8.16 | 4.72 |
| **62.5** | 24.1 | 34.4 | 28.8 | 29.10 | 5.16 | 2.98 |
| **125** | 5.3 | 4.6 | 5.2 | 5.03 | 0.38 | 0.22 |
| **250** | 8.7 | 7.8 | 6.8 | 7.77 | 0.95 | 0.55 |
| **500** | 12.3 | 11.8 | 8.8 | 10.97 | 1.89 | 1.09 |

Fig 1H: Ehrlich Ascites Carcinoma (EAC)

|  | **24h** | | | | |
| --- | --- | --- | --- | --- | --- |
| **Concentration**  **(µM)** | **1** | **2** | **Mean** | **SD** | **SEM** |
| **7.813** | 103.0123 | 105.497 | 104.25 | 1.76 | 1.25 |
| **15.625** | 101.8629 | 98.73 | 100.30 | 2.22 | 1.57 |
| **31.25** | 98.73167 | 90.367 | 94.55 | 5.91 | 4.19 |
| **62.5** | 79.98415 | 70.726 | 75.36 | 6.55 | 4.64 |
| **125** | 87.99049 | 92.951 | 90.47 | 3.51 | 2.49 |
| **250** | 74.4352 | 70.248 | 72.34 | 2.96 | 2.10 |
| **500** | 68.41062 | 60.298 | 64.35 | 5.74 | 4.07 |

|  | **48h** | | | | |
| --- | --- | --- | --- | --- | --- |
| **Concentration**  **(µM)** | **1** | **2** | **Mean** | **SD** | **SEM** |
| **7.813** | 118.83 | 120.85 | 119.84 | 1.43 | 1.01 |
| **15.625** | 99.041 | 112.34 | 105.69 | 9.40 | 6.67 |
| **31.25** | 87.708 | 85 | 86.35 | 1.92 | 1.36 |
| **62.5** | 78.916 | 83.96 | 81.44 | 3.57 | 2.53 |
| **125** | 66.458 | 60.58 | 63.52 | 4.15 | 2.94 |
| **250** | 44.625 | 40.74 | 42.68 | 2.75 | 1.95 |
| **500** | 39.66 | 32.93 | 36.30 | 4.76 | 3.38 |

**Fig 3A:** **AO/EtBr staining of Hepatocellular carcinoma cell line-Hep 3B**

**24h**

|  | **Vitamin D alone 24 h** | | | |
| --- | --- | --- | --- | --- |
|  | Control (Untreated) | | | |
|  | **Set 1** | **Set 2** | **Set 3** | **Set 4** |
| Total Cells | 140 | 154 | 189 | 205 |
| Live Cells | 131 | 144 | 184 | 196 |
| Dead Cells | 9 | 10 | 5 | 9 |
| % Dead Cells | 6.428 | 6.49 | 2.64 | 4.39 |
| **Mean** | 4.99 | | | |
| **SD** | 1.84 | | | |
| **SEM** | 0.92 | | | |

|  | **Vitamin D alone 24 h** | | | |
| --- | --- | --- | --- | --- |
|  | Vehicle Control (VC) | | | |
|  | **Set 1** | **Set 2** | **Set 3** | **Set 4** |
| Total Cells | 135 | 137 | 233 | 330 |
| Live Cells | 121 | 124 | 198 | 295 |
| Dead Cells | 14 | 13 | 35 | 35 |
| % Dead Cells | 10.37 | 9.48 | 15.02 | 10.61 |
| **Mean** | 11.37 | | | |
| **SD** | 2.48 | | | |
| **SEM** | 1.24 | | | |

|  | **Vitamin D alone 24 h** | | | |
| --- | --- | --- | --- | --- |
|  | Positive Control (PC) | | | |
|  | **Set 1** | **Set 2** | **Set 3** | **Set 4** |
| Total Cells | 210 | 225 | 212 | 79 |
| Live Cells | 34 | 30 | 32 | 14 |
| Dead Cells | 176 | 195 | 180 | 65 |
| % Dead Cells | 83.81 | 86.67 | 84.91 | 82.27848 |
| **Mean** | 84.42 | | | |
| **SD** | 1.85 | | | |
| **SEM** | 0.92 | | | |

|  | **Vitamin D alone 24 h** | | | |
| --- | --- | --- | --- | --- |
|  | Vit D3 62.5µM | | | |
|  | **Set 1** | **Set 2** | **Set 3** | **Set 4** |
| Total Cells | 292 | 203 | 247 | 159 |
| Live Cells | 248 | 164 | 216 | 137 |
| Dead Cells | 44 | 39 | 31 | 22 |
| % Dead Cells | 15.07 | 19.21 | 12.55 | 13.84 |
| **Mean** | 15.17 | | | |
| **SD** | 2.89 | | | |
| **SEM** | 1.44 | | | |

|  | **Vitamin D alone 24 h** | | | |
| --- | --- | --- | --- | --- |
|  | Vit D3 125 µM | | | |
|  | **Set 1** | **Set 2** | **Set 3** | **Set 4** |
| Total Cells | 229 | 255 | 352 | 341 |
| Live Cells | 171 | 183 | 238 | 259 |
| Dead Cells | 58 | 72 | 114 | 82 |
| % Dead Cells | 25.33 | 28.24 | 32.39 | 24.05 |
| **Mean** | 27.50 | | | |
| **SD** | 3.70 | | | |
| **SEM** | 1.85 | | | |

|  | **Vitamin D alone 24h** | | | |
| --- | --- | --- | --- | --- |
|  | Vit D3 250 µM | | | |
|  | **Set 1** | **Set 2** | **Set 3** | **Set 4** |
| Total Cells | 161 | 268 | 206 | 223 |
| Live Cells | 52 | 63 | 50 | 40 |
| Dead Cells | 109 | 205 | 156 | 183 |
| % Dead Cells | 67.70 | 76.49 | 75.73 | 82.06 |
| **Mean** | 75.50 | | | |
| **SD** | 5.91 | | | |
| **SEM** | 2.96 | | | |

**48h**

|  | **Vitamin D alone 48 h** | | | |
| --- | --- | --- | --- | --- |
|  | Control (Untreated) | | | |
|  | **Set 1** | **Set 2** | **Set 3** | **Set 4** |
| Total Cells | 219 | 226 | 286 | 267 |
| Live Cells | 188 | 195 | 248 | 226 |
| Dead Cells | 31 | 31 | 38 | 41 |
| % Dead Cells | 14.2 | 13.72 | 13.29 | 15.36 |
| **Mean** | 14.13 | | | |
| **SD** | 0.89 | | | |
| **SEM** | 0.45 | | | |

|  | **Vitamin D alone 48 h** | | | |
| --- | --- | --- | --- | --- |
|  | Vehicle Control (VC) | | | |
|  | **Set 1** | **Set 2** | **Set 3** | **Set 4** |
| Total Cells | 250 | 447 | 348 | 341 |
| Live Cells | 227 | 399 | 293 | 289 |
| Dead Cells | 23 | 48 | 55 | 51 |
| % Dead Cells | 9.20 | 10.74 | 15.80 | 14.96 |
| **Mean** | 12.67 | | | |
| **SD** | 3.21 | | | |
| **SEM** | 1.60 | | | |

|  | **Vitamin D alone 48 h** | | | |
| --- | --- | --- | --- | --- |
|  | Positive Control (PC) | | | |
|  | **Set 1** | **Set 2** | **Set 3** | **Set 4** |
| Total Cells | 152 | 210 | 226 | 220 |
| Live Cells | 9 | 34 | 29 | 33 |
| Dead Cells | 143 | 176 | 197 | 192 |
| % Dead Cells | 94.08 | 83.81 | 87.17 | 87.27 |
| **Mean** | 88.08 | | | |
| **SD** | 4.31 | | | |
| **SEM** | 2.15 | | | |

|  | **Vitamin D alone 48 h** | | | |
| --- | --- | --- | --- | --- |
|  | Vit D3 31.25µM | | | |
|  | **Set 1** | **Set 2** | **Set 3** | **Set 4** |
| Total Cells | 292 | 135 | 241 | 186 |
| Live Cells | 248 | 121 | 207 | 153 |
| Dead Cells | 44 | 14 | 34 | 33 |
| % Dead Cells | 15.06 | 10.37 | 14.10 | 17.74 |
| **Mean** | 14.32 | | | |
| **SD** | 3.05 | | | |
| **SEM** | 1.53 | | | |

|  | **Vitamin D alone 48 h** | | | |
| --- | --- | --- | --- | --- |
|  | Vit D3 62.5 µM | | | |
|  | **Set 1** | **Set 2** | **Set 3** | **Set 4** |
| Total Cells | 283 | 229 | 183 | 210 |
| Live Cells | 220 | 171 | 135 | 157 |
| Dead Cells | 63 | 58 | 48 | 53 |
| % Dead Cells | 22.26 | 25.32 | 26.22 | 25.23 |
| **Mean** | 24.76 | | | |
| **SD** | 1.73 | | | |
| **SEM** | 0.86 | | | |

|  | **Vitamin D alone 24h** | | | |
| --- | --- | --- | --- | --- |
|  | VitD3 125 µM | | | |
|  | **Set 1** | **Set 2** | **Set 3** | **Set 4** |
| Total Cells | 352 | 288 | 125 | 119 |
| Live Cells | 238 | 191 | 86 | 87 |
| Dead Cells | 114 | 97 | 39 | 32 |
| % Dead Cells | 32.38 | 33.68 | 45.34 | 36.78 |
| **Mean** | 37.05 | | | |
| **SD** | 5.83 | | | |
| **SEM** | 2.92 | | | |

**Fig 3B**: **AO/EtBr staining of Colorectal carcinoma cell line-HCT 116**

**24h**

|  | **Vitamin D alone 24 h** | | | |
| --- | --- | --- | --- | --- |
|  | Control (Untreated) | | | |
|  | **Set 1** | **Set 2** | **Set 3** | **Set 4** |
| Total Cells | 308 | 316 | 124 | 117 |
| Live Cells | 290 | 299 | 117 | 113 |
| Dead Cells | 18 | 17 | 7 | 4 |
| % Dead Cells | 5.84 | 5.37 | 5.64 | 3.41 |
| **Mean** | 5.07 | | | |
| **SD** | 1.12 | | | |
| **SEM** | 0.56 | | | |

|  | **Vitamin D alone 24 h** | | | |
| --- | --- | --- | --- | --- |
|  | Vehicle Control (VC) | | | |
|  | **Set 1** | **Set 2** | **Set 3** | **Set 4** |
| Total Cells | 149 | 259 | 174 | 107 |
| Live Cells | 141 | 250 | 165 | 101 |
| Dead Cells | 8 | 9 | 9 | 6 |
| % Dead Cells | 5.36 | 3.47 | 5.17 | 5.60 |
| **Mean** | 4.91 | | | |
| **SD** | 0.97 | | | |
| **SEM** | 0.49 | | | |

|  | **Vitamin D alone 24 h** | | | |
| --- | --- | --- | --- | --- |
|  | Positive Control (PC) | | | |
|  | **Set 1** | **Set 2** | **Set 3** | **Set 4** |
| Total Cells | 197 | 147 | 174 | 192 |
| Live Cells | 105 | 87 | 104 | 109 |
| Dead Cells | 92 | 60 | 70 | 83 |
| % Dead Cells | 46.70 | 40.81 | 40.22 | 43.22 |
| **Mean** | 42.74 | | | |
| **SD** | 2.94 | | | |
| **SEM** | 1.47 | | | |

|  | **Vitamin D alone 24 h** | | | |
| --- | --- | --- | --- | --- |
|  | Vit D3 31.25µM | | | |
|  | **Set 1** | **Set 2** | **Set 3** | **Set 4** |
| Total Cells | 110 | 264 | 139 | 114 |
| Live Cells | 105 | 244 | 127 | 105 |
| Dead Cells | 5 | 20 | 12 | 9 |
| % Dead Cells | 4.54 | 7.57 | 8.63 | 7.89 |
| **Mean** | 7.16 | | | |
| **SD** | 1.80 | | | |
| **SEM** | 0.90 | | | |

|  | **Vitamin D alone 24 h** | | | |
| --- | --- | --- | --- | --- |
|  | Vit D3 62.5µM | | | |
|  | **Set 1** | **Set 2** | **Set 3** | **Set 4** |
| Total Cells | 145 | 202 | 438 | 210 |
| Live Cells | 123 | 164 | 392 | 185 |
| Dead Cells | 22 | 38 | 46 | 25 |
| % Dead Cells | 15.17 | 18.81 | 10.50 | 11.90 |
| **Mean** | 14.10 | | | |
| **SD** | 3.70 | | | |
| **SEM** | 1.85 | | | |

|  | **Vitamin D alone 24h** | | | |
| --- | --- | --- | --- | --- |
|  | Vit D3 125µM | | | |
|  | **Set 1** | **Set 2** | **Set 3** | **Set 4** |
| Total Cells | 176 | 214 | 186 | 181 |
| Live Cells | 97 | 156 | 129 | 115 |
| Dead Cells | 79 | 58 | 57 | 66 |
| % Dead Cells | 44.88 | 27.10 | 30.64 | 36.46 |
| **Mean** | 34.77 | | | |
| **SD** | 7.77 | | | |
| **SEM** | 3.88 | | | |

**48h**

|  | **Vitamin D alone 48 h** | | | |
| --- | --- | --- | --- | --- |
|  | Control (Untreated) | | | |
|  | **Set 1** | **Set 2** | **Set 3** | **Set 4** |
| Total Cells | 271 | 339 | 342 | 296 |
| Live Cells | 237 | 299 | 297 | 267 |
| Dead Cells | 34 | 40 | 45 | 29 |
| % Dead Cells | 12.54 | 11.79 | 13.15 | 9.79 |
| **Mean** | 11.83 | | | |
| **SD** | 1.46 | | | |
| **SEM** | 0.73 | | | |

|  | **Vitamin D alone 48 h** | | | |
| --- | --- | --- | --- | --- |
|  | Vehicle Control (VC) | | | |
|  | **Set 1** | **Set 2** | **Set 3** | **Set 4** |
| Total Cells | 262 | 264 | 337 | 239 |
| Live Cells | 234 | 244 | 297 | 218 |
| Dead Cells | 28 | 20 | 40 | 21 |
| % Dead Cells | 10.68 | 7.57 | 11.86 | 8.78 |
| **Mean** | 9.73 | | | |
| **SD** | 1.92 | | | |
| **SEM** | 0.96 | | | |

|  | **Vitamin D alone 48 h** | | | |
| --- | --- | --- | --- | --- |
|  | Positive Control (PC) | | | |
|  | **Set 1** | **Set 2** | **Set 3** | **Set 4** |
| Total Cells | 74 | 73 | 91 | 73 |
| Live Cells | 8 | 4 | 3 | 5 |
| Dead Cells | 66 | 69 | 88 | 68 |
| % Dead Cells | 89.18 | 94.52 | 96.70 | 93.15 |
| **Mean** | 93.39 | | | |
| **SD** | 3.16 | | | |
| **SEM** | 1.58 | | | |

|  | **Vitamin D alone 48 h** | | | |
| --- | --- | --- | --- | --- |
|  | Vit D3 31.25µM | | | |
|  | **Set 1** | **Set 2** | **Set 3** | **Set 4** |
| Total Cells | 402 | 306 | 383 | 438 |
| Live Cells | 347 | 266 | 308 | 337 |
| Dead Cells | 55 | 40 | 75 | 101 |
| % Dead Cells | 13.68 | 13.07 | 19.58 | 23.05 |
| **Mean** | 17.35 | | | |
| **SD** | 4.81 | | | |
| **SEM** | 2.40 | | | |

|  | **Vitamin D alone 48 h** | | | |
| --- | --- | --- | --- | --- |
|  | Vit D3 62.5µM | | | |
|  | **Set 1** | **Set 2** | **Set 3** | **Set 4** |
| Total Cells | 179 | 170 | 185 | 202 |
| Live Cells | 146 | 152 | 150 | 162 |
| Dead Cells | 33 | 18 | 35 | 40 |
| % Dead Cells | 18.43 | 10.58 | 18.91 | 19.80 |
| **Mean** | 16.94 | | | |
| **SD** | 4.27 | | | |
| **SEM** | 2.13 | | | |

|  | **Vitamin D alone 48h** | | | |
| --- | --- | --- | --- | --- |
|  | Vit D3 125µM | | | |
|  | **Set 1** | **Set 2** | **Set 3** | **Set 4** |
| Total Cells | 138 | 95 | 163 | 92 |
| Live Cells | 98 | 57 | 101 | 55 |
| Dead Cells | 40 | 38 | 62 | 37 |
| % Dead Cells | 28.98 | 40 | 38.03 | 40.21 |
| **Mean** | 36.81 | | | |
| **SD** | 5.31 | | | |
| **SEM** | 2.65 | | | |

**Fig 3C:** **AO/EtBr staining of Cervical carcinoma cell line-HeLa**

**24h**

|  | **Vitamin D alone 24 h** | | | |
| --- | --- | --- | --- | --- |
|  | Control (Untreated) | | | |
|  | **Set 1** | **Set 2** | **Set 3** | **Set 4** |
| Total Cells | 253 | 237 | 214 | 204 |
| Live Cells | 241 | 227 | 200 | 190 |
| Dead Cells | 12 | 10 | 14 | 14 |
| % Dead Cells | 4.74 | 4.22 | 6.54 | 6.86 |
| **Mean** | 5.59 | | | |
| **SD** | 1.31 | | | |
| **SEM** | 0.65 | | | |

|  | **Vitamin D alone 24 h** | | | |
| --- | --- | --- | --- | --- |
|  | Vehicle Control (VC) | | | |
|  | **Set 1** | **Set 2** | **Set 3** | **Set 4** |
| Total Cells | 316 | 352 | 339 | 326 |
| Live Cells | 291 | 326 | 316 | 300 |
| Dead Cells | 25 | 26 | 23 | 26 |
| % Dead Cells | 7.91 | 7.39 | 6.78 | 7.98 |
| **Mean** | 7.51 | | | |
| **SD** | 0.55 | | | |
| **SEM** | 0.28 | | | |

|  | **Vitamin D alone 24 h** | | | |
| --- | --- | --- | --- | --- |
|  | Positive Control (PC) | | | |
|  | **Set 1** | **Set 2** | **Set 3** | **Set 4** |
| Total Cells | 174 | 154 | 172 | 180 |
| Live Cells | 37 | 14 | 34 | 42 |
| Dead Cells | 137 | 140 | 138 | 138 |
| % Dead Cells | 78.74 | 90.91 | 80.23 | 76.67 |
| **Mean** | 81.64 | | | |
| **SD** | 6.35 | | | |
| **SEM** | 3.18 | | | |

|  | **Vitamin D alone 24 h** | | | |
| --- | --- | --- | --- | --- |
|  | Vit D3 62.5µM | | | |
|  | **Set 1** | **Set 2** | **Set 3** | **Set 4** |
| Total Cells | 330 | 353 | 406 | 351 |
| Live Cells | 300 | 320 | 376 | 321 |
| Dead Cells | 30 | 33 | 30 | 30 |
| % Dead Cells | 9.09 | 9.35 | 7.39 | 8.55 |
| **Mean** | 8.59 | | | |
| **SD** | 0.87 | | | |
| **SEM** | 0.43 | | | |

|  | **Vitamin D alone 24 h** | | | |
| --- | --- | --- | --- | --- |
|  | Vit D3 125µM | | | |
|  | **Set 1** | **Set 2** | **Set 3** | **Set 4** |
| Total Cells | 199 | 197 | 195 | 172 |
| Live Cells | 140 | 135 | 144 | 122 |
| Dead Cells | 59 | 62 | 51 | 50 |
| % Dead Cells | 29.65 | 31.47 | 26.15 | 29.07 |
| **Mean** | 29.09 | | | |
| **SD** | 2.21 | | | |
| **SEM** | 1.10 | | | |

|  | **Vitamin D alone 24 h** | | | |
| --- | --- | --- | --- | --- |
|  | Vit D3 250µM | | | |
|  | **Set 1** | **Set 2** | **Set 3** | **Set 4** |
| Total Cells | 124 | 123 | 111 | 119 |
| Live Cells | 20 | 29 | 22 | 26 |
| Dead Cells | 104 | 94 | 89 | 93 |
| % Dead Cells | 83.87 | 76.42 | 80.18 | 78.15 |
| **Mean** | 79.66 | | | |
| **SD** | 3.20 | | | |
| **SEM** | 1.60 | | | |

**48h**

|  | **Vitamin D alone 48 h** | | | |
| --- | --- | --- | --- | --- |
|  | Control (Untreated) | | | |
|  | **Set 1** | **Set 2** | **Set 3** | **Set 4** |
| Total Cells | 209 | 170 | 263 | 259 |
| Live Cells | 196 | 155 | 251 | 249 |
| Dead Cells | 13 | 15 | 12 | 10 |
| % Dead Cells | 6.22 | 8.82 | 4.56 | 3.86 |
| **Mean** | 5.87 | | | |
| **SD** | 2.21 | | | |
| **SEM** | 1.10 | | | |

|  | **Vitamin D alone 48 h** | | | |
| --- | --- | --- | --- | --- |
|  | Vehicle Control (VC) | | | |
|  | **Set 1** | **Set 2** | **Set 3** | **Set 4** |
| Total Cells | 309 | 329 | 300 | 287 |
| Live Cells | 293 | 309 | 281 | 272 |
| Dead Cells | 16 | 20 | 19 | 15 |
| % Dead Cells | 5.18 | 6.08 | 6.33 | 5.23 |
| **Mean** | 5.70 | | | |
| **SD** | 0.59 | | | |
| **SEM** | 0.29 | | | |

|  | **Vitamin D alone 48 h** | | | |
| --- | --- | --- | --- | --- |
|  | Positive Control (PC) | | | |
|  | **Set 1** | **Set 2** | **Set 3** | **Set 4** |
| Total Cells | 85 | 96 | 104 | 97 |
| Live Cells | 5 | 7 | 10 | 8 |
| Dead Cells | 80 | 89 | 94 | 89 |
| % Dead Cells | 94.11765 | 92.70833 | 90.38462 | 91.75258 |
| **Mean** | 92.24 | | | |
| **SD** | 1.57 | | | |
| **SEM** | 0.79 | | | |

|  | **Vitamin D alone 48 h** | | | |
| --- | --- | --- | --- | --- |
|  | Vit D3 62.5µM | | | |
|  | **Set 1** | **Set 2** | **Set 3** | **Set 4** |
| Total Cells | 200 | 205 | 264 | 248 |
| Live Cells | 131 | 143 | 186 | 180 |
| Dead Cells | 69 | 62 | 78 | 68 |
| % Dead Cells | 34.5 | 30.2439 | 29.54545 | 27.41935 |
| **Mean** | 30.43 | | | |
| **SD** | 2.97 | | | |
| **SEM** | 1.48 | | | |

|  | **Vitamin D alone 48 h** | | | |
| --- | --- | --- | --- | --- |
|  | Vit D3 125µM | | | |
|  | **Set 1** | **Set 2** | **Set 3** | **Set 4** |
| Total Cells | 142 | 139 | 141 | 137 |
| Live Cells | 33 | 29 | 34 | 32 |
| Dead Cells | 109 | 110 | 107 | 105 |
| % Dead Cells | 76.76056338 | 79.136691 | 75.886525 | 76.642336 |
| **Mean** | 77.11 | | | |
| **SD** | 1.41 | | | |
| **SEM** | 0.70 | | | |

|  | **Vitamin D alone 48h** | | | |
| --- | --- | --- | --- | --- |
|  | Vit D3 250 µM | | | |
|  | **Set 1** | **Set 2** | **Set 3** | **Set 4** |
| Total Cells | 76 | 88 | 88 | 83 |
| Live Cells | 10 | 8 | 9 | 5 |
| Dead Cells | 66 | 80 | 79 | 78 |
| % Dead Cells | 86.84211 | 90.90909 | 89.77273 | 93.9759 |
| **Mean** | 90.37 | | | |
| **SD** | 2.95 | | | |
| **SEM** | 1.47 | | | |

**Fig 3D:** **AO/EtBr staining of Human glioblastoma cell line-U-87 MG**

**24h**

|  | **Vitamin D alone 24 h** | | | |
| --- | --- | --- | --- | --- |
|  | Control (Untreated) | | | |
|  | **Set 1** | **Set 2** | **Set 3** | **Set 4** |
| Total Cells | 324 | 357 | 358 | 524 |
| Live Cells | 277 | 326 | 313 | 460 |
| Dead Cells | 47 | 31 | 45 | 64 |
| % Dead Cells | 14.51 | 8.68 | 12.57 | 12.21 |
| **Mean** | 11.99 | | | |
| **SD** | 2.43 | | | |
| **SEM** | 1.21 | | | |

|  | **Vitamin D alone 24 h** | | | |
| --- | --- | --- | --- | --- |
|  | Vehicle Control (VC) | | | |
|  | **Set 1** | **Set 2** | **Set 3** | **Set 4** |
| Total Cells | 311 | 389 | 428 | 431 |
| Live Cells | 279 | 346 | 378 | 389 |
| Dead Cells | 32 | 43 | 50 | 42 |
| % Dead Cells | 10.29 | 11.05 | 11.68 | 9.74 |
| **Mean** | 10.69 | | | |
| **SD** | 0.85 | | | |
| **SEM** | 0.43 | | | |

|  | **Vitamin D alone 24 h** | | | |
| --- | --- | --- | --- | --- |
|  | Positive Control (PC) | | | |
|  | **Set 1** | **Set 2** | **Set 3** | **Set 4** |
| Total Cells | 297 | 332 | 303 | 226 |
| Live Cells | 164 | 173 | 167 | 115 |
| Dead Cells | 133 | 159 | 136 | 111 |
| % Dead Cells | 44.78 | 47.89 | 44.88 | 49.12 |
| **Mean** | 46.67 | | | |
| **SD** | 2.18 | | | |
| **SEM** | 1.09 | | | |

|  | **Vitamin D alone 24 h** | | | |
| --- | --- | --- | --- | --- |
|  | Vit D3 62.5µM | | | |
|  | **Set 1** | **Set 2** | **Set 3** | **Set 4** |
| Total Cells | 478 | 518 | 320 | 335 |
| Live Cells | 391 | 443 | 259 | 274 |
| Dead Cells | 87 | 75 | 61 | 61 |
| % Dead Cells | 18.20 | 14.48 | 19.06 | 18.21 |
| **Mean** | 17.49 | | | |
| **SD** | 2.05 | | | |
| **SEM** | 1.02 | | | |

|  | **Vitamin D alone 24 h** | | | |
| --- | --- | --- | --- | --- |
|  | Vit D3 125µM | | | |
|  | **Set 1** | **Set 2** | **Set 3** | **Set 4** |
| Total Cells | 244 | 327 | 299 | 260 |
| Live Cells | 176 | 214 | 201 | 176 |
| Dead Cells | 68 | 113 | 98 | 84 |
| % Dead Cells | 27.87 | 34.56 | 32.78 | 32.31 |
| **Mean** | 31.88 | | | |
| **SD** | 2.84 | | | |
| **SEM** | 1.42 | | | |

|  | **Vitamin D alone 24 h** | | | |
| --- | --- | --- | --- | --- |
|  | Vit D3 250µM | | | |
|  | **Set 1** | **Set 2** | **Set 3** | **Set 4** |
| Total Cells | 329 | 384 | 385 | 326 |
| Live Cells | 151 | 157 | 154 | 160 |
| Dead Cells | 178 | 227 | 231 | 166 |
| % Dead Cells | 54.10 | 59.11 | 60.00 | 50.92 |
| **Mean** | 56.03 | | | |
| **SD** | 4.29 | | | |
| **SEM** | 2.14 | | | |

**48h**

|  | **Vitamin D alone 48 h** | | | |
| --- | --- | --- | --- | --- |
|  | Control (Untreated) | | | |
|  | **Set 1** | **Set 2** | **Set 3** | **Set 4** |
| Total Cells | 247 | 228 | 229 | 268 |
| Live Cells | 217 | 197 | 201 | 228 |
| Dead Cells | 30 | 31 | 28 | 40 |
| % Dead Cells | 12.15 | 13.60 | 12.23 | 14.93 |
| **Mean** | 13.22 | | | |
| **SD** | 1.32 | | | |
| **SEM** | 0.66 | | | |

|  | **Vitamin D alone 48h** | | | |
| --- | --- | --- | --- | --- |
|  | Vehicle Control (VC) | | | |
|  | **Set 1** | **Set 2** | **Set 3** | **Set 4** |
| Total Cells | 389 | 434 | 342 | 255 |
| Live Cells | 328 | 372 | 296 | 218 |
| Dead Cells | 61 | 62 | 46 | 37 |
| % Dead Cells | 15.68 | 14.29 | 13.45 | 14.51 |
| **Mean** | 14.48 | | | |
| **SD** | 0.92 | | | |
| **SEM** | 0.46 | | | |

|  | **Vitamin D alone 48 h** | | | |
| --- | --- | --- | --- | --- |
|  | Positive Control (PC) | | | |
|  | **Set 1** | **Set 2** | **Set 3** | **Set 4** |
| Total Cells | 432 | 225 | 325 | 378 |
| Live Cells | 54 | 32 | 21 | 55 |
| Dead Cells | 378 | 193 | 304 | 323 |
| % Dead Cells | 87.50 | 85.78 | 93.54 | 85.45 |
| **Mean** | 88.07 | | | |
| **SD** | 3.76 | | | |
| **SEM** | 1.88 | | | |

|  | **Vitamin D alone 48 h** | | | |
| --- | --- | --- | --- | --- |
|  | Vit D3 31.25µM | | | |
|  | **Set 1** | **Set 2** | **Set 3** | **Set 4** |
| Total Cells | 258 | 239 | 230 | 274 |
| Live Cells | 217 | 193 | 197 | 230 |
| Dead Cells | 41 | 46 | 33 | 44 |
| % Dead Cells | 15.89 | 19.25 | 14.35 | 16.06 |
| **Mean** | 16.39 | | | |
| **SD** | 2.06 | | | |
| **SEM** | 1.03 | | | |

|  | **Vitamin D alone 48 h** | | | |
| --- | --- | --- | --- | --- |
|  | Vit D3 62.5µM | | | |
|  | **Set 1** | **Set 2** | **Set 3** | **Set 4** |
| Total Cells | 370 | 405 | 455 | 316 |
| Live Cells | 287 | 295 | 328 | 231 |
| Dead Cells | 83 | 110 | 127 | 85 |
| % Dead Cells | 22.43 | 27.16 | 27.91 | 26.90 |
| **Mean** | 26.10 | | | |
| **SD** | 2.48 | | | |
| **SEM** | 1.24 | | | |

|  | **Vitamin D alone 48 h** | | | |
| --- | --- | --- | --- | --- |
|  | Vit D3 125µM | | | |
|  | **Set 1** | **Set 2** | **Set 3** | **Set 4** |
| Total Cells | 454 | 372 | 456 | 435 |
| Live Cells | 178 | 150 | 182 | 174 |
| Dead Cells | 276 | 222 | 274 | 261 |
| % Dead Cells | 60.79 | 59.68 | 60.09 | 60.00 |
| **Mean** | 60.14 | | | |
| **SD** | 0.47 | | | |
| **SEM** | 0.23 | | | |

**Fig 3E:** **AO/EtBr staining of Rat glioblastoma cell line-C6**

**24h**

|  | **Vitamin D alone 24 h** | | | |
| --- | --- | --- | --- | --- |
|  | Control(Untreated) | | | |
|  | **Set 1** | **Set 2** | **Set 3** | **Set 4** |
| Total Cells | 156 | 115 | 92 | 75 |
| Live Cells | 148 | 110 | 90 | 70 |
| Dead Cells | 8 | 5 | 2 | 5 |
| % Dead Cells | 5.13 | 4.35 | 2.17 | 6.67 |
| **Mean** | 4.58 | | | |
| **SD** | 1.87 | | | |
| **SEM** | 0.94 | | | |

|  | **Vitamin D alone 24 h** | | | |
| --- | --- | --- | --- | --- |
|  | Vehicle Control(VC) | | | |
|  | **Set 1** | **Set 2** | **Set 3** | **Set 4** |
| Total Cells | 182 | 114 | 110 | 198 |
| Live Cells | 168 | 105 | 101 | 173 |
| Dead Cells | 14 | 9 | 9 | 25 |
| % Dead Cells | 7.69 | 7.89 | 8.18 | 12.63 |
| **Mean** | 9.10 | | | |
| **SD** | 2.36 | | | |
| **SEM** | 1.18 | | | |

|  | **Vitamin D alone 24 h** | | | |
| --- | --- | --- | --- | --- |
|  | Positive Control (PC) | | | |
|  | **Set 1** | **Set 2** | **Set 3** | **Set 4** |
| Total Cells | 298 | 254 | 233 | 338 |
| Live Cells | 147 | 127 | 103 | 160 |
| Dead Cells | 151 | 127 | 130 | 178 |
| % Dead Cells | 50.67 | 50.00 | 55.79 | 52.66 |
| **Mean** | 52.28 | | | |
| **SD** | 2.60 | | | |
| **SEM** | 1.30 | | | |

|  | **Vitamin D alone 24 h** | | | |
| --- | --- | --- | --- | --- |
|  | Vit D3 125µM | | | |
|  | **Set 1** | **Set 2** | **Set 3** | **Set 4** |
| Total Cells | 310 | 369 | 344 | 376 |
| Live Cells | 276 | 337 | 326 | 352 |
| Dead Cells | 34 | 32 | 18 | 24 |
| % Dead Cells | 10.97 | 8.67 | 5.23 | 6.38 |
| **Mean** | 7.81 | | | |
| **SD** | 2.54 | | | |
| **SEM** | 1.27 | | | |

|  | **Vitamin D alone 24 h** | | | |
| --- | --- | --- | --- | --- |
|  | Vit D3 250µM | | | |
|  | **Set 1** | **Set 2** | **Set 3** | **Set 4** |
| Total Cells | 120 | 168 | 188 | 131 |
| Live Cells | 80 | 123 | 120 | 93 |
| Dead Cells | 40 | 45 | 68 | 38 |
| % Dead Cells | 33.33 | 26.79 | 36.17 | 29.01 |
| **Mean** | 31.32 | | | |
| **SD** | 4.22 | | | |
| **SEM** | 2.11 | | | |

|  | **Vitamin D alone 24 h** | | | |
| --- | --- | --- | --- | --- |
|  | Vit D3 500µM | | | |
|  | **Set 1** | **Set 2** | **Set 3** | **Set 4** |
| Total Cells | 218 | 143 | 117 | 66 |
| Live Cells | 88 | 35 | 23 | 14 |
| Dead Cells | 130 | 108 | 94 | 52 |
| % Dead Cells | 59.63 | 75.52 | 80.34 | 78.79 |
| **Mean** | 73.57 | | | |
| **SD** | 9.51 | | | |
| **SEM** | 4.75 | | | |

**48h**

|  | **Vitamin D alone 48 h** | | | |
| --- | --- | --- | --- | --- |
|  | Control | | | |
|  | **Set 1** | **Set 2** | **Set 3** | **Set 4** |
| Total Cells | 379 | 477 | 471 | 385 |
| Live Cells | 331 | 447 | 429 | 369 |
| Dead Cells | 48 | 30 | 42 | 16 |
| % Dead Cells | 12.66 | 6.29 | 8.92 | 4.16 |
| **Mean** | 8.01 | | | |
| **SD** | 3.67 | | | |
| **SEM** | 1.83 | | | |

|  | **Vitamin D alone 48h** | | | |
| --- | --- | --- | --- | --- |
|  | Vehicle Control(VC) | | | |
|  | **Set 1** | **Set 2** | **Set 3** | **Set 4** |
| Total Cells | 388 | 344 | 351 | 402 |
| Live Cells | 358 | 316 | 322 | 362 |
| Dead Cells | 30 | 28 | 29 | 40 |
| % Dead Cells | 7.73 | 8.14 | 8.26 | 9.95 |
| **Mean** | 8.52 | | | |
| **SD** | 0.98 | | | |
| **SEM** | 0.49 | | | |

|  | **Vitamin D alone 48 h** | | | |
| --- | --- | --- | --- | --- |
|  | Positive Control (PC) | | | |
|  | **Set 1** | **Set 2** | **Set 3** | **Set 4** |
| Total Cells | 83 | 100 | 102 | 145 |
| Live Cells | 18 | 14 | 19 | 23 |
| Dead Cells | 65 | 86 | 83 | 122 |
| % Dead Cells | 78.31 | 86.00 | 81.37 | 84.14 |
| **Mean** | 82.46 | | | |
| **SD** | 3.35 | | | |
| **SEM** | 1.68 | | | |

|  | **Vitamin D alone 48 h** | | | |
| --- | --- | --- | --- | --- |
|  | Vit D3 15.625µM | | | |
|  | **Set 1** | **Set 2** | **Set 3** | **Set 4** |
| Total Cells | 166 | 279 | 191 | 80 |
| Live Cells | 145 | 246 | 169 | 71 |
| Dead Cells | 21 | 33 | 22 | 9 |
| % Dead Cells | 12.65 | 11.83 | 11.52 | 11.25 |
| **Mean** | 11.81 | | | |
| **SD** | 0.61 | | | |
| **SEM** | 0.30 | | | |

|  | **Vitamin D alone 48 h** | | | |
| --- | --- | --- | --- | --- |
|  | Vit D3 31.25µM | | | |
|  | **Set 1** | **Set 2** | **Set 3** | **Set 4** |
| Total Cells | 137 | 227 | 158 | 130 |
| Live Cells | 112 | 178 | 122 | 103 |
| Dead Cells | 25 | 49 | 36 | 27 |
| % Dead Cells | 18.25 | 21.59 | 22.78 | 20.77 |
| **Mean** | 20.85 | | | |
| **SD** | 1.92 | | | |
| **SEM** | 0.96 | | | |

|  | **Vitamin D alone 48 h** | | | |
| --- | --- | --- | --- | --- |
|  | Vit D3 62.5µM | | | |
|  | **Set 1** | **Set 2** | **Set 3** | **Set 4** |
| Total Cells | 240 | 99 | 159 | 116 |
| Live Cells | 126 | 39 | 78 | 60 |
| Dead Cells | 114 | 60 | 81 | 56 |
| % Dead Cells | 47.50 | 60.61 | 50.94 | 48.28 |
| **Mean** | 51.83 | | | |
| **SD** | 6.03 | | | |
| **SEM** | 3.02 | | | |

**Figure 6A:** **The growth of EAC tumors is higher in mice with hyperglycaemia compared to normal mice**

**Tumor Kinetics:**

**Tumor Control (TC)**

| **Days** | **28** | **30** | **32** | **34** | **36** | **38** | **40** | **42** | **44** | **46** |
| --- | --- | --- | --- | --- | --- | --- | --- | --- | --- | --- |
| **Set 1**  **(n=6)** | 93.8 | 177.6 | 226.0 | 245.4 | 349.8 | 445.7 | 559.0 | 716.7 | 919.6 | 1173.5 |
| **Set 2**  **(n=5)** | 118.7 | 577.4 | 857.1 | 852.7 | 936.6 | 965.6 | 1107.5 | 1094.3 |  |  |
| **Set 3**  **(n=5)** | 668.5 | 1011.6 | 1488.6 | 1618.9 | 2309.0 | 2427.0 | 2758.9 | 3005.6 | 3280.0 | 3558.9 |
| **Set 4**  **(n=3)** | 200.7 | 445.9 | 656.4 | 854.6 | 966.1 | 1191.9 | 1225.8 | 1363.0 | 1376.4 | 1449.7 |
| Avg | 270.4 | 553.1 | 807.0 | 892.9 | 1140.4 | 1257.6 | 1412.8 | 1544.9 | 1858.7 | 2060.7 |
| SD | 269.30 | 347.99 | 525.11 | 562.59 | 829.17 | 839.92 | 943.22 | 1009.24 | 1251.90 | 1304.77 |
| SEM | 134.65 | 174.00 | 262.55 | 281.29 | 414.59 | 419.96 | 471.61 | 504.62 | 625.95 | 652.38 |

**STZ+TC**

| **Days** | **28** | **30** | **32** | **34** | **36** | **38** | **40** | **42** | **44** | **46** |
| --- | --- | --- | --- | --- | --- | --- | --- | --- | --- | --- |
| **Set 1**  **(n=4)** | 164.9 | 355.5 | 731.7 | 1259.6 | 1730.7 | 1864.0 | 2210.4 | 2576.6 | 3501.2 | 3640.6 |
| **Set 2**  **(n=3)** | 183.7 | 375.8 | 454.9 | 693.1 | 834.8 | 949.2 | 1227.3 | 1440.4 | 1602.0 | 1620.6 |
| Avg | 174.3 | 365.6 | 593.3 | 976.4 | 1282.7 | 1406.6 | 1718.9 | 2008.5 | 2551.6 | 2630.6 |
| SD | 13.3 | 14.3 | 195.7 | 400.6 | 633.5 | 646.8 | 695.1 | 803.4 | 1342.9 | 1428.3 |
| SEM | 9.4 | 10.2 | 138.8 | 284.1 | 449.3 | 458.7 | 493.0 | 569.8 | 952.4 | 1013.0 |

**Tumor Weights(g):**

|  | **TC** | **STZ+ TC** |
| --- | --- | --- |
|  | 7.1 | 7.4 |
|  | 4.5 | 5.9 |
|  | 7.8 | 9 |
|  | 7.2 | 8.6 |
|  | 4.2 |  |
|  | 6.3 |  |
|  | 1.2 |  |
|  | 4 |  |
|  | 3.4 |  |
|  | 2.6 |  |
|  | 2 |  |
|  | 4.2 |  |
|  | 5.6 |  |
|  | 3.8 |  |
|  | 7.5 |  |
|  | 7.2 |  |
|  | 5.8 |  |
|  | 8.6 |  |
|  | 5.6 |  |
| **Mean (g)** | **5.18** | **7.72** |
| **SD** | **2.09** | **1.39** |
| **SEM** | **0.48** | **0.69** |

**Figure 6B:** **Vitamin D retards the growth of EAC solid tumors in normal and hyperglycaemic mice**

**Tumor Kinetics**

**Tumor Control (TC):**

**STZ+TC**

| **Days** | **28** | **30** | **32** | **34** | **36** | **38** | **40** | **42** | **44** | **46** |
| --- | --- | --- | --- | --- | --- | --- | --- | --- | --- | --- |
| **Set 1**  **(n=6)** | 93.8 | 177.6 | 226.0 | 245.4 | 349.8 | 445.7 | 559.0 | 716.7 | 919.6 | 1173.5 |
| **Set 2**  **(n=5)** | 118.7 | 577.4 | 857.1 | 852.7 | 936.6 | 965.6 | 1107.5 | 1094.3 |  |  |
| **Set 3**  **(n=5)** | 668.5 | 1011.6 | 1488.6 | 1618.9 | 2309.0 | 2427.0 | 2758.9 | 3005.6 | 3280.0 | 3558.9 |
| **Set 4**  **(n=3)** | 200.7 | 445.9 | 656.4 | 854.6 | 966.1 | 1191.9 | 1225.8 | 1363.0 | 1376.4 | 1449.7 |
| Avg | 270.4 | 553.1 | 807.0 | 892.9 | 1140.4 | 1257.6 | 1412.8 | 1544.9 | 1858.7 | 2060.7 |
| SD | 269.30 | 347.99 | 525.11 | 562.59 | 829.17 | 839.92 | 943.22 | 1009.24 | 1251.90 | 1304.77 |
| SEM | 134.65 | 174.00 | 262.55 | 281.29 | 414.59 | 419.96 | 471.61 | 504.62 | 625.95 | 652.38 |

| **Days** | **28** | **30** | **32** | **34** | **36** | **38** | **40** | **42** | **44** | **46** |
| --- | --- | --- | --- | --- | --- | --- | --- | --- | --- | --- |
| **Set 1**  **(n=4)** | 164.9 | 355.5 | 731.7 | 1259.6 | 1730.7 | 1864.0 | 2210.4 | 2576.6 | 3501.2 | 3640.6 |
| **Set 2**  **(n=3)** | 183.7 | 375.8 | 454.9 | 693.1 | 834.8 | 949.2 | 1227.3 | 1440.4 | 1602.0 | 1620.6 |
| Avg | 174.3 | 365.6 | 593.3 | 976.4 | 1282.7 | 1406.6 | 1718.9 | 2008.5 | 2551.6 | 2630.6 |
| SD | 13.3 | 14.3 | 195.7 | 400.6 | 633.5 | 646.8 | 695.1 | 803.4 | 1342.9 | 1428.3 |
| SEM | 9.4 | 10.2 | 138.8 | 284.1 | 449.3 | 458.7 | 493.0 | 569.8 | 952.4 | 1013.0 |

**TC+Vit D3 125µg/Kg**

| **Days** | **28** | **30** | **32** | **34** | **36** | **38** | **40** | **42** | **44** | **46** |
| --- | --- | --- | --- | --- | --- | --- | --- | --- | --- | --- |
| **Set 1**  **(n=5)** | 365.4 | 676.8 | 917.8 | 954.1 | 1094.5 | 966.8 | 940.0 | 760.9 | 622.9 | 526.6 |
| **Set 2**  **(n=5)** | 98.9 | 635.7 | 616.7 | 621.0 | 596.2 | 537.1 | 687.1 | 683.1 |  |  |
| Avg | 232.2 | 656.3 | 767.3 | 787.6 | 845.4 | 752.0 | 813.5 | 722.0 | 622.9 | 526.6 |
| SD | 188.5 | 29.1 | 212.9 | 235.6 | 352.4 | 303.9 | 178.8 | 55.0 | 245.17 | 235.2 |
| SEM | 133.7 | 20.6 | 151.0 | 167.1 | 249.9 | 215.5 | 126.8 | 39.0 | 122.58 | 117.63 |

**STZ+TC+Vit D3 125µg/Kg**

| **Days** | **Tumor Vol**  **(mm^3^)** | **Vol. of no Tumor thigh(mm^3^)** | **Tumor Vol Mean (mm^3^)** | **Vol. of no Tumor thigh(mm^3^)**  **Mean** | **Tumor Vol – Vol of no tumor thigh** | **SD** | **SEM** |
| --- | --- | --- | --- | --- | --- | --- | --- |
| 28 | 1094.9725 | 235.872 | 757.74 | 235.872 | 521.87 | 285.90 | 142.95 |
|  | 580.368 | 235.872 |  |  |  |  |  |
|  | 886.711 | 235.872 |  |  |  |  |  |
|  | 468.93 | 235.872 |  |  |  |  |  |
| 30 | 1188.096 | 235.872 | 1071.66 | 235.872 | 835.79 | 313.31 | 156.65 |
|  | 1498.64 | 235.872 |  |  |  |  |  |
|  | 948.309 | 235.872 |  |  |  |  |  |
|  | 651.63 | 235.872 |  |  |  |  |  |
|  | 991.38 | 235.872 |  |  |  |  |  |
| 32 | 2048 | 235.872 | 1507.819 | 235.872 | 1271.94 | 544.67 | 272.33 |
|  | 1814.121 | 235.872 |  |  |  |  |  |
|  | 1354.88 | 235.872 |  |  |  |  |  |
|  | 814.275 | 235.872 |  |  |  |  |  |
| 34 | 2164.8 | 235.872 | 1930.64 | 235.872 | 1694.77 | 555.58 | 277.79 |
|  | 2103.75 | 235.872 |  |  |  |  |  |
|  | 2220.912 | 235.872 |  |  |  |  |  |
|  | 940.637 | 235.872 |  |  |  |  |  |
|  | 2223.12 | 235.872 |  |  |  |  |  |
| 36 | 1038.96 | 235.872 | 1791.48 | 235.872 | 1555.61 | 773.87 | 386.93 |
|  | 2522.52 | 235.872 |  |  |  |  |  |
|  | 2405.67 | 235.872 |  |  |  |  |  |
|  | 1198.8 | 235.872 |  |  |  |  |  |
|  | 2632.875 | 235.872 |  |  |  |  |  |
| 38 | 898.56 | 235.872 | 1668.55 | 235.872 | 1432.67 | 617.20 | 308.60 |
|  | 2062.26 | 235.872 |  |  |  |  |  |
|  | 2405.67 | 235.872 |  |  |  |  |  |
|  | 1198.8 | 235.872 |  |  |  |  |  |
|  | 1777.4625 | 235.872 |  |  |  |  |  |
| 40 | 1006.5385 | 235.872 | 1241.60 | 235.872 | 1005.73 | 217.69 | 108.84 |
|  | 1335.2175 | 235.872 |  |  |  |  |  |
|  | 1025.959 | 235.872 |  |  |  |  |  |
|  | 1332.738 | 235.872 |  |  |  |  |  |
|  | 1507.572 | 235.872 |  |  |  |  |  |
| 42 | 1160.2085 | 235.872 | 1475.39 | 235.872 | 1239.52 | 392.84 | 196.42 |
|  | 1712.34 | 235.872 |  |  |  |  |  |
|  | 1479.677 | 235.872 |  |  |  |  |  |
|  | 1033.272 | 235.872 |  |  |  |  |  |
|  | 1991.475 | 235.872 |  |  |  |  |  |
| 44 | 737.856 | 235.872 | 1206.203 | 235.872 | 970.331 | 366.12 | 183.06 |
|  | 1712.34 | 235.872 |  |  |  |  |  |
|  | 1169.797 | 235.872 |  |  |  |  |  |
|  | 1033.272 | 235.872 |  |  |  |  |  |
|  | 1377.75 | 235.872 |  |  |  |  |  |
| 46 | 737.856 | 235.872 | 1011.043 | 235.872 | 775.17 | 269.08 | 134.54 |
|  | 1447.335 | 235.872 |  |  |  |  |  |
|  | 1045.845 | 235.872 |  |  |  |  |  |
|  | 868.2355 | 235.872 |  |  |  |  |  |
|  | 955.944 | 235.872 |  |  |  |  |  |

**Tumor Weights(g):**

|  | **TC** | **STZ+ TC** | **TC+Vit D3 125µg/Kg** | **STZ+TC+ Vit D3 125µg/Kg** |
| --- | --- | --- | --- | --- |
|  | 7.1 | 7.4 | 0.6 | 3.8 |
|  | 4.5 | 5.9 | 4.4 | 3.6 |
|  | 7.8 | 9 | 2 | 2.6 |
|  | 7.2 | 8.6 | 2 | 2.2 |
|  | 4.2 |  | 4.2 |  |
|  | 6.3 |  | 3.6 |  |
|  | 1.2 |  | 2.8 |  |
|  | 4 |  | 2 |  |
|  | 3.4 |  | 0.6 |  |
|  | 2.6 |  | 0.6 |  |
|  | 2 |  |  |  |
|  | 4.2 |  |  |  |
|  | 5.6 |  |  |  |
|  | 3.8 |  |  |  |
|  | 7.5 |  |  |  |
|  | 7.2 |  |  |  |
|  | 5.8 |  |  |  |
|  | 8.6 |  |  |  |
|  | 5.6 |  |  |  |
| **Mean (g)** | **5.18** | **7.72** | **2.28** | **3.05** |
| **SD** | **2.09** | **1.39** | **1.44** | **0.77** |
| **SEM** | **0.48** | **0.69** | **0.45** | **0.24** |

**Figure 6C:** **Positive control Cisplatin more effectively retards the growth of EAC soild tumors in hyperglycemic mice**

Values for the groups TC and STZ+TC remain the same as mentioned in 6A and 6B tables.

**TC+PC 2.5mg/Kg**

| **Days** | **28** | **30** | **32** | **34** | **36** | **38** | **40** | **42** | **44** | **46** |
| --- | --- | --- | --- | --- | --- | --- | --- | --- | --- | --- |
| **Set 1**  **(n=6)** | 71.9 | 198.8 | 212.9 | 241.4 | 297.8 | 374.4 | 429.7 | 535.6 | 639.4 | 744.9 |
| **Set 2**  **(n=5)** | 60.9 | 596.7 | 619.0 | 601.2 | 710.8 | 603.4 | 498.5 | 371.2 |  |  |
| **Set 3**  **(n=3)** | 449.5 | 908.3 | 1463.7 | 1540.3 | 1699.2 | 1135.0 | 719.2 | 634.5 | 535.8 | 479.2 |
| Avg | 194.1 | 568.0 | 765.2 | 794.3 | 902.6 | 704.3 | 549.1 | 513.8 | 587.6 | 612.1 |
| SD | 221.2 | 355.6 | 638.1 | 670.6 | 720.1 | 390.2 | 151.2 | 133.0 | 73.3 | 187.8 |
| SEM | 127.9 | 205.6 | 368.9 | 387.7 | 416.2 | 225.6 | 87.4 | 76.9 | 42.4 | 108.6 |

**STZ+TC+PC 2.5mg/Kg**

| **Days** | **Tumor Vol**  **(mm^3^)** | **Vol. of no Tumor thigh(mm^3^)** | **Tumor Vol Mean (mm^3^)** | **Vol. of no Tumor thigh(mm^3^)**  **Mean** | **Tumor Vol – Vol of no tumor thigh** | **SD** | **SEM** |
| --- | --- | --- | --- | --- | --- | --- | --- |
| 28 | 697.779 | 235.872 | 634.58 | 235.872 | 398.71 | 155.471 | 77.73 |
|  | 563.04 | 235.872 |  |  |  |  |  |
|  | 460.768 | 235.872 |  |  |  |  |  |
|  | 816.764 | 235.872 |  |  |  |  |  |
| 30 | 1124.496 | 235.872 | 707.198 | 235.872 | 471.326 | 274.102 | 137.051 |
|  | 377.496 | 235.872 |  |  |  |  |  |
|  | 690.15 | 235.872 |  |  |  |  |  |
|  | 636.65 | 235.872 |  |  |  |  |  |
|  | 831.792 | 235.872 |  |  |  |  |  |
| 32 | 1415.324 | 235.872 | 863.08 | 235.872 | 627.208 | 398.917 | 199.459 |
|  | 495.495 | 235.872 |  |  |  |  |  |
|  | 809.6 | 235.872 |  |  |  |  |  |
|  | 731.901 | 235.872 |  |  |  |  |  |
|  | 1332.63 | 235.872 |  |  |  |  |  |
| 34 | 1896.258 | 235.872 | 1208.279 | 235.872 | 972.406 | 766.5 | 383.25 |
|  | 393.3 | 235.872 |  |  |  |  |  |
|  | 1425.348 | 235.872 |  |  |  |  |  |
|  | 1118.208 | 235.872 |  |  |  |  |  |
|  | 2411.0625 | 235.872 |  |  |  |  |  |
| 36 | 1537.8935 | 235.872 | 1163.477 | 235.872 | 927.60 | 850.67 | 425.33 |
|  | 465.582 | 235.872 |  |  |  |  |  |
|  | 1427.391 | 235.872 |  |  |  |  |  |
|  | 1223.04 | 235.872 |  |  |  |  |  |
|  | 2819.574 | 235.872 |  |  |  |  |  |
| 38 | 1011.197 | 235.872 | 1116.269 | 235.872 | 880.3969 | 500.32 | 250.16 |
|  | 428.922 | 235.872 |  |  |  |  |  |
|  | 1232.28 | 235.872 |  |  |  |  |  |
|  | 1083.264 | 235.872 |  |  |  |  |  |
|  | 1825.6815 | 235.872 |  |  |  |  |  |
| 40 | 966.911 | 235.872 | 1013.832 | 235.872 | 777.96 | 373.88 | 186.94 |
|  | 428.922 | 235.872 |  |  |  |  |  |
|  | 1134 | 235.872 |  |  |  |  |  |
|  | 1083.264 | 235.872 |  |  |  |  |  |
|  | 1456.065 | 235.872 |  |  |  |  |  |
| 42 | 752.136 | 235.872 | 845.1444 | 235.872 | 609.2724 | 235.3428 | 117.6714 |
|  | 494.802 | 235.872 |  |  |  |  |  |
|  | 869.04 | 235.872 |  |  |  |  |  |
|  | 1083.264 | 235.872 |  |  |  |  |  |
|  | 1026.48 | 235.872 |  |  |  |  |  |
| 44 | 450.34 | 235.872 | 751.7466 | 235.872 | 515.87 | 249.09 | 124.54 |
|  | 687.375 | 235.872 |  |  |  |  |  |
|  | 622.16 | 235.872 |  |  |  |  |  |
|  | 916.608 | 235.872 |  |  |  |  |  |
|  | 1082.25 | 235.872 |  |  |  |  |  |
| 46 | 450.34 | 235.872 | 646.1002 | 235.872 | 410.22 | 251.57 | 125.78 |
|  | 555.399 | 235.872 |  |  |  |  |  |
|  | 622.16 | 235.872 |  |  |  |  |  |
|  | 520.352 | 235.872 |  |  |  |  |  |
|  | 1082.25 | 235.872 |  |  |  |  |  |

**Tumor Weights(g):**

|  | **TC** | **STZ TC** | **TC+PC 2.5mg/Kg** | **STZ+TC+ PC 2.5mg/Kg** |
| --- | --- | --- | --- | --- |
|  | 7.1 | 7.4 | 1.8 | 2.8 |
|  | 4.5 | 5.9 | 2 | 0.8 |
|  | 7.8 | 9 | 0.6 | 2.4 |
|  | 7.2 | 8.6 | 1.2 | 2.2 |
|  | 4.2 |  | 1.8 |  |
|  | 6.3 |  | 4.2 |  |
|  | 1.2 |  | 5 |  |
|  | 4 |  | 2.6 |  |
|  | 3.4 |  | 3 |  |
|  | 2.6 |  | 4.8 |  |
|  | 2 |  | 5.2 |  |
|  | 4.2 |  | 2.8 |  |
|  | 5.6 |  | 2.6 |  |
|  | 3.8 |  | 4.4 |  |
|  | 7.5 |  |  |  |
|  | 7.2 |  |  |  |
|  | 5.8 |  |  |  |
|  | 8.6 |  |  |  |
|  | 5.6 |  |  |  |
| **Mean(g)** | **5.18** | **7.72** | **3** | **2.05** |
| **SD** | **2.09** | **1.39** | **1.48** | **0.86** |
| **SEM** | **0.48** | **0.69** | **0.39** | **0.43** |

**Figure 7A: Intraperitoneal and Intratumoral combination administration is more effective compared to just intraperitoneal administration of vitamin D3 in retarding tumor growth**

Values for the TC group remains the same as mentioned in 6A and 6B tables.

**Vit D3 125µg/kg i.p**

| **Days** | **Tumor Vol**  **(mm^3^)** | **Vol. of no Tumor thigh(mm^3^)** | **Tumor Vol Mean (mm^3^)** | **Vol. of no Tumor thigh(mm^3^)**  **Mean** | **Tumor Vol – Vol of no tumor thigh** | | **SD** | **SEM** |
| --- | --- | --- | --- | --- | --- | --- | --- | --- |
| 28 | 89.262 | 17.28 | 116.21 | 17.28 | 98.93 | 39.55 | | 16.21 |
|  | 110.187 | 17.28 |  |  |  |  |  |  |
|  | 94.164 | 17.28 |  |  |  |  |  |  |
|  | 88.6635 | 17.28 |  |  |  |  |  |  |
|  | 122.85 | 17.28 |  |  |  |  |  |  |
|  | 192.192 | 17.28 |  |  |  |  |  |  |
| 30 | 337.311 | 58.656 | 694.34 | 58.656 | 635.68 | 258.49 | | 105.94 |
|  | 813.846 | 58.656 |  |  |  |  |  |  |
|  | 729.6975 | 58.656 |  |  |  |  |  |  |
|  | 584.21 | 58.656 |  |  |  |  |  |  |
|  | 1105.65 | 58.656 |  |  |  |  |  |  |
|  | 595.348 | 58.656 |  |  |  |  |  |  |
| 32 | 339.84 | 58.656 | 675.39 | 58.656 | 616.73 | 256.42 | | 105.093 |
|  | 978.12 | 58.656 |  |  |  |  |  |  |
|  | 677.1 | 58.656 |  |  |  |  |  |  |
|  | 428.022 | 58.656 |  |  |  |  |  |  |
|  | 925.4625 | 58.656 |  |  |  |  |  |  |
|  | 703.8 | 58.656 |  |  |  |  |  |  |
| 34 | 207.138 | 58.656 | 679.65 | 58.656 | 621.00 | 303.52 | | 124.39 |
|  | 781.2 | 58.656 |  |  |  |  |  |  |
|  | 512.7905 | 58.656 |  |  |  |  |  |  |
|  | 1102.08 | 58.656 |  |  |  |  |  |  |
|  | 821.338 | 58.656 |  |  |  |  |  |  |
|  | 653.4 | 58.656 |  |  |  |  |  |  |
| 36 | 171.296 | 58.656 | 654.88 | 58.656 | 596.22 | 328.11 | | 134.47 |
|  | 979.875 | 58.656 |  |  |  |  |  |  |
|  | 556.614 | 58.656 |  |  |  |  |  |  |
|  | 431.244 | 58.656 |  |  |  |  |  |  |
|  | 1004.032 | 58.656 |  |  |  |  |  |  |
|  | 786.24 | 58.656 |  |  |  |  |  |  |
| 38 | 224.037 | 166.532 | 703.644 | 166.532 | 537.11 | 330.92 | | 135.62 |
|  | 1056.384 | 166.532 |  |  |  |  |  |  |
|  | 521.304 | 166.532 |  |  |  |  |  |  |
|  | 516.9825 | 166.532 |  |  |  |  |  |  |
|  | 983.808 | 166.532 |  |  |  |  |  |  |
|  | 919.353 | 166.532 |  |  |  |  |  |  |
| 40 | 198 | 144.4615 | 831.55 | 144.46 | 687.090 | 429.22 | | 175.91 |
|  | 1339.4095 | 144.4615 |  |  |  |  |  |  |
|  | 596.988 | 144.4615 |  |  |  |  |  |  |
|  | 624.078 | 144.4615 |  |  |  |  |  |  |
|  | 1151.621 | 144.4615 |  |  |  |  |  |  |
|  | 1079.2175 | 144.4615 |  |  |  |  |  |  |
| 42 | 226.796 | 144.4615 | 827.570 | 144.46 | 683.10 | 534.14 | | 218.91 |
|  | 556.25 | 144.4615 |  |  |  |  |  |  |
|  | 654.48 | 144.4615 |  |  |  |  |  |  |
|  | 554.7915 | 144.4615 |  |  |  |  |  |  |
|  | 1388.745 | 144.4615 |  |  |  |  |  |  |
|  | 1584.36 | 144.4615 |  |  |  |  |  |  |

**Vit D3 125µg/kg i.p & i.t**

| **Days** | **Tumor Vol**  **(mm^3^)** | **Vol. of no Tumor thigh(mm^3^)** | **Tumor Vol Mean (mm^3^)** | **Vol. of no Tumor thigh(mm^3^)**  **Mean** | **Tumor Vol – Vol of no tumor thigh** | **SD** | **SEM** |
| --- | --- | --- | --- | --- | --- | --- | --- |
| 28 | 565.6 | 235.872 | 601.32 | 235.872 | 365.493 | 260.07 | 106.586 |
|  | 492.7195 | 235.872 |  |  |  |  |  |
|  | 1008.126 | 235.872 |  |  |  |  |  |
|  | 300.468 | 235.872 |  |  |  |  |  |
|  | 639.693 | 235.872 |  |  |  |  |  |
|  |  |  |  |  |  |  |  |
| 30 | 835.78 | 235.872 | 912.7091 | 235.872 | 676.83 | 494.69 | 202.74 |
|  | 785.4165 | 235.872 |  |  |  |  |  |
|  | 291.45 | 235.872 |  |  |  |  |  |
|  | 1664.628 | 235.872 |  |  |  |  |  |
|  | 986.271 | 235.872 |  |  |  |  |  |
|  |  |  |  |  |  |  |  |
| 32 | 834.831 | 235.872 | 1153.6872 | 235.872 | 917.815 | 682.8112 | 279.84 |
|  | 1035.45 | 235.872 |  |  |  |  |  |
|  | 599.664 | 235.872 |  |  |  |  |  |
|  | 2338.875 | 235.872 |  |  |  |  |  |
|  | 959.616 | 235.872 |  |  |  |  |  |
|  |  |  |  |  |  |  |  |
| 34 | 745.143 | 235.872 | 1189.9918 | 235.872 | 954.1198 | 624.2703 | 255.8485 |
|  | 1122.68 | 235.872 |  |  |  |  |  |
|  | 698.775 | 235.872 |  |  |  |  |  |
|  | 2244.743 | 235.872 |  |  |  |  |  |
|  | 1138.619 | 235.872 |  |  |  |  |  |
|  |  |  |  |  |  |  |  |
| 36 | 1013.384 | 235.872 | 1330.41 | 235.872 | 1094.547 | 729.7069 | 299.06 |
|  | 1023.666 | 235.872 |  |  |  |  |  |
|  | 705.672 | 235.872 |  |  |  |  |  |
|  | 2573.375 | 235.872 |  |  |  |  |  |
|  | 1335.996 | 235.872 |  |  |  |  |  |
|  |  |  |  |  |  |  |  |
| 38 | 726.703 | 235.872 | 1202.7098 | 235.872 | 966.83 | 786.73 | 322.43 |
|  | 1023.666 | 235.872 |  |  |  |  |  |
|  | 641.9655 | 235.872 |  |  |  |  |  |
|  | 2573.375 | 235.872 |  |  |  |  |  |
|  | 1047.84 | 235.872 |  |  |  |  |  |
|  |  |  |  |  |  |  |  |
| 40 | 1024.938 | 235.872 | 1175.86 | 235.872 | 939.99 | 507.787 | 208.10 |
|  | 955.189 | 235.872 |  |  |  |  |  |
|  | 528.22 | 235.872 |  |  |  |  |  |
|  | 1582.95 | 235.872 |  |  |  |  |  |
|  | 1788.035 | 235.872 |  |  |  |  |  |
|  |  |  |  |  |  |  |  |
| 42 | 746.172 | 235.872 | 996.7922 | 235.872 | 760.92 | 693.6138 | 284.26 |
|  | 791.9605 | 235.872 |  |  |  |  |  |
|  | 74.844 | 235.872 |  |  |  |  |  |
|  | 1582.95 | 235.872 |  |  |  |  |  |
|  | 1788.035 | 235.872 |  |  |  |  |  |
|  |  |  |  |  |  |  |  |

**7B. Immunohistochemical analysis of the expression of p53**

| Tumor Control | **1** | **2** | **3** | **4** | **Mean** |
| --- | --- | --- | --- | --- | --- |
| Tumor 1 | 73 | 70 | 89 | 94 | 81.5 |
| Tumor 2 | 58 | 72 | 77 | 73 | 70 |
| Tumor 3 | 0 | 0 | 0 | 0 | 0 |
| Tumor 4 | 22 | 24 | 24 | 34 | 26 |
| Tumor 5 | 29 | 32 | 32 | 27 | 30 |
| Tumor 6 | 11 | 12 | 19 | 14 | 14 |
| Tumor 7 | 21 | 24 | 31 | 32 | 27 |
| Tumor 8 | 34 | 35 | 35 | 40 | 36 |
| Tumor 9 | 29 | 39 | 33 | 39 | 35 |
| Tumor 10 | 21 | 19 | 18 | 22 | 20 |
|  | | | | **Mean** | 33.95 |
|  |  |  |  | **SD** | 24.58 |
|  |  |  |  | **SEM** | 7.77 |

| Vehicle  Control | **1** | **2** | **3** | **4** | **Mean** |
| --- | --- | --- | --- | --- | --- |
| Tumor 1 | 30 | 32 | 32 | 29 | 30.75 |
| Tumor 2 | 39 | 35 | 38 | 38 | 37.5 |
| Tumor 3 | 21 | 26 | 29 | 23 | 24.75 |
| Tumor 4 | 21 | 25 | 21 | 19 | 21.5 |
| Tumor 5 | 34 | 30 | 32 | 35 | 32.75 |
| Tumor 6 | 39 | 40 | 41 | 40 | 40 |
| Tumor 7 | 35 | 26 | 27 | 32 | 30 |
| Tumor 8 | 17 | 18 | 22 | 23 | 20 |
| Tumor 9 | 10 | 16 | 15 | 19 | 15 |
| Tumor 10 | 33 | 29 | 35 | 31 | 32 |
|  | | | | **Mean** | 28.425 |
|  |  |  |  | **SD** | 7.94 |
|  |  |  |  | **SEM** | 2.51 |

| Vit D3  i.p & i.t | **1** | **2** | **3** | **4** | **Mean** |
| --- | --- | --- | --- | --- | --- |
| Tumor 1 | 77 | 75 | 74 | 75 | 75.25 |
| Tumor 2 | 59 | 58 | 59 | 63 | 59.75 |
| Tumor 3 | 49 | 45 | 34 | 46 | 43.5 |
| Tumor 4 | 54 | 59 | 55 | 50 | 54.5 |
| Tumor 5 | 55 | 45 | 45 | 43 | 47 |
| Tumor 6 | 43 | 49 | 41 | 42 | 43.75 |
| Tumor 7 | 63 | 63 | 75 | 79 | 70 |
| Tumor 8 | 82 | 75 | 78 | 85 | 80 |
| Tumor 9 | 76 | 77 | 79 | 76 | 77 |
| Tumor 10 | 60 | 54 | 60 | 66 | 60 |
|  | | | | **Mean** | 61.075 |
|  |  |  |  | **SD** | 13.92 |
|  |  |  |  | **SEM** | 4.40 |

| Vit D3 i.p | **1** | **2** | **3** | **4** | **Mean** |
| --- | --- | --- | --- | --- | --- |
| Tumor 1 | 41 | 36 | 42 | 41 | 40 |
| Tumor 2 | 51 | 39 | 35 | 35 | 40 |
| Tumor 3 | 16 | 19 | 20 | 25 | 20 |
| Tumor 4 | 56 | 36 | 62 | 50 | 51 |
| Tumor 5 | 47 | 47 | 39 | 47 | 45 |
| Tumor 6 | 35 | 52 | 51 | 42 | 45 |
| Tumor 7 | 43 | 54 | 41 | 38 | 44 |
|  | | | | **Mean** | 40.71 |
|  |  |  |  | **SD** | 9.86 |
|  |  |  |  | **SEM** | 3.12 |

| Positive  Control | **1** | **2** | **3** | **4** | **Mean** |
| --- | --- | --- | --- | --- | --- |
| Tumor 1 | 32 | 31 | 29 | 36 | 32 |
| Tumor 2 | 32 | 31 | 42 | 47 | 38 |
| Tumor 3 | 41 | 30 | 45 | 44 | 40 |
| Tumor 4 | 46 | 40 | 44 | 46 | 44 |
| Tumor 5 | 36 | 38 | 34 | 32 | 35 |
| Tumor 6 | 83 | 73 | 74 | 78 | 77 |
| Tumor 7 | 65 | 69 | 62 | 68 | 66 |
| Tumor 8 | 66 | 71 | 72 | 71 | 70 |
| Tumor 9 | 87 | 82 | 82 | 89 | 85 |
|  | | | | **Mean** | 54.11 |
|  |  |  |  | **SD** | 20.26 |
|  |  |  |  | **SEM** | 6.41 |

**7C. Immunohistochemical analysis of Ki67 expression**

| Tumor  Control | **1** | **2** | **3** | **4** | **Mean** |
| --- | --- | --- | --- | --- | --- |
| Tumor 1 | 73 | 70 | 68 | 66 | 69.25 |
| Tumor 2 | 69 | 93 | 86 | 78 | 81.5 |
| Tumor 3 | 45 | 43 | 45 | 44 | 44.25 |
| Tumor 4 | 65 | 67 | 68 | 50 | 62.5 |
| Tumor 5 | 81 | 83 | 88 | 90 | 85.5 |
| Tumor 6 | 82 | 75 | 78 | 85 | 80 |
| Tumor 7 | 90 | 90 | 88 | 88 | 89 |
| Tumor 8 | 91 | 89 | 90 | 90 | 90 |
| Tumor 9 | 100 | 100 | 100 | 100 | 100 |
| Tumor 10 | 100 | 100 | 100 | 100 | 100 |
|  | | | | **Mean** | 80.2 |
|  |  |  |  | **SD** | 17.36 |
|  |  |  |  | **SEM** | 5.49 |

| Vehicle Control | **1** | **2** | **3** | **4** | **Mean** |
| --- | --- | --- | --- | --- | --- |
| Tumor 1 | 85 | 86 | 78 | 83 | 83 |
| Tumor 2 | 80 | 72 | 76 | 78 | 76.5 |
| Tumor 3 | 75 | 80 | 76 | 77 | 77 |
| Tumor 4 | 73 | 72 | 72 | 73 | 72.5 |
| Tumor 5 | 70 | 65 | 75 | 70 | 70 |
| Tumor 6 | 65 | 70 | 59 | 58 | 63 |
| Tumor 7 | 69 | 71 | 78 | 70 | 72 |
| Tumor 8 | 92 | 76 | 79 | 85 | 83 |
| Tumor 9 | 83 | 90 | 82 | 85 | 85 |
| Tumor 10 | 68 | 75 | 77 | 88 | 77 |
|  | | | | **Mean** | 75.9 |
|  |  |  |  | **SD** | 6.78 |
|  |  |  |  | **SEM** | 2.14 |

| Vit D3 i.p & i.t | **1** | **2** | **3** | **4** | **Mean** |
| --- | --- | --- | --- | --- | --- |
| Tumor 1 | 54 | 52 | 56 | 58 | 55 |
| Tumor 2 | 59 | 70 | 73 | 62 | 66 |
| Tumor 3 | 38 | 40 | 43 | 33 | 38.5 |
| Tumor 4 | 54 | 60 | 50 | 48 | 53 |
| Tumor 5 | 63 | 59 | 58 | 66 | 61.5 |
| Tumor 6 | 43 | 49 | 52 | 42 | 46.5 |
| Tumor 7 | 52 | 59 | 58 | 51 | 55 |
| Tumor 8 | 27 | 28 | 22 | 23 | 25 |
| Tumor 9 | 52 | 57 | 58 | 49 | 54 |
| Tumor 10 | 29 | 32 | 39 | 28 | 32 |
|  | | | | **Mean** | 48.65 |
|  |  |  |  | **SD** | 13.07 |
|  |  |  |  | **SEM** | 4.137 |

| Vit D3  i.p | **1** | **2** | **3** | **4** | **Mean** |
| --- | --- | --- | --- | --- | --- |
| Tumor 1 | 54 | 60 | 65 | 61 | 60 |
| Tumor 2 | 55 | 59 | 68 | 58 | 60 |
| Tumor 3 | 81 | 85 | 79 | 75 | 80 |
| Tumor 4 | 98 | 91 | 89 | 82 | 90 |
| Tumor 5 | 63 | 66 | 61 | 66 | 64 |
| Tumor 6 | 73 | 70 | 69 | 68 | 70 |
| Tumor 7 | 65 | 69 | 79 | 59 | 68 |
|  | | | | **Mean** | 70.28 |
|  |  |  |  | **SD** | 11.10 |
|  |  |  |  | **SEM** | 3.51 |

| PC | **1** | **2** | **3** | **4** | **Mean** |
| --- | --- | --- | --- | --- | --- |
| Tumor 1 | 32 | 31 | 29 | 36 | 32 |
| Tumor 2 | 31 | 29 | 37 | 47 | 36 |
| Tumor 3 | 25 | 30 | 25 | 28 | 27 |
| Tumor 4 | 29 | 40 | 32 | 35 | 34 |
| Tumor 5 | 28 | 27 | 29 | 32 | 29 |
| Tumor 6 | 32 | 33 | 34 | 33 | 33 |
| Tumor 7 | 12 | 16 | 25 | 27 | 20 |
| Tumor 8 | 49 | 55 | 47 | 49 | 50 |
| Tumor 9 | 25 | 26 | 28 | 25 | 26 |
|  | | | | **Mean** | 31.88 |
|  |  |  |  | **SD** | 8.358 |
|  |  |  |  | **SEM** | 2.64 |

**7D. Immunohistochemical analysis of blood vessel density by CD31 staining**

| Tumor  Control | **1** | **2** | **3** | **4** | **Mean** |
| --- | --- | --- | --- | --- | --- |
| Tumor 1 | 10 | 8 | 8 | 6 | 8 |
| Tumor 2 | 11 | 9 | 6 | 10 | 9 |
| Tumor 3 | 6 | 5 | 7 | 6 | 6 |
| Tumor 4 | 6 | 8 | 11 | 7 | 8 |
| Tumor 5 | 12 | 7 | 8 | 9 | 9 |
| Tumor 6 | 4 | 5 | 7 | 6 | 5.5 |
| Tumor 7 | 12 | 11 | 8 | 5 | 9 |
| Tumor 8 | 9 | 10 | 10 | 11 | 10 |
| Tumor 9 | 7 | 8 | 5 | 8 | 7 |
| Tumor 10 | 8 | 6 | 11 | 7 | 8 |
|  | | | | **Mean** | 7.95 |
|  |  |  |  | **SD** | 1.42 |
|  |  |  |  | **SEM** | 0.45 |

| Vehicle  Control | **1** | **2** | **3** | **4** | **Mean** |
| --- | --- | --- | --- | --- | --- |
| Tumor 1 | 1 | 2 | 3 | 2 | 2 |
| Tumor 2 | 3 | 5 | 6 | 6 | 5 |
| Tumor 3 | 3 | 5 | 6 | 2 | 4 |
| Tumor 4 | 2 | 6 | 5 | 3 | 4 |
| Tumor 5 | 6 | 4 | 6 | 8 | 6 |
| Tumor 6 | 3 | 2 | 5 | 2 | 3 |
| Tumor 7 | 5 | 8 | 6 | 9 | 7 |
| Tumor 8 | 8 | 11 | 11 | 10 | 10 |
| Tumor 9 | 6 | 9 | 9 | 12 | 9 |
| Tumor 10 | 10 | 10 | 5 | 11 | 9 |
|  | | | | **Mean** | 5.9 |
|  |  |  |  | **SD** | 2.76 |
|  |  |  |  | **SEM** | 0.87 |

| Vit D3 i.p & i.t | **1** | **2** | **3** | **4** | **Mean** |
| --- | --- | --- | --- | --- | --- |
| Tumor 1 | 2 | 5 | 6 | 3 | 4 |
| Tumor 2 | 5 | 7 | 8 | 4 | 6 |
| Tumor 3 | 3 | 2 | 3 | 0 | 2 |
| Tumor 4 | 1 | 3 | 5 | 7 | 4 |
| Tumor 5 | 4 | 1 | 2 | 5 | 3 |
| Tumor 6 | 4 | 4 | 3 | 9 | 5 |
| Tumor 7 | 4 | 3 | 2 | 3 | 3 |
| Tumor 8 | 5 | 4 | 1 | 2 | 3 |
| Tumor 9 | 2 | 2 | 1 | 3 | 2 |
| Tumor 10 | 3 | 3 | 0 | 2 | 2 |
|  | | | | **Mean** | 3.4 |
|  |  |  |  | **SD** | 1.34 |
|  |  |  |  | **SEM** | 0.427 |

| Vit D3  i.p | **1** | **2** | **3** | **4** | **Mean** |
| --- | --- | --- | --- | --- | --- |
| Tumor 1 | 1 | 3 | 2 | 2 | 2 |
| Tumor 2 | 2 | 4 | 1 | 1 | 2 |
| Tumor 3 | 3 | 1 | 2 | 3 | 2.25 |
| Tumor 4 | 5 | 4 | 1 | 2 | 3 |
| Tumor 5 | 1 | 3 | 5 | 7 | 4 |
|  | | | | **Mean** | 2.65 |
|  |  |  |  | **SD** | 0.85 |
|  |  |  |  | **SEM** | 0.27 |

| Positive Control | **1** | **2** | **3** | **4** | **Mean** |
| --- | --- | --- | --- | --- | --- |
| Tumor 1 | 1 | 3 | 2 | 2 | 2 |
| Tumor 2 | 1 | 4 | 5 | 2 | 3 |
| Tumor 3 | 2 | 5 | 4 | 1 | 3 |
| Tumor 4 | 2 | 2 | 3 | 1 | 2 |
| Tumor 5 | 3 | 3 | 2 | 0 | 2 |
| Tumor 6 | 1 | 3 | 0 | 0 | 1 |
| Tumor 7 | 1 | 0 | 0 | 0 | 1 |
| Tumor 8 | 2 | 1 | 2 | 3 | 2 |
|  | | | | **Mean** | 2 |
|  |  |  |  | **SD** | 0.75 |
|  |  |  |  | **SEM** | 0.26 |

**Supporting Data: Raw values with Mean, SD and SEM**

**Supporting Data Figure S1: Dose and time dependent decrease in the viability of cancer cells upon treatment with vitamin D3**

1. **Human Hepatocellular Carcinoma-Hep G2**

|  | 24h | | | | | |
| --- | --- | --- | --- | --- | --- | --- |
| **Concentration**  **(µM)** | **1** | **2** | **3** | **Mean** | **SD** | **SEM** |
| **7.813** | 97.47 | 102.89 | 101.49 | 100.62 | 2.81 | 1.62 |
| **15.625** | 123.05 | 100.57 | 98.08 | 107.24 | 13.75 | 7.94 |
| **31.25** | 91.917 | 99.76 | 97.13 | 96.27 | 3.99 | 2.30 |
| **62.5** | 73.09 | 94.82 | 91.56 | 86.49 | 11.71 | 6.77 |
| **125** | 83.28 | 80.06 | 80.06 | 81.13 | 1.85 | 1.07 |
| **250** | 74.63 | 74.29 | 74.29 | 74.41 | 0.19 | 0.11 |
| **500** | 36.77 | 50.42 | 48.05 | 45.08 | 7.29 | 4.21 |

|  | 48h | | | | | |
| --- | --- | --- | --- | --- | --- | --- |
| **Concentration**  **(µM)** | **1** | **2** | **3** | **Mean** | **SD** | **SEM** |
| **7.813** | 108.68 | 89.60 | 97.85 | 98.71 | 9.57 | 5.53 |
| **15.625** | 89.10 | 87.15 | 102.18 | 92.81 | 8.17 | 4.72 |
| **31.25** | 63.02 | 64.84 | 71.76 | 66.54 | 4.61 | 2.66 |
| **62.5** | 31.91 | 18.49 | 27.96 | 26.12 | 6.89 | 3.98 |
| **125** | 30.55 | 21.77 | 21.35 | 24.56 | 5.197 | 3.00 |
| **250** | 25.68 | 21.83 | 21.43 | 22.98 | 2.34 | 1.35 |
| **500** | 27.36 | 22.58 | 21.66 | 23.87 | 3.05 | 1.76 |

|  | 72h | | | | | |
| --- | --- | --- | --- | --- | --- | --- |
| **Concentration**  **(µM)** | **1** | **2** | **3** | **Mean** | **SD** | **SEM** |
| **7.813** | 105.64 | 109.09 | 109 | 107.91 | 1.96 | 1.13 |
| **15.625** | 96.20 | 99.19 | 100 | 98.46 | 1.99 | 1.15 |
| **31.25** | 56.48 | 58.22 | 55 | 56.57 | 1.61 | 0.93 |
| **62.5** | 11.75 | 13.45 | 12 | 12.40 | 0.91 | 0.53 |
| **125** | 16.52 | 16.84 | 15 | 16.12 | 0.98 | 0.57 |
| **250** | 18.04 | 18.46 | 19 | 18.50 | 0.47 | 0.27 |
| **500** | 19.07 | 19.61 | 21 | 19.89 | 0.99 | 0.57 |

1. **Human Colorectal Carcinoma-HT 29**

|  | 24h | | | | | |
| --- | --- | --- | --- | --- | --- | --- |
| **Concentration**  **(µM)** | **1** | **2** | **3** | **Mean** | **SD** | **SEM** |
| **7.813** | 103.77 | 103.17 | 103.2 | 103.38 | 0.33 | 0.19 |
| **15.625** | 104.00 | 103.52 | 103.66 | 103.73 | 0.24 | 0.14 |
| **31.25** | 100.23 | 100.85 | 100.34 | 100.47 | 0.33 | 0.19 |
| **62.5** | 85.54 | 86.34 | 87.34 | 86.40 | 0.90 | 0.52 |
| **125** | 41.96 | 50.04 | 50.2 | 47.40 | 4.71 | 2.72 |
| **250** | 21.45 | 21.12 | 21.2 | 21.26 | 0.17 | 0.09 |
| **500** | 21.28 | 17.08 | 17.02 | 18.46 | 2.44 | 1.41 |

|  | 48h | | | | | |
| --- | --- | --- | --- | --- | --- | --- |
| **Concentration**  **(µM)** | **1** | **2** | **3** | **Mean** | **SD** | **SEM** |
| **7.813** | 98.10 | 113.99 | 101.85 | 104.65 | 8.30 | 4.79 |
| **15.625** | 98.09 | 103.34 | 107.83 | 103.09 | 4.87 | 2.82 |
| **31.25** | 93.97 | 103.72 | 104.32 | 100.67 | 5.80 | 3.35 |
| **62.5** | 87.93 | 100.56 | 95.77 | 94.75 | 6.37 | 3.68 |
| **125** | 77.42 | 76.79 | 71.41 | 75.21 | 3.30 | 1.90 |
| **250** | 34.32 | 36.33 | 47.64 | 39.43 | 7.181 | 4.15 |
| **500** | 45.69 | 39.97 | 46.93 | 44.20 | 3.71 | 2.14 |

|  | 72h | | | | | |
| --- | --- | --- | --- | --- | --- | --- |
| **Concentration**  **(µM)** | **1** | **2** | **3** | **Mean** | **SD** | **SEM** |
| **7.813** | 110.64 | 109.14 | 108.04 | 109.27 | 1.30 | 0.75 |
| **15.625** | 98.71 | 96.27 | 101.96 | 98.98 | 2.85 | 1.65 |
| **31.25** | 99.09 | 98.54 | 101.93 | 99.85 | 1.82 | 1.05 |
| **62.5** | 85.08 | 80.82 | 93.77 | 86.56 | 6.59 | 3.81 |
| **125** | 60.55 | 55.65 | 63.65 | 59.95 | 4.03 | 2.33 |
| **250** | 63.15 | 51.35 | 61.83 | 58.78 | 6.46 | 3.73 |
| **500** | 61.84 | 52.74 | 58.81 | 57.80 | 4.63 | 2.67 |

1. **Human Cervical Carcinoma-SiHa**

|  | 24h | | | | | |
| --- | --- | --- | --- | --- | --- | --- |
| **Concentration**  **(µM)** | **1** | **2** | **3** | **Mean** | **SD** | **SEM** |
| **7.813** | 102.56 | 102.56 | 95.34 | 100.15 | 4.168 | 2.40 |
| **15.625** | 103.07 | 88.26 | 100.55 | 97.30 | 7.92 | 4.58 |
| **31.25** | 103.07 | 86.79 | 94.59 | 94.82 | 8.14 | 4.70 |
| **62.5** | 81.23 | 66.28 | 72.84 | 73.45 | 7.49 | 4.33 |
| **125** | 17.67 | 22.88 | 12.06 | 17.54 | 5.41 | 3.12 |
| **250** | 37.14 | 43.47 | 35.30 | 38.64 | 4.28 | 2.47 |
| **500** | 48.06 | 52.50 | 58.24 | 52.93 | 5.10 | 2.95 |

|  | 48h | | | | | |
| --- | --- | --- | --- | --- | --- | --- |
| **Concentration**  **(µM)** | **1** | **2** | **3** | **Mean** | **SD** | **SEM** |
| **7.813** | 110.07 | 110.07 | 88.31 | 102.82 | 12.56 | 7.26 |
| **15.625** | 114.56 | 110.98 | 88.09 | 104.54 | 14.35 | 8.30 |
| **31.25** | 105.08 | 102.16 | 84.83 | 97.36 | 10.94 | 6.32 |
| **62.5** | 28.29 | 6.07 | 17.57 | 17.31 | 11.11 | 6.42 |
| **125** | 10.71 | 20.32 | 12.47 | 14.50 | 5.11 | 2.95 |
| **250** | 23.90 | 33.87 | 34.60 | 30.79 | 5.98 | 3.45 |
| **500** | 29.39 | 42.99 | 45.56 | 39.31 | 8.68 | 5.02 |

|  | 72h | | | | | |
| --- | --- | --- | --- | --- | --- | --- |
| **Concentration**  **(µM)** | **1** | **2** | **3** | **Mean** | **SD** | **SEM** |
| **7.813** | 118.43 | 90.33 | 90.33 | 99.69 | 16.22 | 9.38 |
| **15.625** | 119.70 | 92.19 | 92.19 | 101.36 | 15.88 | 9.18 |
| **31.25** | 104.35 | 91.17 | 91.17 | 95.57 | 7.60 | 4.39 |
| **62.5** | 3.8147 | 9.24 | 9.24 | 7.43 | 3.13 | 1.81 |
| **125** | 12.71 | 17.30 | 17.30 | 15.77 | 2.64 | 1.53 |
| **250** | 21.16 | 26.54 | 26.54 | 24.75 | 3.10 | 1.79 |
| **500** | 24.34 | 35.11 | 35.11 | 31.52 | 6.21 | 3.59 |

**Fig S3: Treatment of cancer cells with vitamin D3 induced death**

1. **Human Hepatocellular Carcinoma-Hep G2**

**24h**

|  | **Vitamin D alone 24 h** | | | |
| --- | --- | --- | --- | --- |
|  | Control (Untreated) | | | |
|  | **Set 1** | **Set 2** | **Set 3** | **Set 4** |
| Total Cells | 137 | 123 | 201 | 205 |
| Live Cells | 124 | 118 | 185 | 193 |
| Dead Cells | 13 | 5 | 16 | 12 |
| % Dead Cells | 9.49 | 4.07 | 7.96 | 5.85 |
| **Mean** | 6.84 | | | |
| **SD** | 2.38 | | | |
| **SEM** | 1.19 | | | |

|  | **Vitamin D alone 24 h** | | | |
| --- | --- | --- | --- | --- |
|  | Vehicle Control (VC) | | | |
|  | **Set 1** | **Set 2** | **Set 3** | **Set 4** |
| Total Cells | 243 | 271 | 137 | 145 |
| Live Cells | 216 | 236 | 124 | 130 |
| Dead Cells | 27 | 35 | 13 | 15 |
| % Dead Cells | 11.11 | 12.92 | 9.49 | 10.34 |
| **Mean** | 10.97 | | | |
| **SD** | 1.46 | | | |
| **SEM** | 0.73 | | | |

|  | **Vitamin D alone 24 h** | | | |
| --- | --- | --- | --- | --- |
|  | Positive Control (PC) | | | |
|  | **Set 1** | **Set 2** | **Set 3** | **Set 4** |
| Total Cells | 151 | 130 | 165 | 146 |
| Live Cells | 98 | 84 | 106 | 93 |
| Dead Cells | 53 | 46 | 59 | 53 |
| % Dead Cells | 35.09 | 35.38 | 35.75 | 36.30 |
| **Mean** | 35.64 | | | |
| **SD** | 0.52 | | | |
| **SEM** | 0.26 | | | |

|  | **Vitamin D alone 24 h** | | | |
| --- | --- | --- | --- | --- |
|  | Vit D3 62.5µM | | | |
|  | **Set 1** | **Set 2** | **Set 3** | **Set 4** |
| Total Cells | 251 | 222 | 241 | 346 |
| Live Cells | 213 | 193 | 207 | 288 |
| Dead Cells | 38 | 29 | 34 | 58 |
| % Dead Cells | 15.13 | 13.06 | 14.10 | 16.76 |
| **Mean** | 14.77 | | | |
| **SD** | 1.58 | | | |
| **SEM** | 0.79 | | | |

|  | **Vitamin D alone 24 h** | | | |
| --- | --- | --- | --- | --- |
|  | Vit D3 125µM | | | |
|  | **Set 1** | **Set 2** | **Set 3** | **Set 4** |
| Total Cells | 180 | 226 | 196 | 191 |
| Live Cells | 101 | 158 | 133 | 126 |
| Dead Cells | 79 | 68 | 63 | 65 |
| % Dead Cells | 43.88 | 30.08 | 32.14 | 34.03 |
| **Mean** | 35.04 | | | |
| **SD** | 6.12 | | | |
| **SEM** | 3.06 | | | |

|  | **Vitamin D alone 24 h** | | | |
| --- | --- | --- | --- | --- |
|  | Vit D3 250µM | | | |
|  | **Set 1** | **Set 2** | **Set 3** | **Set 4** |
| Total Cells | 140 | 129 | 125 | 121 |
| Live Cells | 11 | 12 | 9 | 12 |
| Dead Cells | 129 | 117 | 116 | 109 |
| % Dead Cells | 92.14 | 90.70 | 92.80 | 90.08 |
| **Mean** | 91.43 | | | |
| **SD** | 1.26 | | | |
| **SEM** | 0.63 | | | |

**48h**

|  | **Vitamin D alone 48 h** | | | |
| --- | --- | --- | --- | --- |
|  | Control(Untreated) | | | |
|  | **Set 1** | **Set 2** | **Set 3** | **Set 4** |
| Total Cells | 138 | 143 | 140 | 121 |
| Live Cells | 124 | 129 | 130 | 116 |
| Dead Cells | 14 | 14 | 10 | 5 |
| % Dead Cells | 10.14 | 9.79 | 7.14 | 4.13 |
| **Mean** | 7.80 | | | |
| **SD** | 2.79 | | | |
| **SEM** | 1.39 | | | |

|  | **Vitamin D alone 48h** | | | |
| --- | --- | --- | --- | --- |
|  | Vehicle Control (VC) | | | |
|  | **Set 1** | **Set 2** | **Set 3** | **Set 4** |
| Total Cells | 243 | 271 | 526 | 537 |
| Live Cells | 216 | 236 | 437 | 445 |
| Dead Cells | 27 | 35 | 89 | 92 |
| % Dead Cells | 11.11 | 12.92 | 16.92 | 17.13 |
| **Mean** | 14.52 | | | |
| **SD** | 2.99 | | | |
| **SEM** | 1.49 | | | |

|  | **Vitamin D alone 48 h** | | | |
| --- | --- | --- | --- | --- |
|  | Positive Control (PC) | | | |
|  | **Set 1** | **Set 2** | **Set 3** | **Set 4** |
| Total Cells | 132 | 117 | 146 | 137 |
| Live Cells | 17 | 9 | 14 | 11 |
| Dead Cells | 115 | 108 | 132 | 126 |
| % Dead Cells | 87.12 | 92.31 | 90.41 | 91.97 |
| **Mean** | 90.45 | | | |
| **SD** | 2.37 | | | |
| **SEM** | 1.18 | | | |

|  | **Vitamin D alone 48 h** | | | |
| --- | --- | --- | --- | --- |
|  | Vit D3 15.625µM | | | |
|  | **Set 1** | **Set 2** | **Set 3** | **Set 4** |
| Total Cells | 240 | 274 | 243 | 250 |
| Live Cells | 214 | 238 | 205 | 210 |
| Dead Cells | 26 | 36 | 38 | 40 |
| % Dead Cells | 10.83 | 13.14 | 15.64 | 16 |
| **Mean** | 13.90 | | | |
| **SD** | 2.41 | | | |
| **SEM** | 1.20 | | | |

|  | **Vitamin D alone 48 h** | | | |
| --- | --- | --- | --- | --- |
|  | Vit D3 31.25µM | | | |
|  | **Set 1** | **Set 2** | **Set 3** | **Set 4** |
| Total Cells | 340 | 278 | 278 | 260 |
| Live Cells | 272 | 203 | 230 | 210 |
| Dead Cells | 68 | 75 | 48 | 50 |
| % Dead Cells | 20 | 26.98 | 17.27 | 19.23 |
| **Mean** | 20.87 | | | |
| **SD** | 4.23 | | | |
| **SEM** | 2.12 | | | |

|  | **Vitamin D alone 48 h** | | | |
| --- | --- | --- | --- | --- |
|  | Vit D3 62.5µM | | | |
|  | **Set 1** | **Set 2** | **Set 3** | **Set 4** |
| Total Cells | 278 | 298 | 312 | 349 |
| Live Cells | 181 | 202 | 194 | 231 |
| Dead Cells | 97 | 96 | 118 | 118 |
| % Dead Cells | 34.89 | 32.21 | 37.82 | 33.81 |
| **Mean** | 34.68 | | | |
| **SD** | 2.36 | | | |
| **SEM** | 1.18 | | | |

1. **Human Colorectal Carcinoma- HT 29**

**24h**

|  | **Vitamin D alone 24 h** | | | |
| --- | --- | --- | --- | --- |
|  | Control (Untreated) | | | |
|  | **Set 1** | **Set 2** | **Set 3** | **Set 4** |
| Total Cells | 123 | 128 | 138 | 144 |
| Live Cells | 107 | 115 | 120 | 125 |
| Dead Cells | 16 | 13 | 18 | 19 |
| % Dead Cells | 13.00 | 10.15 | 13.04 | 13.19 |
| **Mean** | 12.35 | | | |
| **SD** | 1.47 | | | |
| **SEM** | 0.73 | | | |

|  | **Vitamin D alone 24 h** | | | |
| --- | --- | --- | --- | --- |
|  | Vehicle Control (VC) | | | |
|  | **Set 1** | **Set 2** | **Set 3** | **Set 4** |
| Total Cells | 151 | 168 | 168 | 157 |
| Live Cells | 130 | 145 | 150 | 136 |
| Dead Cells | 21 | 23 | 18 | 21 |
| % Dead Cells | 13.90 | 13.69 | 10.71 | 13.38 |
| **Mean** | 12.92 | | | |
| **SD** | 1.49 | | | |
| **SEM** | 0.74 | | | |

|  | **Vitamin D alone 24 h** | | | |
| --- | --- | --- | --- | --- |
|  | Positive Control (PC) | | | |
|  | **Set 1** | **Set 2** | **Set 3** | **Set 4** |
| Total Cells | 183 | 147 | 158 | 167 |
| Live Cells | 143 | 112 | 120 | 130 |
| Dead Cells | 40 | 35 | 38 | 37 |
| % Dead Cells | 21.85 | 23.80 | 24.05 | 22.15 |
| **Mean** | 22.97 | | | |
| **SD** | 1.12 | | | |
| **SEM** | 0.56 | | | |

|  | **Vitamin D alone 24 h** | | | |
| --- | --- | --- | --- | --- |
|  | Vit D3 62.5µM | | | |
|  | **Set 1** | **Set 2** | **Set 3** | **Set 4** |
| Total Cells | 203 | 222 | 233 | 223 |
| Live Cells | 170 | 197 | 201 | 195 |
| Dead Cells | 33 | 25 | 32 | 28 |
| % Dead Cells | 16.25 | 11.26 | 13.73 | 12.55 |
| **Mean** | 13.45 | | | |
| **SD** | 2.12 | | | |
| **SEM** | 1.06 | | | |

|  | **Vitamin D alone 24 h** | | | |
| --- | --- | --- | --- | --- |
|  | Vit D3 125µM | | | |
|  | **Set 1** | **Set 2** | **Set 3** | **Set 4** |
| Total Cells | 268 | 264 | 250 | 267 |
| Live Cells | 205 | 205 | 190 | 200 |
| Dead Cells | 63 | 59 | 60 | 67 |
| % Dead Cells | 23.50 | 22.34 | 24 | 25.09 |
| **Mean** | 23.74 | | | |
| **SD** | 1.14 | | | |
| **SEM** | 0.57 | | | |

|  | **Vitamin D alone 24 h** | | | |
| --- | --- | --- | --- | --- |
|  | Vit D3 250µM | | | |
|  | **Set 1** | **Set 2** | **Set 3** | **Set 4** |
| Total Cells | 170 | 179 | 113 | 161 |
| Live Cells | 39 | 36 | 34 | 51 |
| Dead Cells | 131 | 143 | 79 | 110 |
| % Dead Cells | 77.05 | 79.88 | 69.91 | 68.32 |
| **Mean** | 73.80 | | | |
| **SD** | 5.56 | | | |
| **SEM** | 2.78 | | | |

**48h**

|  | **Vitamin D alone 48 h** | | | |
| --- | --- | --- | --- | --- |
|  | Control (Untreated) | | | |
|  | **Set 1** | **Set 2** | **Set 3** | **Set 4** |
| Total Cells | 157 | 228 | 242 | 250 |
| Live Cells | 134 | 202 | 210 | 220 |
| Dead Cells | 23 | 26 | 32 | 30 |
| % Dead Cells | 14.64 | 11.40 | 13.22 | 12 |
| **Mean** | 12.82 | | | |
| **SD** | 1.44 | | | |
| **SEM** | 0.72 | | | |

|  | **Vitamin D alone 48h** | | | |
| --- | --- | --- | --- | --- |
|  | Vehicle Control (VC) | | | |
|  | **Set 1** | **Set 2** | **Set 3** | **Set 4** |
| Total Cells | 172 | 205 | 242 | 235 |
| Live Cells | 150 | 180 | 210 | 210 |
| Dead Cells | 22 | 25 | 32 | 25 |
| % Dead Cells | 12.79 | 12.19 | 13.2 | 10.64 |
| **Mean** | 12.21 | | | |
| **SD** | 1.13 | | | |
| **SEM** | 0.57 | | | |

|  | **Vitamin D alone 48 h** | | | |
| --- | --- | --- | --- | --- |
|  | Positive Control (PC) | | | |
|  | **Set 1** | **Set 2** | **Set 3** | **Set 4** |
| Total Cells | 116 | 142 | 145 | 137 |
| Live Cells | 73 | 92 | 86 | 82 |
| Dead Cells | 43 | 50 | 59 | 55 |
| % Dead Cells | 37.06 | 35.21 | 40.68 | 40.14 |
| **Mean** | 38.28 | | | |
| **SD** | 2.59 | | | |
| **SEM** | 1.30 | | | |

|  | **Vitamin D alone 48 h** | | | |
| --- | --- | --- | --- | --- |
|  | Vit D3 31.25µM | | | |
|  | **Set 1** | **Set 2** | **Set 3** | **Set 4** |
| Total Cells | 214 | 209 | 265 | 282 |
| Live Cells | 183 | 190 | 220 | 230 |
| Dead Cells | 31 | 19 | 45 | 52 |
| % Dead Cells | 14.48 | 9.09 | 16.98 | 18.43 |
| **Mean** | 14.75 | | | |
| **SD** | 4.11 | | | |
| **SEM** | 2.06 | | | |

|  | **Vitamin D alone 48 h** | | | |
| --- | --- | --- | --- | --- |
|  | Vit D3 62.5µM | | | |
|  | **Set 1** | **Set 2** | **Set 3** | **Set 4** |
| Total Cells | 210 | 248 | 268 | 279 |
| Live Cells | 161 | 166 | 180 | 189 |
| Dead Cells | 49 | 82 | 88 | 90 |
| % Dead Cells | 23.33 | 33.06 | 32.83 | 32.25 |
| **Mean** | 30.37 | | | |
| **SD** | 4.71 | | | |
| **SEM** | 2.35 | | | |

|  | **Vitamin D alone 48 h** | | | |
| --- | --- | --- | --- | --- |
|  | Vit D3 125µM | | | |
|  | **Set 1** | **Set 2** | **Set 3** | **Set 4** |
| Total Cells | 92 | 75 | 82 | 79 |
| Live Cells | 32 | 35 | 32 | 37 |
| Dead Cells | 60 | 40 | 50 | 42 |
| % Dead Cells | 65.22 | 53.33 | 60.98 | 53.16 |
| **Mean** | 58.17 | | | |
| **SD** | 5.94 | | | |
| **SEM** | 2.97 | | | |

1. **Human Cervical Carcinoma-SiHa**

**24h**

|  | **Vitamin D alone 24 h** | | | |
| --- | --- | --- | --- | --- |
|  | Control (Untreated) | | | |
|  | **Set 1** | **Set 2** | **Set 3** | **Set 4** |
| Total Cells | 160 | 131 | 175 | 184 |
| Live Cells | 153 | 121 | 164 | 170 |
| Dead Cells | 7 | 10 | 11 | 14 |
| % Dead Cells | 4.375 | 7.63 | 6.28 | 7.61 |
| **Mean** | 6.48 | | | |
| **SD** | 1.54 | | | |
| **SEM** | 0.77 | | | |

|  | **Vitamin D alone 24 h** | | | |
| --- | --- | --- | --- | --- |
|  | Vehicle Control (VC) | | | |
|  | **Set 1** | **Set 2** | **Set 3** | **Set 4** |
| Total Cells | 229 | 168 | 215 | 225 |
| Live Cells | 210 | 142 | 203 | 210 |
| Dead Cells | 19 | 26 | 12 | 15 |
| % Dead Cells | 8.29 | 15.47 | 5.58 | 6.67 |
| **Mean** | 9.01 | | | |
| **SD** | 4.46 | | | |
| **SEM** | 2.23 | | | |

|  | **Vitamin D alone 24 h** | | | |
| --- | --- | --- | --- | --- |
|  | Positive Control (PC) | | | |
|  | **Set 1** | **Set 2** | **Set 3** | **Set 4** |
| Total Cells | 110 | 89 | 81 | 90 |
| Live Cells | 25 | 11 | 8 | 10 |
| Dead Cells | 85 | 78 | 73 | 80 |
| % Dead Cells | 77.27 | 87.64 | 90.12 | 88.88 |
| **Mean** | 85.98 | | | |
| **SD** | 5.89 | | | |
| **SEM** | 2.95 | | | |

|  | **Vitamin D alone 24 h** | | | |
| --- | --- | --- | --- | --- |
|  | Vit D3 62.5µM | | | |
|  | **Set 1** | **Set 2** | **Set 3** | **Set 4** |
| Total Cells | 273 | 287 | 254 | 272 |
| Live Cells | 238 | 250 | 220 | 230 |
| Dead Cells | 35 | 37 | 34 | 42 |
| % Dead Cells | 12.82 | 12.89 | 13.38 | 15.44 |
| **Mean** | 13.63 | | | |
| **SD** | 1.23 | | | |
| **SEM** | 0.62 | | | |

|  | **Vitamin D alone 24 h** | | | |
| --- | --- | --- | --- | --- |
|  | Vit D3 125µM | | | |
|  | **Set 1** | **Set 2** | **Set 3** | **Set 4** |
| Total Cells | 235 | 134 | 151 | 152 |
| Live Cells | 185 | 97 | 106 | 110 |
| Dead Cells | 50 | 37 | 45 | 42 |
| % Dead Cells | 21.27 | 27.61 | 29.80 | 27.63 |
| **Mean** | 26.58 | | | |
| **SD** | 3.68 | | | |
| **SEM** | 1.84 | | | |

|  | **Vitamin D alone 24 h** | | | |
| --- | --- | --- | --- | --- |
|  | Vit D3 250µM | | | |
|  | **Set 1** | **Set 2** | **Set 3** | **Set 4** |
| Total Cells | 80 | 101 | 96 | 99 |
| Live Cells | 12 | 12 | 8 | 9 |
| Dead Cells | 68 | 89 | 88 | 90 |
| % Dead Cells | 85 | 88.12 | 91.67 | 90.91 |
| **Mean** | 88.92 | | | |
| **SD** | 3.03 | | | |
| **SEM** | 1.51 | | | |

**48h**

|  | **Vitamin D alone 48 h** | | | |
| --- | --- | --- | --- | --- |
|  | Control (Untreated) | | | |
|  | **Set 1** | **Set 2** | **Set 3** | **Set 4** |
| Total Cells | 167 | 137 | 166 | 172 |
| Live Cells | 159 | 128 | 156 | 160 |
| Dead Cells | 8 | 9 | 10 | 12 |
| % Dead Cells | 4.79 | 6.57 | 6.02 | 6.98 |
| **Mean** | 6.09 | | | |
| **SD** | 0.95 | | | |
| **SEM** | 0.48 | | | |

|  | **Vitamin D alone 48h** | | | |
| --- | --- | --- | --- | --- |
|  | Vehicle Control (VC) | | | |
|  | **Set 1** | **Set 2** | **Set 3** | **Set 4** |
| Total Cells | 186 | 208 | 164 | 198 |
| Live Cells | 163 | 191 | 157 | 189 |
| Dead Cells | 23 | 17 | 7 | 9 |
| % Dead Cells | 12.36 | 8.17 | 4.26 | 4.55 |
| **Mean** | 7.34 | | | |
| **SD** | 3.79 | | | |
| **SEM** | 1.90 | | | |

|  | **Vitamin D alone 48 h** | | | |
| --- | --- | --- | --- | --- |
|  | Positive Control (PC) | | | |
|  | **Set 1** | **Set 2** | **Set 3** | **Set 4** |
| Total Cells | 57 | 57 | 61 | 65 |
| Live Cells | 3 | 1 | 2 | 5 |
| Dead Cells | 54 | 56 | 59 | 60 |
| % Dead Cells | 94.73 | 98.24 | 96.72 | 92.30 |
| **Mean** | 95.50 | | | |
| **SD** | 2.57 | | | |
| **SEM** | 1.28 | | | |

|  | **Vitamin D alone 48 h** | | | |
| --- | --- | --- | --- | --- |
|  | Vit D3 62.5µM | | | |
|  | **Set 1** | **Set 2** | **Set 3** | **Set 4** |
| Total Cells | 98 | 110 | 95 | 118 |
| Live Cells | 15 | 17 | 15 | 20 |
| Dead Cells | 83 | 93 | 80 | 98 |
| % Dead Cells | 84.69 | 84.54 | 84.21 | 83.05 |
| **Mean** | 84.13 | | | |
| **SD** | 0.74 | | | |
| **SEM** | 0.37 | | | |

|  | **Vitamin D alone 48 h** | | | |
| --- | --- | --- | --- | --- |
|  | Vit D3 125µM | | | |
|  | **Set 1** | **Set 2** | **Set 3** | **Set 4** |
| Total Cells | 69 | 82 | 73 | 72 |
| Live Cells | 13 | 14 | 15 | 12 |
| Dead Cells | 56 | 68 | 58 | 60 |
| % Dead Cells | 81.15 | 82.92 | 79.45 | 83.33 |
| **Mean** | 81.72 | | | |
| **SD** | 1.78 | | | |
| **SEM** | 0.89 | | | |

|  | **Vitamin D alone 48 h** | | | |
| --- | --- | --- | --- | --- |
|  | Vit D3 250µM | | | |
|  | **Set 1** | **Set 2** | **Set 3** | **Set 4** |
| Total Cells | 72 | 57 | 89 | 110 |
| Live Cells | 7 | 2 | 9 | 12 |
| Dead Cells | 65 | 55 | 80 | 98 |
| % Dead Cells | 90.28 | 96.49 | 89.89 | 89.09 |
| **Mean** | 91.44 | | | |
| **SD** | 3.41 | | | |
| **SEM** | 1.70 | | | |

**Supporting Data Figure S5: Vitamin D3, but not the Cisplatin, could moderately reduce STZ-induced hyperglycaemia in mice**

**b)**

**Normal Control**

| **Days** | FBG(mg/dL) | Mean | SD | SEM |
| --- | --- | --- | --- | --- |
| 6 | 121 | 102.4 | 12.64 | 5.67 |
|  | 109 |  |  |  |
|  | 97 |  |  |  |
|  | 89 |  |  |  |
|  | 96 |  |  |  |
| 8 | 100 | 107.8 | 12.91 | 5.79 |
|  | 121 |  |  |  |
|  | 92 |  |  |  |
|  | 105 |  |  |  |
|  | 121 |  |  |  |
| 10 | 98 | 96 | 19.33 | 8.67 |
|  | 68 |  |  |  |
|  | 120 |  |  |  |
|  | 105 |  |  |  |
|  | 89 |  |  |  |
| 12 | 98 | 102.6 | 12.03 | 5.40 |
|  | 108 |  |  |  |
|  | 88 |  |  |  |
|  | 120 |  |  |  |
|  | 99 |  |  |  |
| 14 | 98 | 102.6 | 12.03 | 5.40 |
|  | 108 |  |  |  |
|  | 88 |  |  |  |
|  | 120 |  |  |  |
|  | 99 |  |  |  |
| 16 | 98 | 102.8 | 11.95 | 5.36 |
|  | 110 |  |  |  |
|  | 88 |  |  |  |
|  | 119 |  |  |  |
|  | 99 |  |  |  |
| 18 | 101 | 107 | 11.14 | 4.99 |
|  | 121 |  |  |  |
|  | 93 |  |  |  |
|  | 105 |  |  |  |
|  | 115 |  |  |  |
| 20 | 100 | 96 | 8.34 | 3.74 |
|  | 93 |  |  |  |
|  | 86 |  |  |  |
|  | 108 |  |  |  |
|  | 93 |  |  |  |
| 22 | 115 | 102.8 | 8.35 | 3.74 |
|  | 102 |  |  |  |
|  | 92 |  |  |  |
|  | 105 |  |  |  |
|  | 100 |  |  |  |
| 24 | 115 | 102.2 | 11.14 | 4.99 |
|  | 102 |  |  |  |
|  | 91 |  |  |  |
|  | 103 |  |  |  |
|  | 100 |  |  |  |
| 26 | 130 | 116.2 | 18.36 | 8.23 |
|  | 108 |  |  |  |
|  | 88 |  |  |  |
|  | 132 |  |  |  |
|  | 123 |  |  |  |
| 28 | 120 | 115.4 | 10.01 | 4.49 |
|  | 125 |  |  |  |
|  | 111 |  |  |  |
|  | 100 |  |  |  |
|  | 121 |  |  |  |

**STZ+TC**

| **Days** | FBG | Mean | SD | SEM |
| --- | --- | --- | --- | --- |
| 6 | 128 | 132.2 | 13.56466 | 6.08 |
|  | 154 |  |  |  |
|  | 124 |  |  |  |
|  | 130 |  |  |  |
|  | 125 |  |  |  |
| 8 | 118 | 126.8 | 16.96172 | 7.61 |
|  | 113 |  |  |  |
|  | 155 |  |  |  |
|  | 118 |  |  |  |
|  | 130 |  |  |  |
| 10 | 162 | 161.8 | 4.969909 | 2.23 |
|  | 170 |  |  |  |
|  | 161 |  |  |  |
|  | 157 |  |  |  |
|  | 159 |  |  |  |
| 12 | 160 | 148.6 | 30.87556 | 13.85 |
|  | 103 |  |  |  |
|  | 163 |  |  |  |
|  | 134 |  |  |  |
|  | 183 |  |  |  |
| 14 | 162 | 175.8 | 14.49828 | 6.50 |
|  | 180 |  |  |  |
|  | 159 |  |  |  |
|  | 188 |  |  |  |
|  | 190 |  |  |  |
| 16 | 196 | 182.6 | 13.40895 | 6.01 |
|  | 190 |  |  |  |
|  | 177 |  |  |  |
|  | 188 |  |  |  |
|  | 162 |  |  |  |
| 18 | 176 | 186.6 | 10.43072 | 4.68 |
|  | 184 |  |  |  |
|  | 199 |  |  |  |
|  | 178 |  |  |  |
|  | 196 |  |  |  |
| 20 | 188 | 196.6 | 8.905055 | 3.99 |
|  | 197 |  |  |  |
|  | 189 |  |  |  |
|  | 210 |  |  |  |
|  | 199 |  |  |  |
| 22 | 191 | 195.6 | 10.94532 | 4.91 |
|  | 197 |  |  |  |
|  | 181 |  |  |  |
|  | 211 |  |  |  |
|  | 198 |  |  |  |
| 24 | 188 | 196.6 | 8.905055 | 3.99 |
|  | 197 |  |  |  |
|  | 189 |  |  |  |
|  | 210 |  |  |  |
|  | 199 |  |  |  |
| 26 | 211 | 207.4 | 26.08256 | 11.70 |
|  | 182 |  |  |  |
|  | 193 |  |  |  |
|  | 250 |  |  |  |
|  | 201 |  |  |  |
| 28 | 204 | 198.8 | 20.84946 | 9.35 |
|  | 179 |  |  |  |
|  | 182 |  |  |  |
|  | 231 |  |  |  |
|  | 198 |  |  |  |

**C)**

**Fasting blood glucose**

**Normal Control**

| **Days** | FBG (mg/dL) | Mean | SD | SEM |
| --- | --- | --- | --- | --- |
| 28 | 100 | 96 | 8.337 | 3.738 |
|  | 93 |  |  |  |
|  | 86 |  |  |  |
|  | 108 |  |  |  |
|  | 93 |  |  |  |
| 34 | 98 | 102.6 | 12.033 | 5.396 |
|  | 108 |  |  |  |
|  | 88 |  |  |  |
|  | 120 |  |  |  |
|  | 99 |  |  |  |
| 40 | 130 | 116.2 | 18.363 | 8.235 |
|  | 108 |  |  |  |
|  | 88 |  |  |  |
|  | 132 |  |  |  |
|  | 123 |  |  |  |
| 46 | 120 | 115.4 | 10.015 | 4.491 |
|  | 125 |  |  |  |
|  | 111 |  |  |  |
|  | 100 |  |  |  |
|  | 121 |  |  |  |

**STZ+TC**

| **Days** | FBG(mg/dL) | Mean | SD | SEM |
| --- | --- | --- | --- | --- |
| 28 | 188 | 196.6 | 8.91 | 3.993 |
|  | 197 |  |  |  |
|  | 189 |  |  |  |
|  | 210 |  |  |  |
|  | 199 |  |  |  |
| 34 | 191 | 195.6 | 10.95 | 4.908 |
|  | 197 |  |  |  |
|  | 181 |  |  |  |
|  | 211 |  |  |  |
|  | 198 |  |  |  |
| 40 | 188 | 196.6 | 8.91 | 3.993 |
|  | 197 |  |  |  |
|  | 189 |  |  |  |
|  | 210 |  |  |  |
|  | 199 |  |  |  |
| 46 | 211 | 207.4 | 26.08 | 11.696 |
|  | 182 |  |  |  |
|  | 193 |  |  |  |
|  | 250 |  |  |  |
|  | 201 |  |  |  |

**STZ+VC**

| **Days** | FBG(mg/dL) | Mean | SD | SEM |
| --- | --- | --- | --- | --- |
| 28 | 200 | 198.2 | 6.099 | 2.735 |
|  | 198 |  |  |  |
|  | 204 |  |  |  |
|  | 201 |  |  |  |
|  | 188 |  |  |  |
| 34 | 201 | 203 | 5.148 | 2.308 |
|  | 199 |  |  |  |
|  | 209 |  |  |  |
|  | 208 |  |  |  |
|  | 198 |  |  |  |
| 40 | 201 | 195 | 16.416 | 7.362 |
|  | 179 |  |  |  |
|  | 211 |  |  |  |
|  | 208 |  |  |  |
|  | 176 |  |  |  |
| 46 | 250 | 216.6 | 27.024 | 12.118 |
|  | 185 |  |  |  |
|  | 225 |  |  |  |
|  | 193 |  |  |  |
|  | 230 |  |  |  |

**STZ+PC**

| **Days** | FBG(mg/dL) | Mean | SD | SEM |
| --- | --- | --- | --- | --- |
| 28 | 192 | 198.6 | 14.98 | 6.72 |
|  | 180 |  |  |  |
|  | 199 |  |  |  |
|  | 201 |  |  |  |
|  | 221 |  |  |  |
| 34 | 193 | 200 | 16.12 | 7.23 |
|  | 181 |  |  |  |
|  | 199 |  |  |  |
|  | 202 |  |  |  |
|  | 225 |  |  |  |
| 40 | 292 | 221.4 | 43.05 | 19.30 |
|  | 179 |  |  |  |
|  | 198 |  |  |  |
|  | 213 |  |  |  |
|  | 225 |  |  |  |
| 46 | 323 | 239.6 | 50.45 | 22.62 |
|  | 191 |  |  |  |
|  | 211 |  |  |  |
|  | 240 |  |  |  |
|  | 233 |  |  |  |

**STZ+Vit D 125µg/Kg**

| **Days** | FBG(mg/dL) | Mean | SD | SEM |
| --- | --- | --- | --- | --- |
| 28 | 189 | 210.8 | 25.3 | 11.3 |
|  | 189 |  |  |  |
|  | 244 |  |  |  |
|  | 201 |  |  |  |
|  | 231 |  |  |  |
| 34 | 188 | 199.4 | 10.1 | 4.5 |
|  | 206 |  |  |  |
|  | 209 |  |  |  |
|  | 189 |  |  |  |
|  | 205 |  |  |  |
| 40 | 180 | 193 | 10.0 | 4.5 |
|  | 201 |  |  |  |
|  | 189 |  |  |  |
|  | 190 |  |  |  |
|  | 205 |  |  |  |
| 46 | 180 | 192.8 | 10.1 | 4.5 |
|  | 201 |  |  |  |
|  | 189 |  |  |  |
|  | 189 |  |  |  |
|  | 205 |  |  |  |
