# Supplementary material for: Vitamin D3 reduces the viability of cancer cells in vitro and retard the EAC tumors growth in mice
Source: PLoS One. 2025 Sep 8;20(9):e0331306. doi: 10.1371/journal.pone.0331306 (PMC12416751; doi:10.1371/journal.pone.0331306)
Supplement: S1 Image — (PDF) [file pone.0331306.s013.pdf]

Hep 3B-Original blots

Vitamin D receptor (VDR)

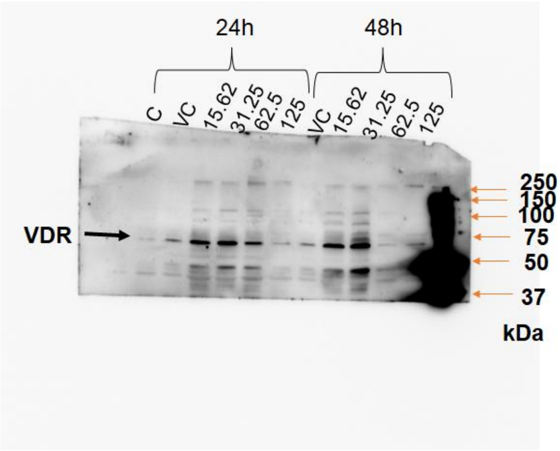

Cut blot depicting VDR band

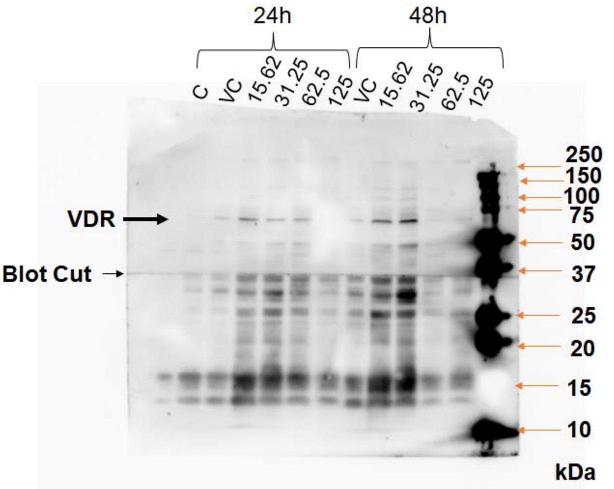

Complete blot depicting VDR band

Catalog Number : sc-13133

The protein bands were visualized using ECL (Clarity Western ECL substrate, Biorad 170-5060) followed by capturing the image in a Chemidoc system (Alliance Q9 UVITec Cambridge UK).

Hep 3B -p53

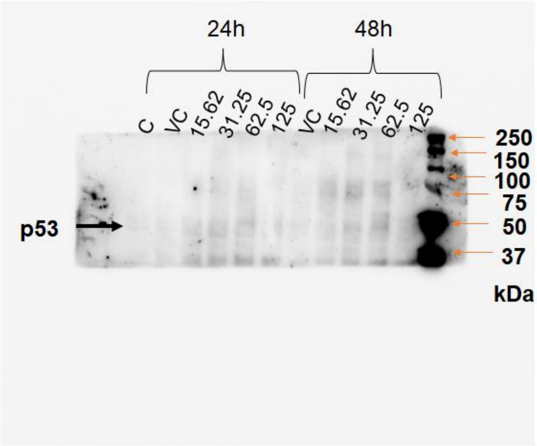

Cut blot depicting p53 band

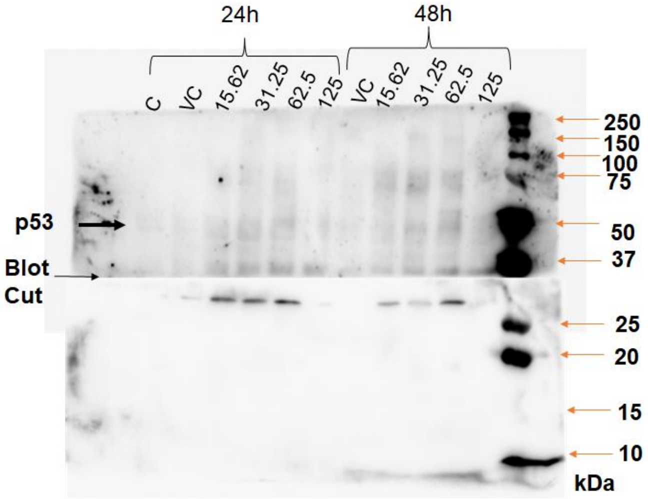

Complete blot depicting p53 band

Catalog Number : 9282

The protein bands were visualized using ECL (Clarity Western ECL substrate, Biorad 170-5060) followed by capturing the image in a Chemidoc system (Alliance Q9 UVITec Cambridge UK).

Hep 3B-Bax

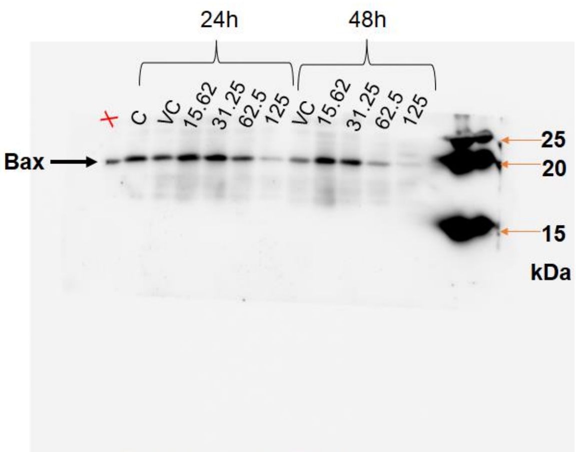

Cut blot depicting Bax band

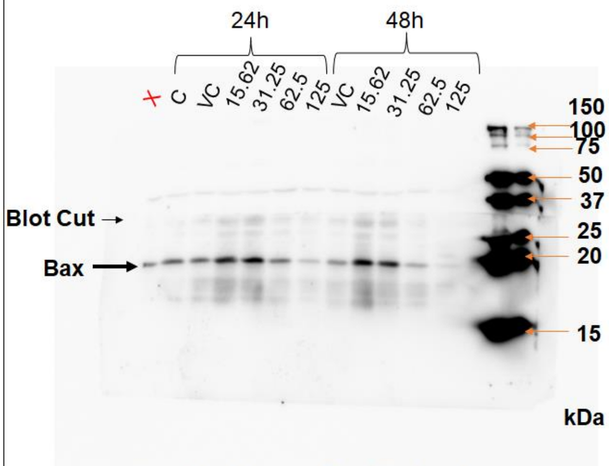

Complete blot depicting Bax band

Catalog Number : 5023s

The protein bands were visualized using ECL (Clarity Western ECL substrate, Biorad 170-5060) followed by capturing the image in a Chemidoc system (Alliance Q9 UVITec Cambridge UK).

Hep 3B-Bcl2

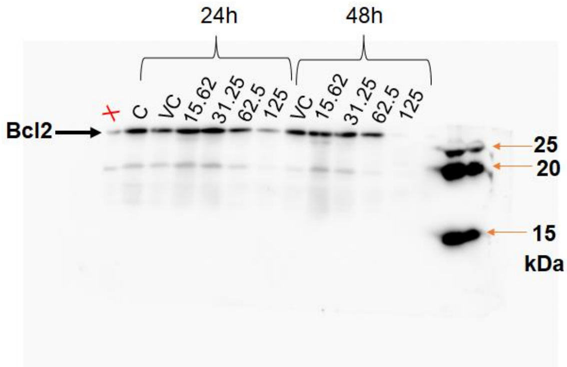

Cut blot depicting Bcl2 band

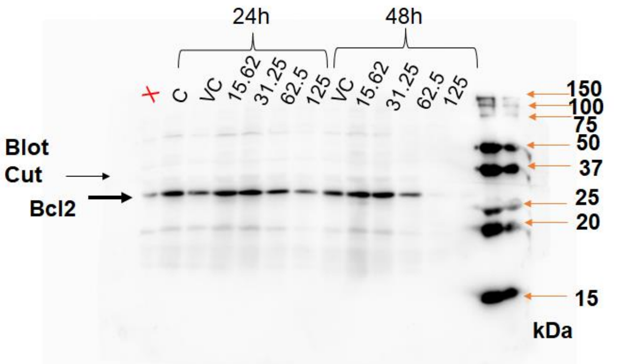

Complete blot depicting Bcl2 band

Catalog Number : PAA778hu01

The protein bands were visualized using ECL (Clarity Western ECL substrate, Biorad 170-5060) followed by capturing the image in a Chemidoc system (Alliance Q9 UVITec Cambridge UK).

Hep 3B-Cyclin D1

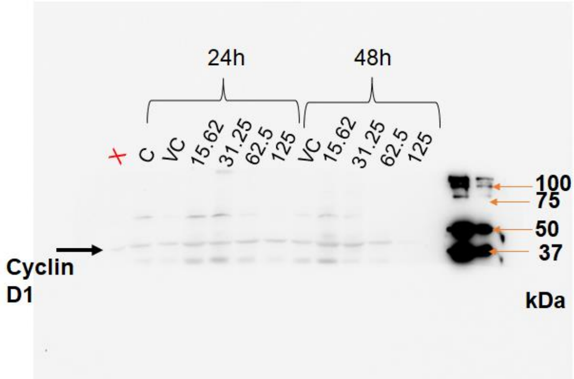

Cut blot depicting Cyclin D1 band

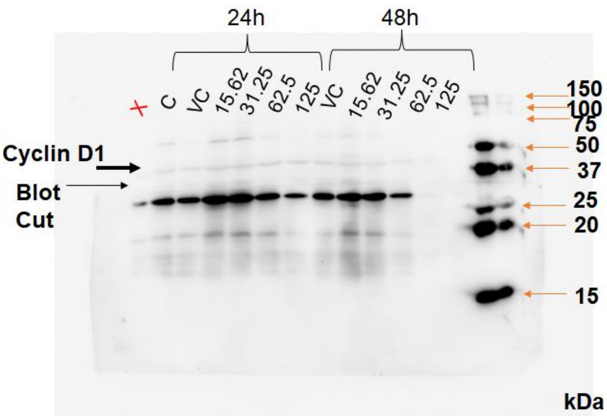

Complete blot depicting Cyclin D1 band

Catalog Number : sc-8396

The protein bands were visualized using ECL (Clarity Western ECL substrate, Biorad 170-5060) followed by capturing the image in a Chemidoc system (Alliance Q9 UVITec Cambridge UK).

Hep 3B-p21

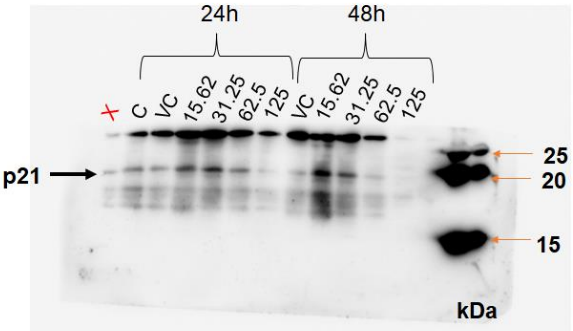

Cut blot depicting p21 band

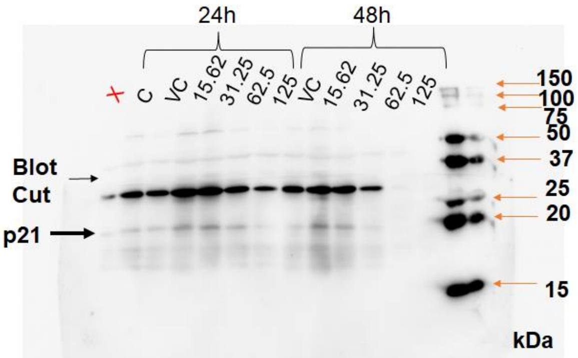

Complete blot depicting p21 band

Catalog Number : sc-6246

The protein bands were visualized using ECL (Clarity Western ECL substrate, Biorad 170-5060) followed by capturing the image in a Chemidoc system (Alliance Q9 UVITec Cambridge UK).

Hep 3B- Survivin

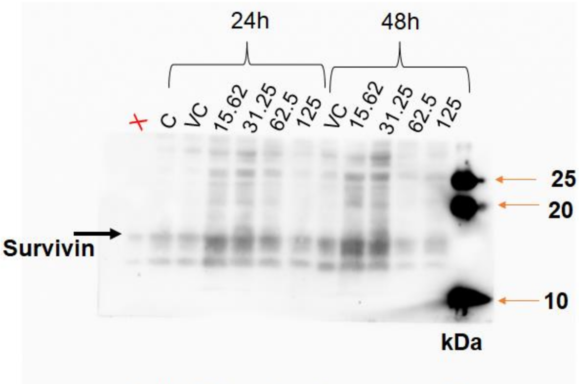

Cut blot depicting Survivin band

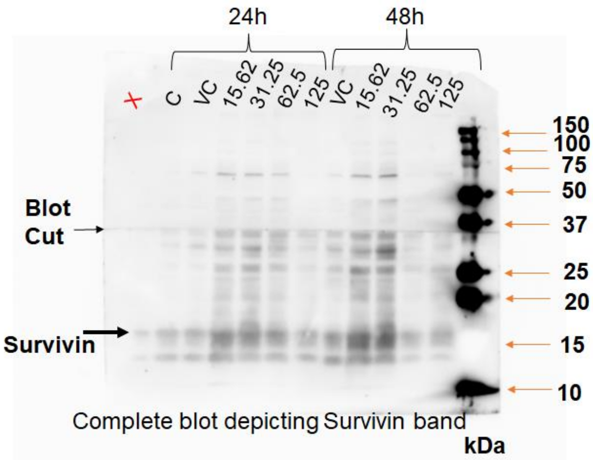

Complete blot depicting Survivin band

Catalog Number : 2803s

The protein bands were visualized using ECL (Clarity Western ECL substrate, Biorad 170-5060) followed by capturing the image in a Chemidoc system (Alliance Q9 UVITec Cambridge UK).

Hep 3B-Beta Actin

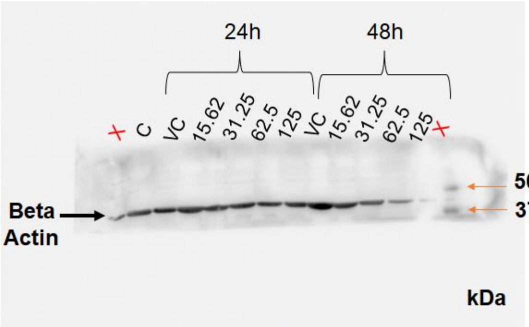

Cut blot depicting Beta Actin band

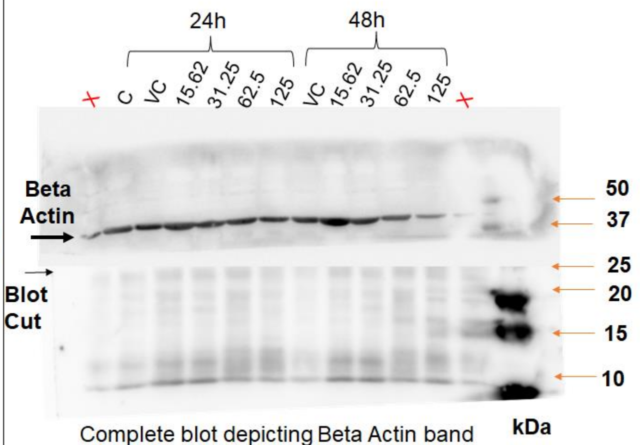

Complete blot depicting Beta Actin band

Catalog Number : CAB340Mi22

The protein bands were visualized using ECL (Clarity Western ECL substrate, Biorad 170-5060) followed by capturing the image in a Chemidoc system (Alliance Q9 UVITec Cambridge UK).

HCT 116-Original blots

Vitamin D receptor (VDR)

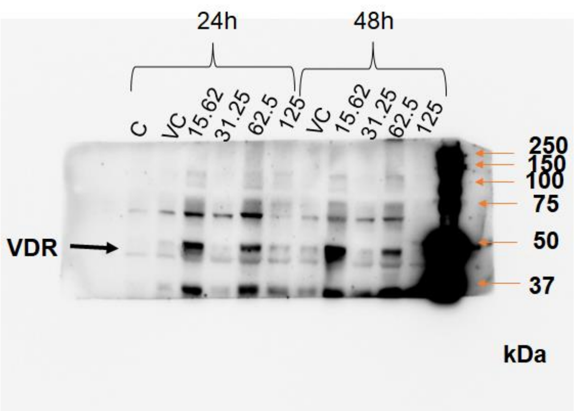

Cut blot depicting VDR band

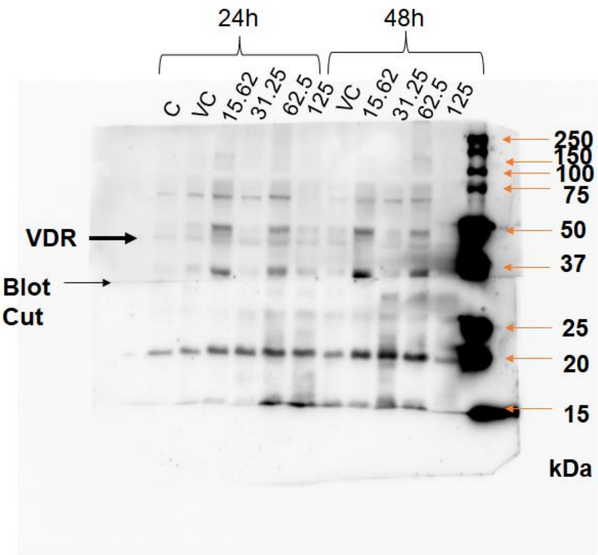

Complete blot depicting VDR band

Catalog Number : sc-13133

The protein bands were visualized using ECL (Clarity Western ECL substrate, Biorad 170-5060) followed by capturing the image in a Chemidoc system (Alliance Q9 UVITec Cambridge UK).

HCT 116-p53

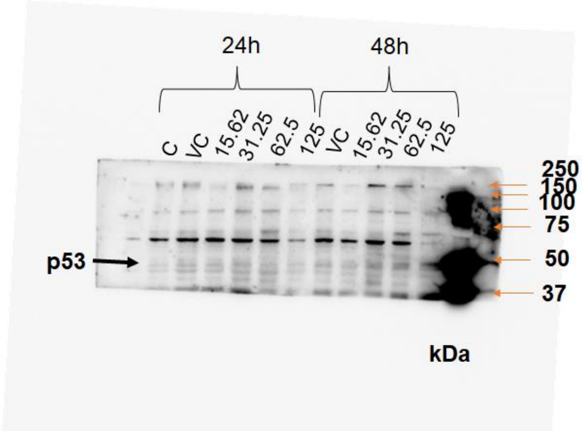

Cut blot depicting p53 band

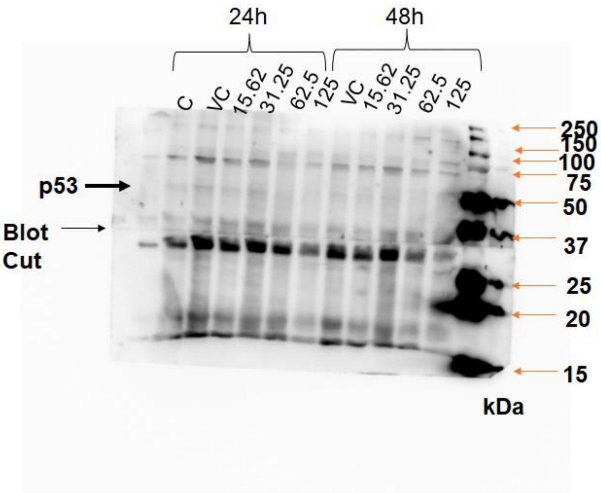

Complete blot depicting p53 band

Catalog Number : 9282

The protein bands were visualized using ECL (Clarity Western ECL substrate, Biorad 170-5060) followed by capturing the image in a Chemidoc system (Alliance Q9 UVITec Cambridge UK).

HCT 116-Bax

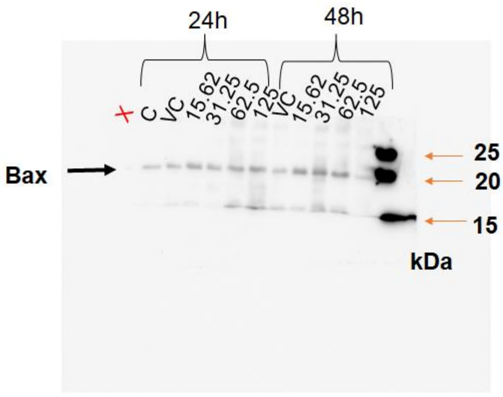

Cut blot depicting Bax band

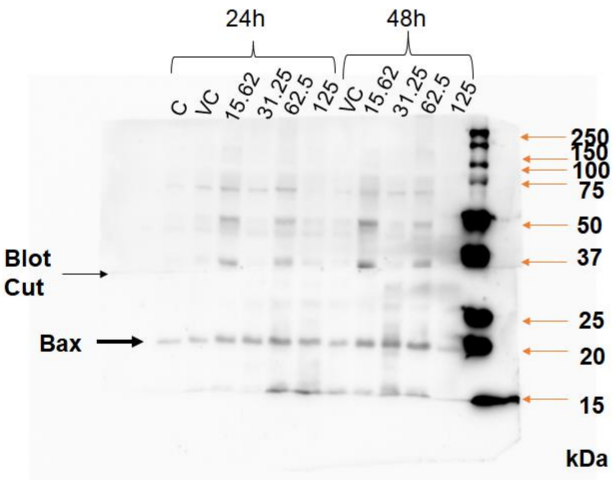

Complete blot depicting Bax band

Catalog Number : 5023s

The protein bands were visualized using ECL (Clarity Western ECL substrate, Biorad 170-5060) followed by capturing the image in a Chemidoc system (Alliance Q9 UVITec Cambridge UK).

HCT 116-Bcl2

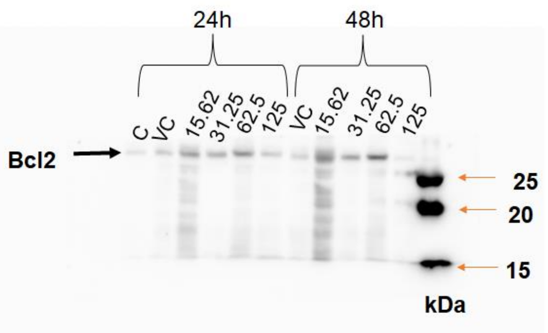

Cut blot depicting Bcl2 band

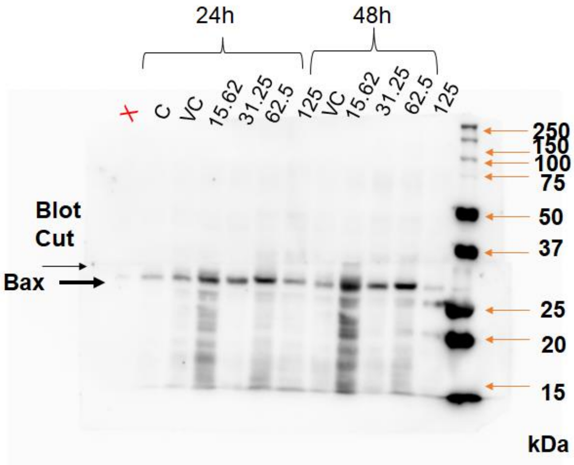

Complete blot depicting Bcl2 band

Catalog Number : PAA778hu01

The protein bands were visualized using ECL (Clarity Western ECL substrate, Biorad 170-5060) followed by capturing the image in a Chemidoc system (Alliance Q9 UVITec Cambridge UK).

HCT 116-Cyclin D1

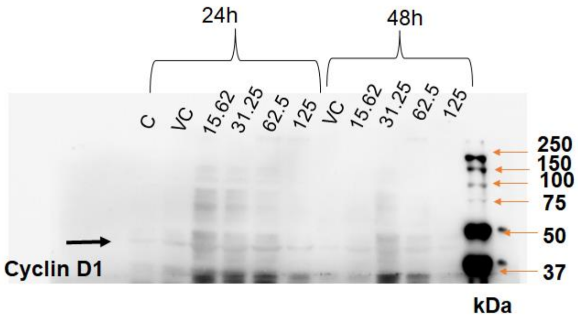

Cut blot depicting Cyclin D1 band

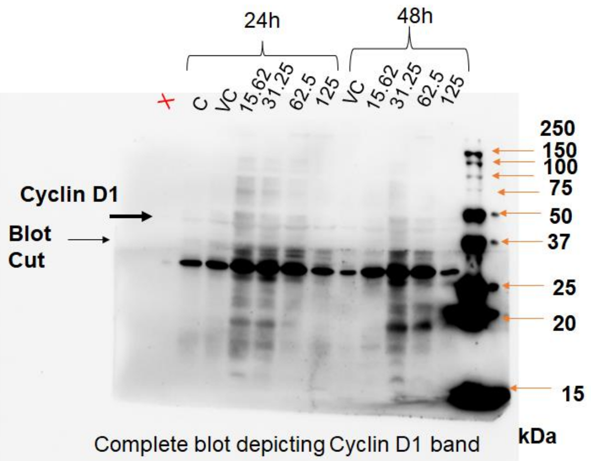

Complete blot depicting Cyclin D1 band

Catalog Number : Sc-8396

The protein bands were visualized using ECL (Clarity Western ECL substrate, Biorad 170-5060) followed by capturing the image in a Chemidoc system (Alliance Q9 UVITec Cambridge UK).

HCT 116-p21

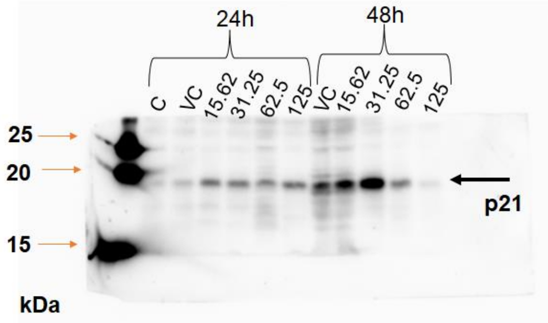

Cut blot depicting p21 band

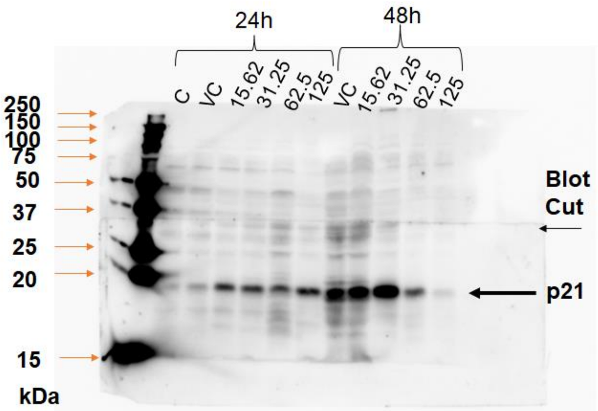

Complete blot depicting p21 band

Catalog Number : sc-6246

The protein bands were visualized using ECL (Clarity Western ECL substrate, Biorad 170-5060) followed by capturing the image in a Chemidoc system (Alliance Q9 UVITec Cambridge UK).

HCT 116-Survivin

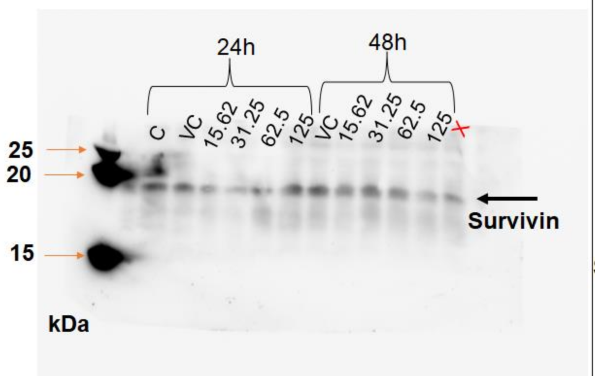

Cut blot depicting Survivin band

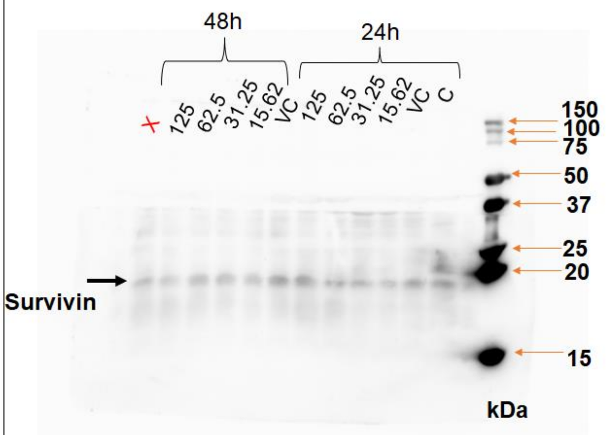

Complete blot depicting Survivin band

Catalog Number : 2803

The protein bands were visualized using ECL (Clarity Western ECL substrate, Biorad 170-5060) followed by capturing the image in a Chemidoc system (Alliance Q9 UVITec Cambridge UK).

HCT 116-Beta Actin

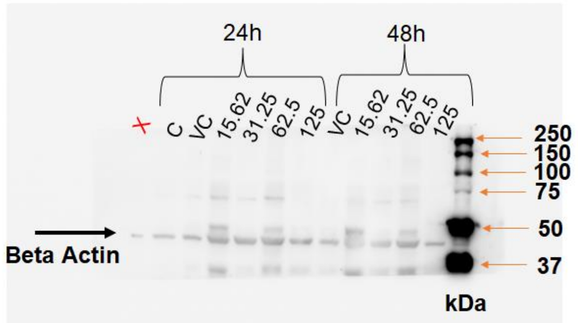

Cut blot depicting Beta Actin band

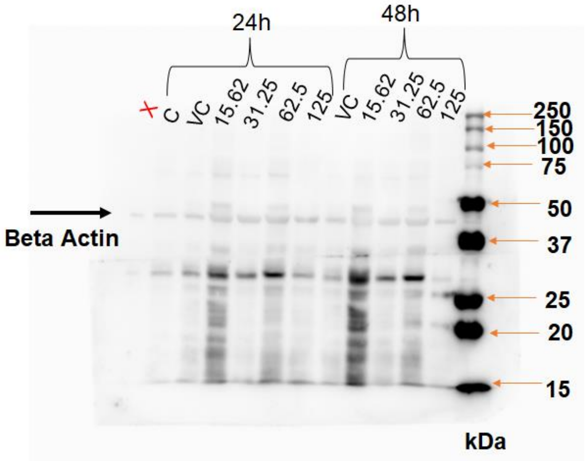

Complete blot depicting Beta Actin band

Catalog Number : CAB340Mi22

The protein bands were visualized using ECL (Clarity Western ECL substrate, Biorad 170-5060) followed by capturing the image in a Chemidoc system (Alliance Q9 UVITec Cambridge UK).
